# Supplementary material for: Use Internet search data to accurately track state level influenza epidemics
Source: Sci Rep. 2021 Feb 17;11:4023. doi: 10.1038/s41598-021-83084-5 (PMC7889878; doi:10.1038/s41598-021-83084-5)
Supplement: Supplementary file 1 — Supplementary Tables [file 41598_2021_83084_MOESM1_ESM.pdf]

Supporting Information for
Use Internet Search Data to Accurately Track State Level Influenza Epidemics

Shihao Yang, Shaoyang Ning, S. C. Kou
Correspondence to: kou@stat.harvard.edu

This PDF file includes:

- Supplementary Text
- Supplementary Figs. S1 to S51
- Supplementary Tables S1 to S55

This Supplementary Material is organized as following: (1) the detailed calculation procedure for the multiple correlation behind the stand-alone modeling of HI, AK, ND, VT, MT, ME, and SD is presented; (2) a table for the confidence interval coverage is presented; (3) all the Google query terms used in this study are listed; (4) Google Trends data quality at different geographic area is studied; (5) full comparison to another Google-search-based benchmark method is presented; (6) detailed estimation results for each of 51 studied states/district/city are reported in tables and plotted in figures.

Multiple correlation

For each state, the multiple correlation of its flu activity level to the other states', other regions' and the national flu activity levels is calculated as follows. First, the states of HI and AK are excluded because they are not part of the contiguous US; the state of FL is excluded because FL data is not available from CDC. Then for the in-sample time period of 2010-10-09 to 2014-09-27, we regress each state's %ILI to (i) all other 48 states' %ILI (including DC and NYC but excluding FL, HI, and AK), (ii) all the other 9 regions' %ILI (i.e., regions other than the one that the specific state belongs to), and (iii) the national %ILI. After the regression, we obtain the R-squared, which is the square of multiple correlation. The five states with the lowest multiple correlations are ND, VT, MT, ME, and SD. We, therefore, would not use spatial pooling on HI, AK, ND, VT, MT, ME, and SD. Instead, we only use the state-specific data together with national level data for cross-resolution boosting on those seven aforementioned states in the second step of ARGOX.

Confidence interval coverage

We study the goodness of our confidence intervals by examining its actual coverage (coverage of the actual %ILI released by CDC weeks later). The result is shown in Table S1. In general, the coverage of 95% confidence interval is quite close to the nominal value, suggesting that our model quantifies the uncertainty reasonably well.

AL	AK	AZ	AR	CA	CO	CT	DE	DC	GA
0.919	0.916	0.909	0.930	0.944	0.905	0.909	0.930	0.923	0.926
HI	ID	IL	IN	IA	KS	KY	LA	ME	MD
0.947	0.958	0.930	0.926	0.926	0.940	0.867	0.916	0.916	0.947
MA	MI	MN	MS	MO	MT	NE	NV	NH	NJ
0.937	0.947	0.940	0.930	0.923	0.937	0.909	0.916	0.898	0.930
NM	NY	NC	ND	OH	OK	OR	PA	RI	SC
0.933	0.902	0.951	0.944	0.926	0.937	0.926	0.930	0.909	0.926
SD	TN	TX	UT	VT	VA	WA	WV	WI	WY
0.940	0.919	0.926	0.898	0.947	0.951	0.905	0.909	0.937	0.874
NYC									
0.912									

Table S1. The actual coverage of the confidence intervals by ARGOX for 51 states/district/city. The coverage is for 95% nominal confidence level. The average coverage over all the 51 states/district/city is 92.5%.

Query terms for Google Trends

Table S2 lists all query terms/phrases used in this study. Most of them are taken from previous studies[9,22] with a few additional terms identified through "Related topics" and "Related queries" from Google Trends when search for flu-related information.

Table S2. All search query terms used in this study. The last 21 terms separated by a horizontal line from the first 140 terms are new "Related topics" and "Related queries" identified from Google Trends.

flu incubation	flu incubation period	influenza type a	symptoms of the flu
flu symptoms	influenza symptoms	flu contagious	influenza a
a influenza	symptoms of flu	flu duration	influenza incubation
type a influenza	flu treatment	symptoms of influenza	influenza contagious
flu in children	cold or flu	symptoms of bronchitis	flu recovery
tessalon	influenza incubation period	symptoms of pneumonia	tussionex
signs of the flu	flu treatments	remedies for the flu	walking pneumonia
flu test	tussin	upper respiratory	respiratory flu
acute bronchitis	bronchitis	sinus infections	flu relief
painful cough	how long does the flu last	flu cough	sinus
expectorant	strep	strep throat	influenza treatment
flu reports	flu remedy	robitussin	rapid flu
treatment for the flu	chest cold	cough fever	oscillococcinum
flu fever	treat the flu	how to treat the flu	over the counter flu
how long is the flu	flu medicine	flu or cold	normal body
is flu contagious	treat flu	body temperature	reduce fever
flu vs cold	how long is the flu contagious	fever reducer	get over the flu
treating flu	having the flu	treatment for flu	human temperature
dangerous fever	the flu	remedies for flu	influenza a and b
contagious flu	fever flu	flu remedies	how long is flu contagious
cold vs flu	braun thermoscan	fever cough	signs of flu
how long does flu last	normal body temperature	get rid of the flu	i have the flu
taking temperature	flu versus cold	how long flu	flu germs
flu and cold	thermoscan	flu complications	high fever
flu children	the flu virus	how to treat flu	pneumonia
flu headache	ear thermometer	how to get rid of the flu	flu how long
cold and flu	over the counter flu medicine	treating the flu	flu care
how long contagious	fight the flu	reduce a fever	cure the flu
medicine for flu	flu length	cure flu	exposed to flu
low body	early flu symptoms	flu report	incubation period for flu
break a fever	flu contagious period	cold versus flu	what to do if you have the flu
medicine for the flu	flu and fever	flu lasts	incubation period for the flu
do i have the flu	type a flu symptoms	flu texas	how long am i contagious with the flu
how to break a fever	fever breaks	type a flu	how to bring a fever down
how to treat the flu at home	flu how long are you contagious	flu a symptoms	flu
Influenza vaccine	Influenza	Fever	Influenza A virus
Influenza B virus	Common cold	Cough	Sore throat
Virus	Avian influenza	Spanish flu	Headache
Nausea	Flu season	Oseltamivir	Nasal congestion
Canine influenza	Rapid influenza diagnostic test	Theraflu	Dextromethorphan
Rhinorrhea			

Google Trends data quality

As stated at `trends.google.com`, the numbers in Google Trends "represent search interest relative to the highest point on the chart for the given region and time; a value of 100 is the peak popularity for the term; a value of 50 means that the term is half as popular; a score of 0 means there was not enough data for this term." As such, the proportion of zeros in the Google Trends data reflects the data quality: higher proportion of zeros indicates lower quality of Google Trends data. Table S3 summarizes the average proportion of zeros for the query terms listed in Table S2 in each of the geographic areas. As we can see, Google Trends data at the US national level have far fewer zeros than any of the states, implying a significant drop in quality from national-level data to state-level data.

Table S3. Average proportion of zeros in Google Trends data for the query terms in Table S2. Higher proportion of zeros indicates lower quality of Google Trends data since "a score of 0 means there was not enough data for this term" (`trends.google.com`). The proportion of zeros in Google Trends at the US national level is in the upper sub-table, while the proportions of zeros at state/district/city level are in the lower sub-table.

	US National
proportion of zeros	1.37%

AK	AL	AR	AZ	CA	CO	CT	DC	DE	FL
78.33%	46.13%	56.16%	39.53%	13.52%	42.77%	50.99%	63.00%	73.34%	20.93%
GA	HI	IA	ID	IL	IN	KS	KY	LA	MA
29.86%	67.14%	54.90%	64.58%	26.39%	40.78%	55.50%	47.06%	48.70%	37.68%
MD	ME	MI	MN	MO	MS	MT	NC	ND	NE
41.26%	67.47%	31.78%	41.99%	40.67%	56.99%	73.07%	30.69%	75.61%	59.34%
NH	NJ	NM	NV	NY	OH	OK	OR	PA	RI
67.21%	34.86%	63.73%	55.14%	18.92%	30.64%	50.78%	48.99%	30.17%	69.28%
SC	SD	TN	TX	UT	VA	VT	WA	WI	WV
45.97%	74.43%	38.48%	16.77%	52.78%	32.98%	77.42%	37.85%	42.78%	64.07%
WY	NYC								
79.82%	18.25%								

More comparison with the result of Lu et al. (2019)

Lu et al. (2019)[21] proposed another Google-search-based method for state-level influenza tracking, utilizing a network approach. We compare ARGOX with Lu et al. (2019) together with other methods here. The retrospective results of Lu et al. (2019) are available for seasons 2014-15, 2015-16, and 2016-17, and it only studied 37 selected states, which are: AK, AL, AR, AZ, DE, GA, ID, KS, KY, LA, MA, MD, ME, MI, MN, NC, ND, NE, NH, NJ, NM, NV, NY, OH, OR, PA, RI, SC, SD, TN, TX, UT, VA, VT, WA, WI, WV. For completeness, ARGOX, VAR, GFT, and the naive method are compared here for the same period and for the same 37 states. Overall ARGOX takes the lead in this subset of 37 states for all three seasons of 2014-15, 2015-16 and 2016-17.

	Overall '14-'17	'14-'15	'15-'16	'16-'17
MSE				
ARGOX	**0.269**	**0.406**	**0.163**	**0.339**
VAR	0.873	1.234	0.503	1.214
GFT	–	1.464	–	–
Lu et al. (2019)	0.418	0.467	0.528	0.544
naive	0.383	0.618	0.201	0.471
Correlation				
ARGOX	**0.919**	0.914	**0.836**	**0.890**
VAR	0.808	0.809	0.684	0.754
GFT	–	**0.915**	–	–
Lu et al. (2019)	0.912	0.912	0.808	0.858
naive	0.894	0.880	0.806	0.860

Table S4. Comparison of different methods for state-level %ILI estimation, averaging over the 37 states, for the period of 2014 to 2017, due to the availability of Lu et al. (2019). The MSE and Correlation are reported. The method with the best performance is highlighted in boldface for each metric in each period. Methods considered here include ARGOX, VAR, GFT, Lu et al. (2019), and the naive method.

Detailed estimation results for each state/district/city

	Whole period '14-'20	Overall '14-'17	'14-'15	'15-'16	'16-'17	'17-'18	'18-'19	'19-'20
MSE								
ARGOX	**0.726**	**1.121**	2.819	0.369	**0.321**	**0.773**	**0.267**	**0.692**
VAR	1.214	1.378	2.617	0.551	1.226	1.611	1.650	1.553
GFT	–	–	7.441	–	–	–	–	–
Lu et al. (2019)	–	4.875	**2.509**	12.152	3.437	–	–	–
naive	1.233	1.792	4.594	**0.349**	0.691	1.678	0.616	1.156
MAE								
ARGOX	**0.469**	**0.608**	**1.022**	0.472	**0.444**	**0.440**	**0.328**	**0.635**
VAR	0.707	0.801	1.055	0.629	0.861	0.780	0.788	0.912
GFT	–	–	1.750	–	–	–	–	–
Lu et al. (2019)	–	–	–	–	–	–	–	–
naive	0.593	0.709	1.200	**0.444**	0.639	0.738	0.573	0.812
Correlation								
ARGOX	**0.954**	**0.928**	0.917	0.826	0.955	**0.974**	**0.969**	**0.929**
VAR	0.929	0.912	0.924	0.844	0.883	0.959	0.906	0.846
GFT	–	–	**0.963**	–	–	–	–	–
Lu et al. (2019)	–	0.885	0.924	0.683	**0.969**	–	–	–
naive	0.923	0.888	0.867	**0.851**	0.906	0.942	0.925	0.883

Table S5. Comparison of different methods for state-level %ILI estimation in Alabama (AL). The MSE, MAE, and correlation are reported. The method with the best performance is highlighted in boldface for each metric in each period.

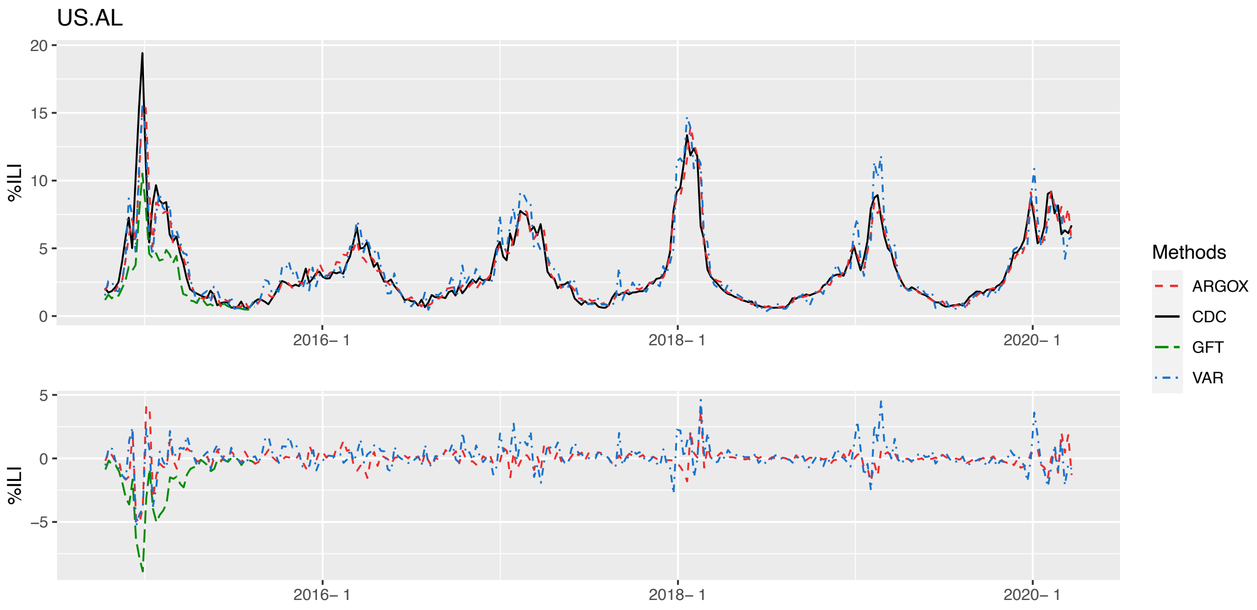

Figure S1. Plots of the %ILI estimates (top) and the estimation errors (bottom) for Alabama (AL).

	Whole period ’14-’20	Overall ’14-’17	’14-’15	’15-’16	’16-’17	’17-’18	’18-’19	’19-’20
MSE								
ARGOX	**0.911**	**0.475**	**0.381**	0.695	**0.490**	**0.371**	**1.217**	**4.487**
VAR	4.902	4.659	6.417	4.153	5.154	10.475	2.379	8.727
GFT	–	–	0.918	–	–	–	–	–
Lu et al. (2019)	–	0.510	0.450	**0.692**	0.602	–	–	–
naive	0.996	0.534	0.395	0.710	0.612	0.404	1.269	4.898
MAE								
ARGOX	**0.591**	**0.477**	**0.459**	**0.565**	**0.520**	**0.493**	**0.768**	**1.310**
VAR	1.338	1.299	1.332	1.331	1.546	2.094	1.128	1.768
GFT	–	–	0.780	–	–	–	–	–
Lu et al. (2019)	–	–	–	–	–	–	–	–
naive	0.619	0.510	0.461	0.596	0.604	0.504	0.821	1.326
Correlation								
ARGOX	**0.872**	**0.805**	0.746	0.815	**0.800**	**0.923**	0.879	**0.564**
VAR	0.593	0.291	0.166	0.363	0.227	0.380	0.754	0.457
GFT	–	–	0.638	–	–	–	–	–
Lu et al. (2019)	–	0.781	0.723	0.815	0.750	–	–	–
naive	0.865	0.797	**0.762**	**0.820**	0.769	0.912	**0.880**	0.555

Table S6. Comparison of different methods for state-level %ILI estimation in Alaska (AK). The MSE, MAE, and correlation are reported. The method with the best performance is highlighted in boldface for each metric in each period.

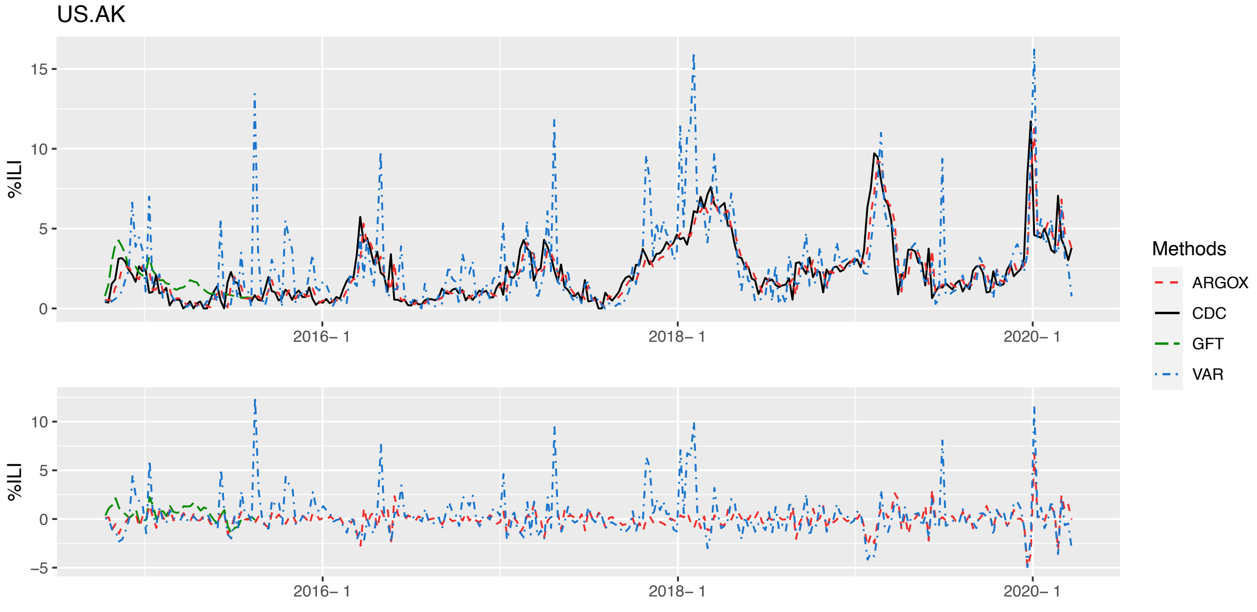

Figure S2. Plots of the %ILI estimates (top) and the estimation errors (bottom) for Alaska (AK).

	Whole period ’14-’20	Overall ’14-’17	’14-’15	’15-’16	’16-’17	’17-’18	’18-’19	’19-’20
MSE								
ARGOX	**0.151**	**0.093**	0.064	**0.157**	0.098	**0.418**	**0.094**	**0.208**
VAR	0.663	0.188	0.062	0.270	0.362	3.360	0.262	0.781
GFT	–	–	1.213	–	–	–	–	–
Lu et al. (2019)	–	0.126	**0.052**	0.237	0.139	–	–	–
naive	0.185	0.095	0.070	0.161	**0.092**	0.602	0.132	0.276
MAE								
ARGOX	**0.282**	**0.218**	**0.174**	**0.291**	0.254	**0.480**	**0.257**	**0.385**
VAR	0.459	0.319	0.199	0.376	0.497	0.999	0.403	0.543
GFT	–	–	0.940	–	–	–	–	–
Lu et al. (2019)	–	–	–	–	–	–	–	–
naive	0.297	0.230	0.198	0.293	**0.254**	0.529	0.269	0.387
Correlation								
ARGOX	**0.951**	**0.957**	0.957	**0.944**	0.861	**0.927**	**0.925**	**0.867**
VAR	0.835	0.923	**0.963**	0.921	0.555	0.788	0.812	0.699
GFT	–	–	0.934	–	–	–	–	–
Lu et al. (2019)	–	0.949	0.960	0.930	**0.896**	–	–	–
naive	0.941	0.956	0.949	0.939	0.865	0.886	0.901	0.810

Table S7. Comparison of different methods for state-level %ILI estimation in Arizona (AZ). The MSE, MAE, and correlation are reported. The method with the best performance is highlighted in boldface for each metric in each period.

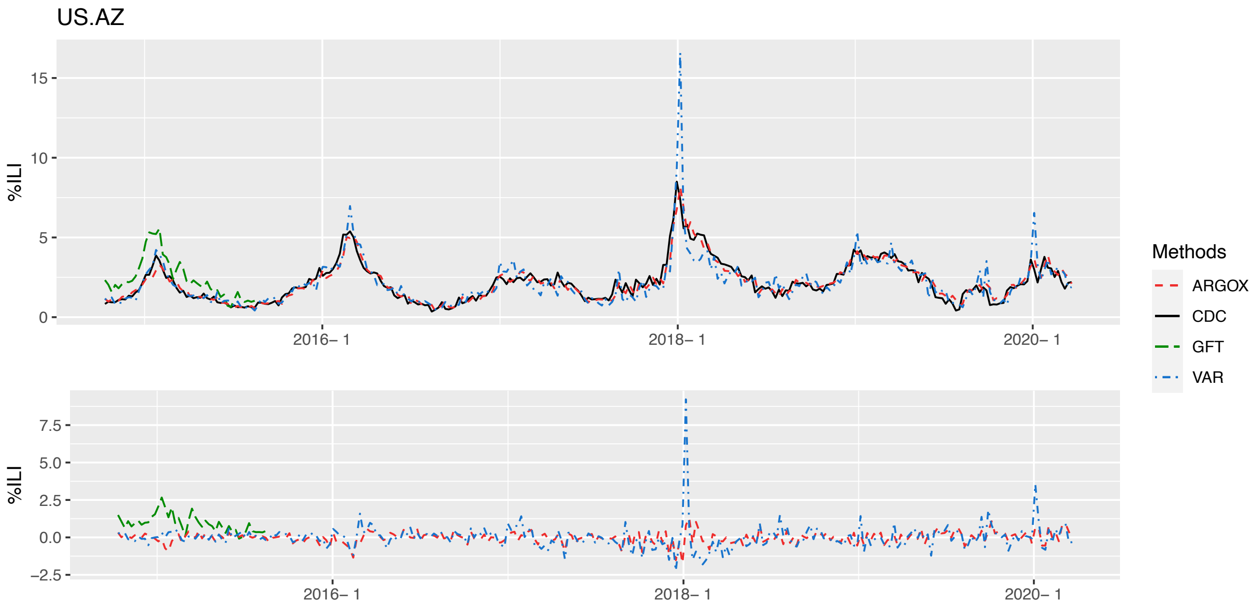

Figure S3. Plots of the %ILI estimates (top) and the estimation errors (bottom) for Arizona (AZ).

	Whole period '14-'20	Overall '14-'17	'14-'15	'15-'16	'16-'17	'17-'18	'18-'19	'19-'20
MSE								
ARGOX	**0.786**	**0.585**	**1.024**	**0.286**	**0.677**	**0.849**	**0.551**	**3.791**
VAR	2.487	1.529	2.202	0.879	2.109	9.018	2.619	3.827
GFT	–	–	1.820	–	–	–	–	–
Lu et al. (2019)	–	0.679	1.325	0.341	–	–	–	–
naive	1.201	0.922	1.570	0.418	1.166	2.024	0.974	4.553
MAE								
ARGOX	**0.532**	**0.457**	**0.586**	**0.389**	**0.549**	**0.673**	**0.550**	1.424
VAR	0.894	0.806	0.964	0.686	1.075	1.584	1.125	**1.275**
GFT	–	–	1.153	–	–	–	–	–
Lu et al. (2019)	–	–	–	–	–	–	–	–
naive	0.647	0.548	0.677	0.472	0.708	1.016	0.729	1.523
Correlation								
ARGOX	**0.937**	**0.940**	0.927	**0.904**	**0.940**	**0.972**	**0.951**	**0.667**
VAR	0.856	0.860	0.907	0.685	0.783	0.842	0.884	0.633
GFT	–	–	**0.974**	–	–	–	–	–
Lu et al. (2019)	–	0.928	0.909	0.875	–	–	–	–
naive	0.906	0.906	0.889	0.866	0.893	0.920	0.903	0.620

Table S8. Comparison of different methods for state-level %ILI estimation in Arkansas (AR). The MSE, MAE, and correlation are reported. The method with the best performance is highlighted in boldface for each metric in each period.

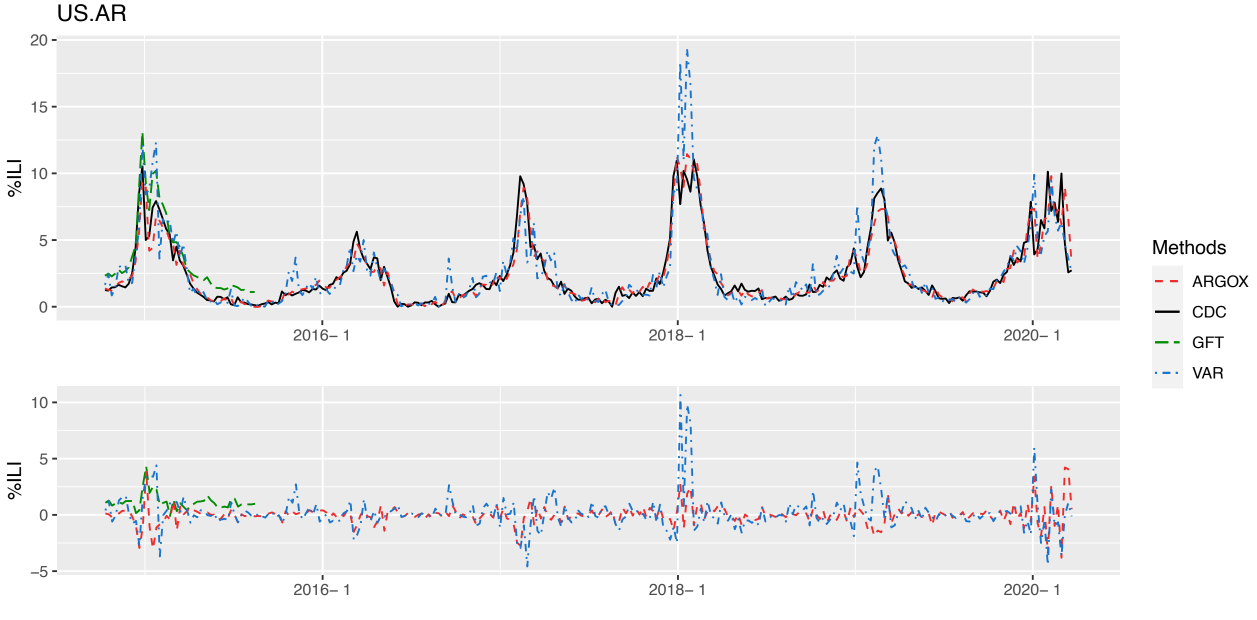

Figure S4. Plots of the %ILI estimates (top) and the estimation errors (bottom) for Arkansas (AR).

	Whole period ’14-’20	Overall ’14-’17	’14-’15	’15-’16	’16-’17	’17-’18	’18-’19	’19-’20
MSE								
ARGOX	**0.076**	**0.067**	**0.081**	**0.078**	0.068	**0.155**	**0.047**	**0.195**
VAR	0.169	0.162	0.172	0.190	0.190	0.409	0.093	0.257
GFT	–	–	0.266	–	–	–	–	–
Lu et al. (2019)	–	–	–	–	–	–	–	–
naive	0.123	0.092	0.124	0.125	**0.064**	0.398	0.069	0.222
MAE								
ARGOX	**0.187**	**0.186**	**0.199**	**0.218**	**0.192**	**0.250**	**0.172**	**0.314**
VAR	0.288	0.284	0.272	0.320	0.310	0.457	0.255	0.379
GFT	–	–	0.388	–	–	–	–	–
Lu et al. (2019)	–	–	–	–	–	–	–	–
naive	0.224	0.214	0.233	0.277	0.194	0.381	0.220	0.330
Correlation								
ARGOX	**0.971**	**0.962**	**0.966**	**0.940**	**0.926**	**0.958**	**0.958**	**0.950**
VAR	0.941	0.938	0.949	0.908	0.872	0.926	0.924	0.931
GFT	–	–	0.927	–	–	–	–	–
Lu et al. (2019)	–	–	–	–	–	–	–	–
naive	0.952	0.947	0.944	0.907	0.917	0.892	0.940	0.943

Table S9. Comparison of different methods for state-level %ILI estimation in California (CA). The MSE, MAE, and correlation are reported. The method with the best performance is highlighted in boldface for each metric in each period.

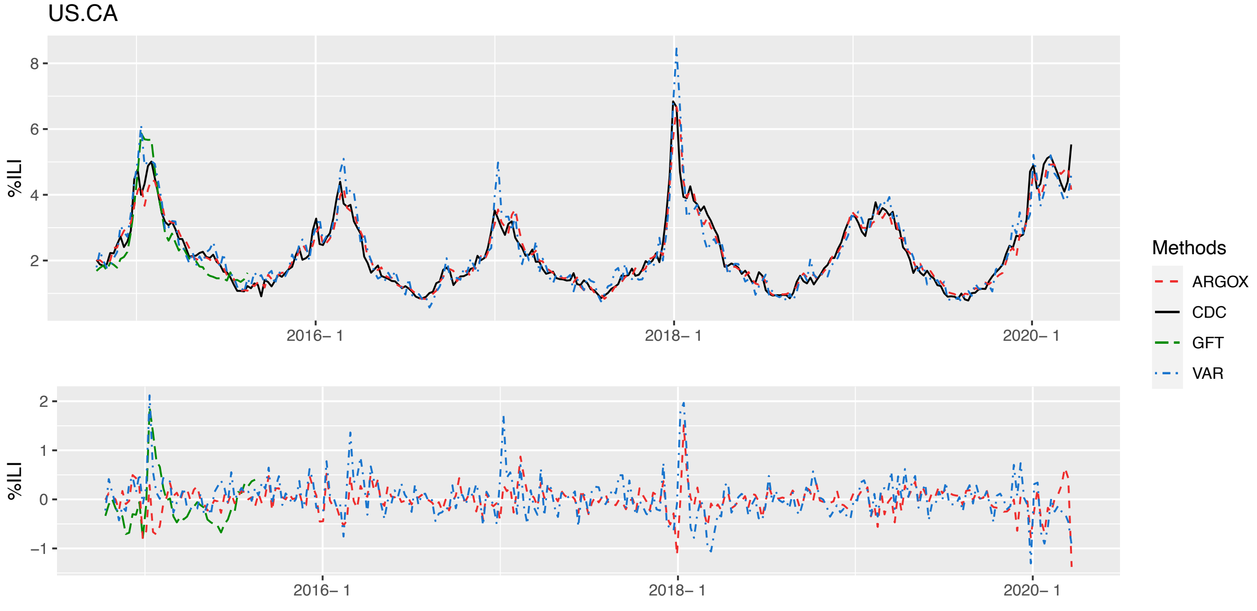

Figure S5. Plots of the %ILI estimates (top) and the estimation errors (bottom) for California (CA).

	Whole period '14-'20	Overall '14-'17	'14-'15	'15-'16	'16-'17	'17-'18	'18-'19	'19-'20
MSE								
ARGOX	**0.178**	**0.153**	**0.166**	**0.028**	**0.343**	**0.047**	0.624	**0.196**
VAR	0.770	0.456	0.514	0.070	1.042	0.814	1.971	2.211
GFT	–	–	0.585	–	–	–	–	–
Lu et al. (2019)	–	–	–	–	–	–	–	–
naive	0.225	0.201	0.204	0.043	0.470	0.071	**0.525**	0.533
MAE								
ARGOX	**0.272**	**0.241**	**0.236**	**0.119**	**0.430**	**0.161**	0.635	**0.332**
VAR	0.531	0.374	0.363	0.204	0.676	0.578	1.062	1.085
GFT	–	–	0.420	–	–	–	–	–
Lu et al. (2019)	–	–	–	–	–	–	–	–
naive	0.310	0.287	0.279	0.166	0.506	0.213	**0.562**	0.534
Correlation								
ARGOX	**0.972**	**0.864**	**0.927**	**0.926**	**0.484**	**0.979**	**0.910**	**0.975**
VAR	0.888	0.665	0.754	0.824	0.461	0.811	0.661	0.784
GFT	–	–	0.850	–	–	–	–	–
Lu et al. (2019)	–	–	–	–	–	–	–	–
naive	0.964	0.832	0.908	0.885	0.374	0.961	0.904	0.930

Table S10. Comparison of different methods for state-level %ILI estimation in Colorado (CO). The MSE, MAE, and correlation are reported. The method with the best performance is highlighted in boldface for each metric in each period.

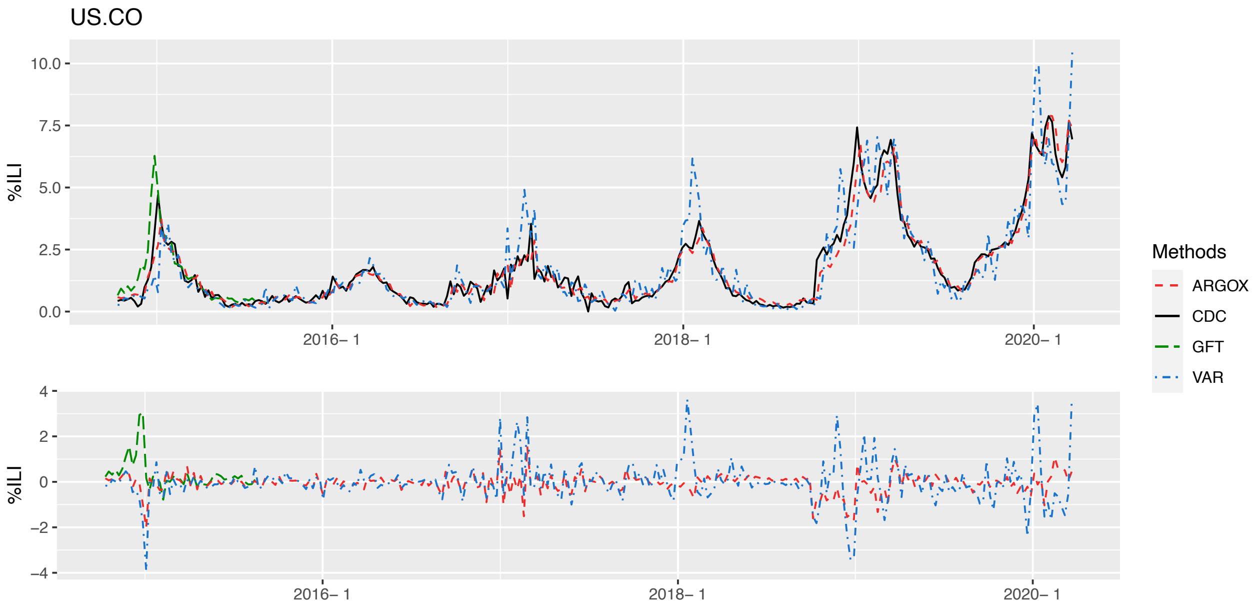

Figure S6. Plots of the %ILI estimates (top) and the estimation errors (bottom) for Colorado (CO).

	Whole period '14-'20	Overall '14-'17	'14-'15	'15-'16	'16-'17	'17-'18	'18-'19	'19-'20
MSE								
ARGOX	**0.281**	**0.228**	0.411	0.222	**0.145**	**0.283**	**0.175**	**0.462**
VAR	3.626	5.482	15.079	1.046	0.512	3.095	1.515	3.967
GFT	–	–	1.944	–	–	–	–	–
Lu et al. (2019)	–	–	–	–	–	–	–	–
naive	0.330	0.230	**0.349**	**0.213**	0.242	0.399	0.243	0.636
MAE								
ARGOX	**0.359**	0.314	0.410	0.365	**0.300**	**0.388**	**0.324**	**0.439**
VAR	0.891	0.925	1.584	0.845	0.584	1.090	0.910	1.395
GFT	–	–	0.999	–	–	–	–	–
Lu et al. (2019)	–	–	–	–	–	–	–	–
naive	0.375	**0.305**	**0.323**	**0.360**	0.379	0.458	0.367	0.578
Correlation								
ARGOX	**0.962**	0.942	0.918	0.882	**0.955**	**0.956**	**0.953**	**0.976**
VAR	0.699	0.443	0.222	0.674	0.855	0.785	0.767	0.822
GFT	–	–	0.774	–	–	–	–	–
Lu et al. (2019)	–	–	–	–	–	–	–	–
naive	0.955	**0.942**	**0.928**	**0.887**	0.925	0.938	0.934	0.960

Table S11. Comparison of different methods for state-level %ILI estimation in Connecticut (CT). The MSE, MAE, and correlation are reported. The method with the best performance is highlighted in boldface for each metric in each period.

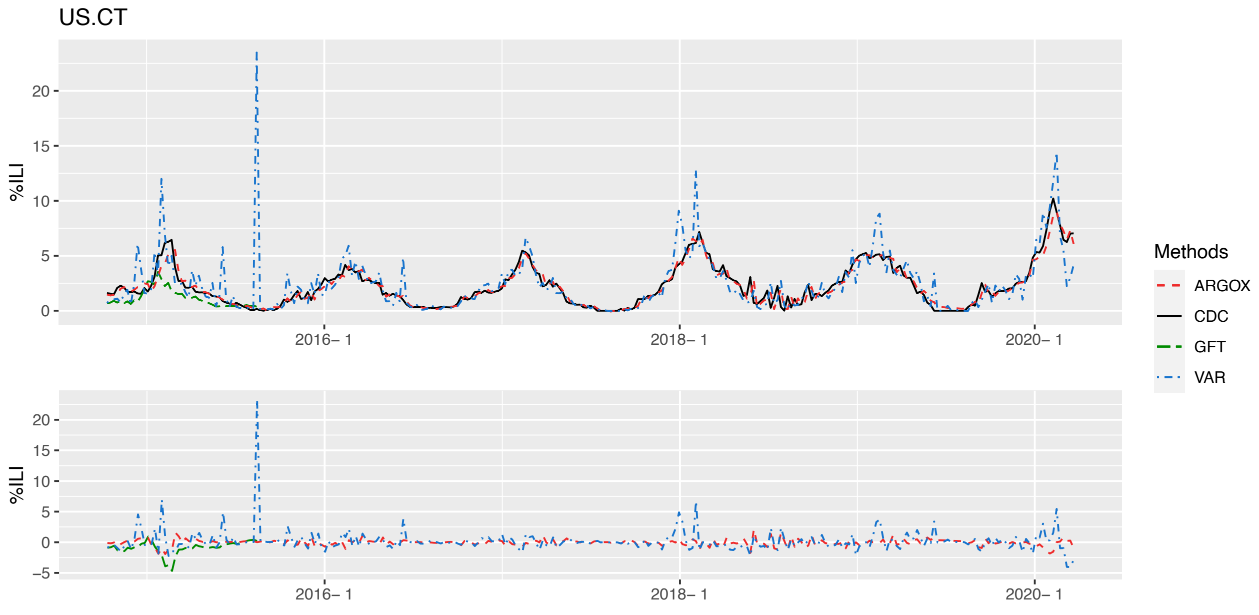

Figure S7. Plots of the %ILI estimates (top) and the estimation errors (bottom) for Connecticut (CT).

	Whole period '14-'20	Overall '14-'17	'14-'15	'15-'16	'16-'17	'17-'18	'18-'19	'19-'20
MSE								
ARGOX	**0.187**	0.126	0.297	**0.062**	**0.041**	**0.445**	**0.071**	**0.725**
VAR	0.644	0.313	0.495	0.116	0.486	2.306	0.239	2.213
GFT	–	–	3.978	–	–	–	–	–
Lu et al. (2019)	–	0.163	0.377	0.073	0.053	–	–	–
naive	0.211	**0.107**	**0.231**	0.066	0.053	0.598	0.086	0.881
MAE								
ARGOX	**0.224**	0.189	0.266	0.188	**0.156**	**0.340**	0.209	**0.600**
VAR	0.359	0.277	0.333	0.230	0.388	0.673	0.340	0.994
GFT	–	–	1.783	–	–	–	–	–
Lu et al. (2019)	–	–	–	–	–	–	–	–
naive	0.232	**0.169**	**0.217**	**0.173**	0.173	0.455	**0.208**	0.674
Correlation								
ARGOX	**0.874**	0.824	0.813	0.832	**0.769**	**0.889**	**0.899**	**0.781**
VAR	0.748	0.718	0.760	0.659	0.730	0.848	0.779	0.457
GFT	–	–	0.747	–	–	–	–	–
Lu et al. (2019)	–	0.843	0.841	**0.840**	0.614	–	–	–
naive	0.864	**0.860**	**0.864**	0.832	0.739	0.850	0.882	0.748

Table S12. Comparison of different methods for state-level %ILI estimation in Delaware (DE). The MSE, MAE, and correlation are reported. The method with the best performance is highlighted in boldface for each metric in each period.

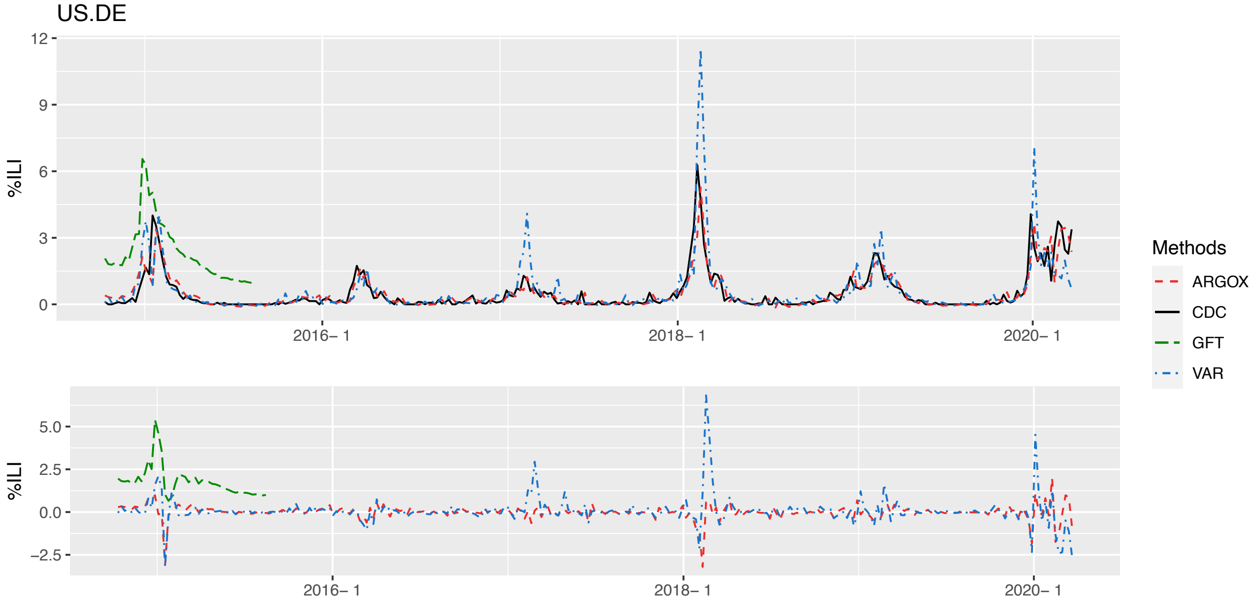

Figure S8. Plots of the %ILI estimates (top) and the estimation errors (bottom) for Delaware (DE).

	Whole period '14-'20	Overall '14-'17	'14-'15	'15-'16	'16-'17	'17-'18	'18-'19	'19-'20
MSE								
ARGOX	**2.011**	**2.806**	**2.875**	**2.634**	**4.192**	1.531	**0.216**	**0.516**
VAR	15.898	10.397	5.739	15.635	17.804	14.672	7.150	1.439
GFT	–	–	25.894	–	–	–	–	–
Lu et al. (2019)	–	–	–	–	–	–	–	–
naive	2.081	2.907	3.124	2.787	4.811	**0.695**	0.280	0.689
MAE								
ARGOX	0.968	1.227	**1.195**	1.289	**1.418**	1.012	**0.320**	**0.568**
VAR	2.126	2.249	1.741	2.723	3.370	2.132	1.775	0.921
GFT	–	–	4.759	–	–	–	–	–
Lu et al. (2019)	–	–	–	–	–	–	–	–
naive	**0.888**	**1.194**	1.297	**1.277**	1.509	**0.444**	0.342	0.650
Correlation								
ARGOX	0.873	0.820	**0.828**	0.786	**0.609**	0.666	**0.677**	**0.794**
VAR	0.554	0.605	0.602	0.580	0.475	0.602	0.243	0.656
GFT	–	–	0.728	–	–	–	–	–
Lu et al. (2019)	–	–	–	–	–	–	–	–
naive	**0.873**	**0.828**	0.810	**0.810**	0.562	**0.728**	0.605	0.741

Table S13. Comparison of different methods for state-level %ILI estimation in District of Columbia (DC). The MSE, MAE, and correlation are reported. The method with the best performance is highlighted in boldface for each metric in each period.

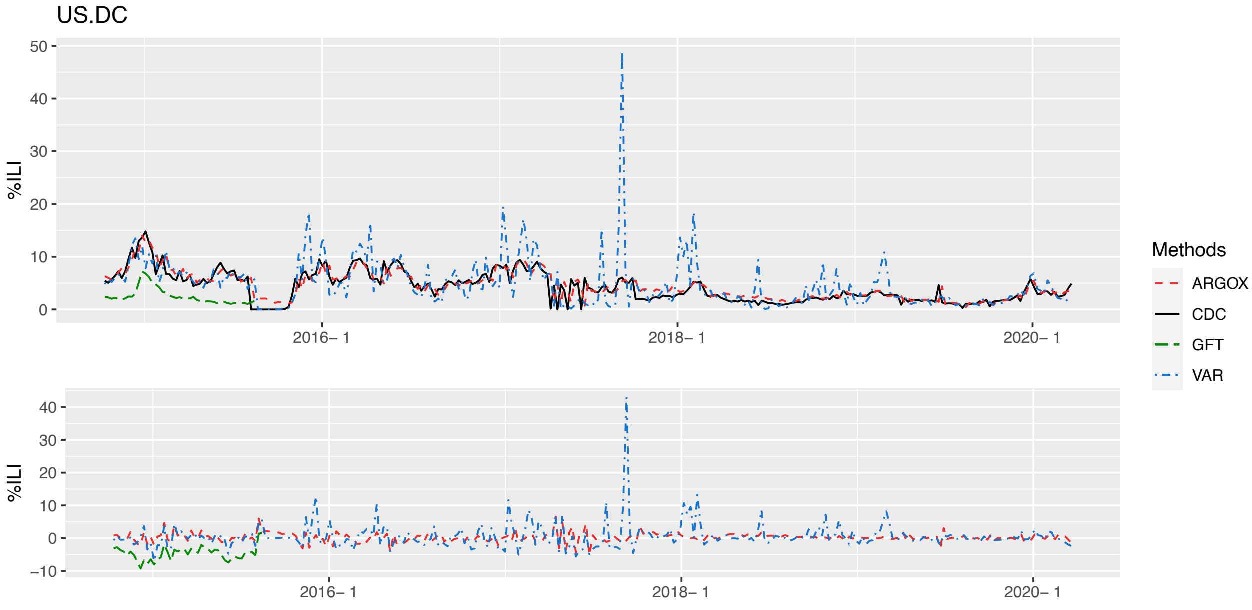

Figure S9. Plots of the %ILI estimates (top) and the estimation errors (bottom) for District of Columbia (DC).

	Whole period '14-'20	Overall '14-'17	'14-'15	'15-'16	'16-'17	'17-'18	'18-'19	'19-'20
MSE								
ARGOX	**0.346**	**0.282**	0.606	**0.096**	**0.192**	**1.057**	**0.145**	**0.742**
VAR	0.715	0.416	0.493	0.469	0.445	1.595	0.986	2.239
GFT	–	–	0.600	–	–	–	–	–
Lu et al. (2019)	–	0.283	**0.408**	0.224	0.347	–	–	–
naive	0.688	0.410	0.888	0.129	0.318	2.188	0.452	2.045
MAE								
ARGOX	**0.337**	**0.313**	**0.378**	**0.242**	**0.373**	**0.617**	**0.271**	**0.658**
VAR	0.527	0.454	0.451	0.484	0.518	0.834	0.666	0.954
GFT	–	–	0.470	–	–	–	–	–
Lu et al. (2019)	–	–	–	–	–	–	–	–
naive	0.442	0.347	0.442	0.275	0.418	0.837	0.488	1.085
Correlation								
ARGOX	**0.970**	0.928	0.882	**0.877**	**0.945**	**0.968**	**0.966**	**0.936**
VAR	0.943	0.889	0.908	0.352	0.870	0.956	0.846	0.869
GFT	–	–	0.901	–	–	–	–	–
Lu et al. (2019)	–	**0.945**	**0.957**	0.711	0.936	–	–	–
naive	0.941	0.893	0.827	0.837	0.909	0.934	0.878	0.830

Table S14. Comparison of different methods for state-level %ILI estimation in Georgia (GA). The MSE, MAE, and correlation are reported. The method with the best performance is highlighted in boldface for each metric in each period.

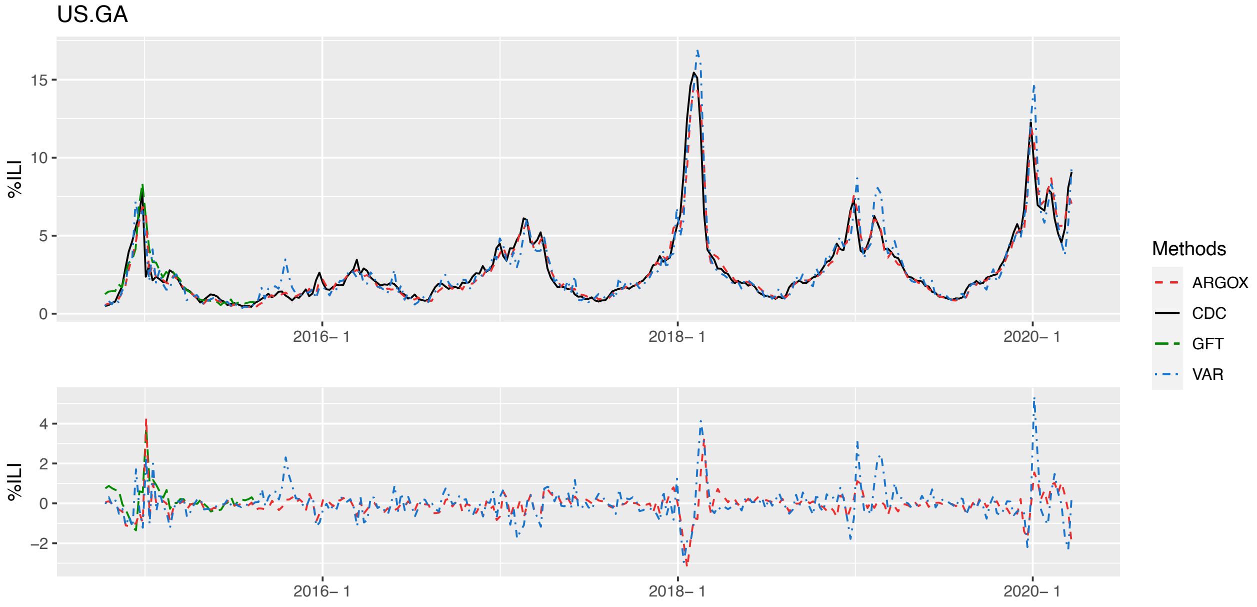

Figure S10. Plots of the %ILI estimates (top) and the estimation errors (bottom) for Georgia (GA).

	Whole period '14-'20	Overall '14-'17	'14-'15	'15-'16	'16-'17	'17-'18	'18-'19	'19-'20
MSE								
ARGOX	**0.821**	1.133	2.491	**0.593**	**0.463**	**1.279**	**0.381**	**0.468**
VAR	2.395	2.945	7.087	1.148	0.893	4.532	0.409	3.337
GFT	–	–	15.289	–	–	–	–	–
Lu et al. (2019)	–	–	–	–	–	–	–	–
naive	0.880	**1.099**	**2.305**	0.619	0.548	1.574	0.500	0.590
MAE								
ARGOX	**0.618**	**0.707**	1.059	**0.619**	**0.558**	**0.832**	**0.469**	**0.528**
VAR	0.989	1.093	1.805	0.813	0.765	1.354	0.513	1.420
GFT	–	–	3.292	–	–	–	–	–
Lu et al. (2019)	–	–	–	–	–	–	–	–
naive	0.662	0.717	**1.037**	0.642	0.609	0.966	0.510	0.608
Correlation								
ARGOX	**0.935**	0.943	0.929	**0.906**	**0.747**	**0.780**	**0.868**	**0.894**
VAR	0.871	0.891	0.870	0.832	0.632	0.681	0.854	0.720
GFT	–	–	0.899	–	–	–	–	–
Lu et al. (2019)	–	–	–	–	–	–	–	–
naive	0.933	**0.946**	**0.936**	0.905	0.716	0.730	0.833	0.874

Table S15. Comparison of different methods for state-level %ILI estimation in Hawaii (HI). The MSE, MAE, and correlation are reported. The method with the best performance is highlighted in boldface for each metric in each period.

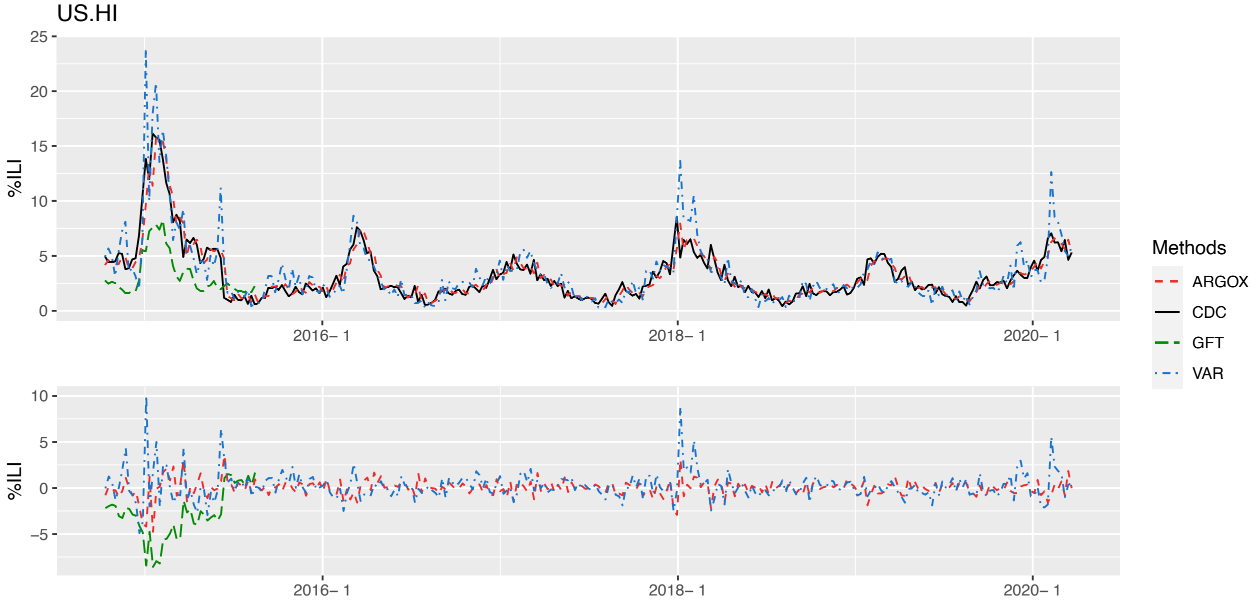

Figure S11. Plots of the %ILI estimates (top) and the estimation errors (bottom) for Hawaii (HI).

	Whole period '14-'20	Overall '14-'17	'14-'15	'15-'16	'16-'17	'17-'18	'18-'19	'19-'20
MSE								
ARGOX	**0.252**	**0.283**	**0.433**	0.250	**0.164**	**0.255**	0.552	0.186
VAR	1.008	1.605	2.637	0.525	0.777	1.020	**0.539**	0.541
GFT	–	–	1.395	–	–	–	–	–
Lu et al. (2019)	–	0.377	0.494	**0.205**	–	–	–	–
naive	0.322	0.369	0.675	0.221	0.228	0.398	0.673	**0.164**
MAE								
ARGOX	**0.351**	**0.402**	**0.473**	0.397	**0.316**	**0.379**	0.512	0.335
VAR	0.605	0.809	1.073	0.578	0.702	0.728	**0.501**	0.557
GFT	–	–	0.965	–	–	–	–	–
Lu et al. (2019)	–	–	–	–	–	–	–	–
naive	0.383	0.452	0.629	**0.375**	0.362	0.469	0.583	**0.333**
Correlation								
ARGOX	**0.919**	0.928	0.939	0.621	**0.803**	**0.874**	0.778	**0.874**
VAR	0.744	0.687	0.732	0.383	0.274	0.816	**0.818**	0.494
GFT	–	–	**0.952**	–	–	–	–	–
Lu et al. (2019)	–	**0.929**	0.931	**0.697**	–	–	–	–
naive	0.900	0.909	0.904	0.692	0.750	0.818	0.754	0.863

Table S16. Comparison of different methods for state-level %ILI estimation in Idaho (ID). The MSE, MAE, and correlation are reported. The method with the best performance is highlighted in boldface for each metric in each period.

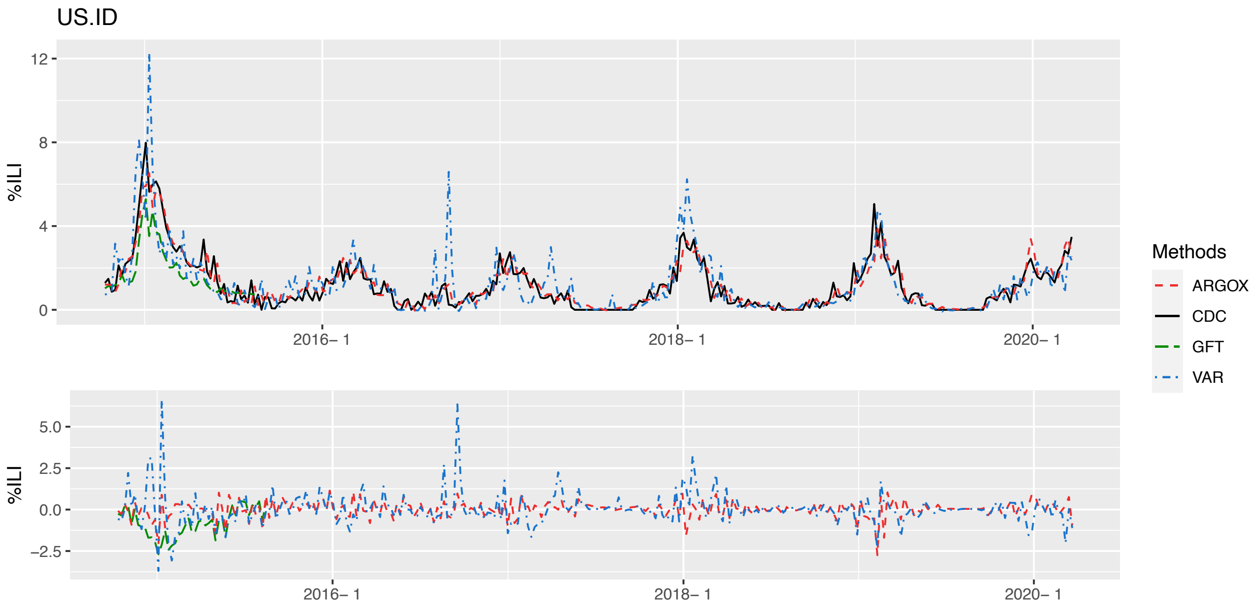

Figure S12. Plots of the %ILI estimates (top) and the estimation errors (bottom) for Idaho (ID).

	Whole period '14-'20	Overall '14-'17	'14-'15	'15-'16	'16-'17	'17-'18	'18-'19	'19-'20
MSE								
ARGOX	**0.085**	**0.079**	**0.156**	**0.050**	**0.046**	**0.106**	**0.077**	**0.291**
VAR	0.203	0.157	0.259	0.068	0.146	0.327	0.122	0.829
GFT	–	–	0.414	–	–	–	–	–
Lu et al. (2019)	–	–	–	–	–	–	–	–
naive	0.163	0.117	0.188	0.073	0.129	0.381	0.155	0.479
MAE								
ARGOX	**0.185**	**0.189**	**0.247**	**0.179**	**0.177**	**0.213**	**0.194**	**0.387**
VAR	0.289	0.285	0.357	0.203	0.292	0.365	0.228	0.680
GFT	–	–	0.574	–	–	–	–	–
Lu et al. (2019)	–	–	–	–	–	–	–	–
naive	0.254	0.230	0.262	0.221	0.268	0.396	0.285	0.523
Correlation								
ARGOX	**0.980**	**0.967**	**0.951**	**0.959**	**0.978**	**0.984**	**0.947**	**0.967**
VAR	0.952	0.941	0.933	0.956	0.931	0.955	0.913	0.905
GFT	–	–	0.947	–	–	–	–	–
Lu et al. (2019)	–	–	–	–	–	–	–	–
naive	0.962	0.951	0.939	0.939	0.936	0.939	0.889	0.945

Table S17. Comparison of different methods for state-level %ILI estimation in Illinois (IL). The MSE, MAE, and correlation are reported. The method with the best performance is highlighted in boldface for each metric in each period.

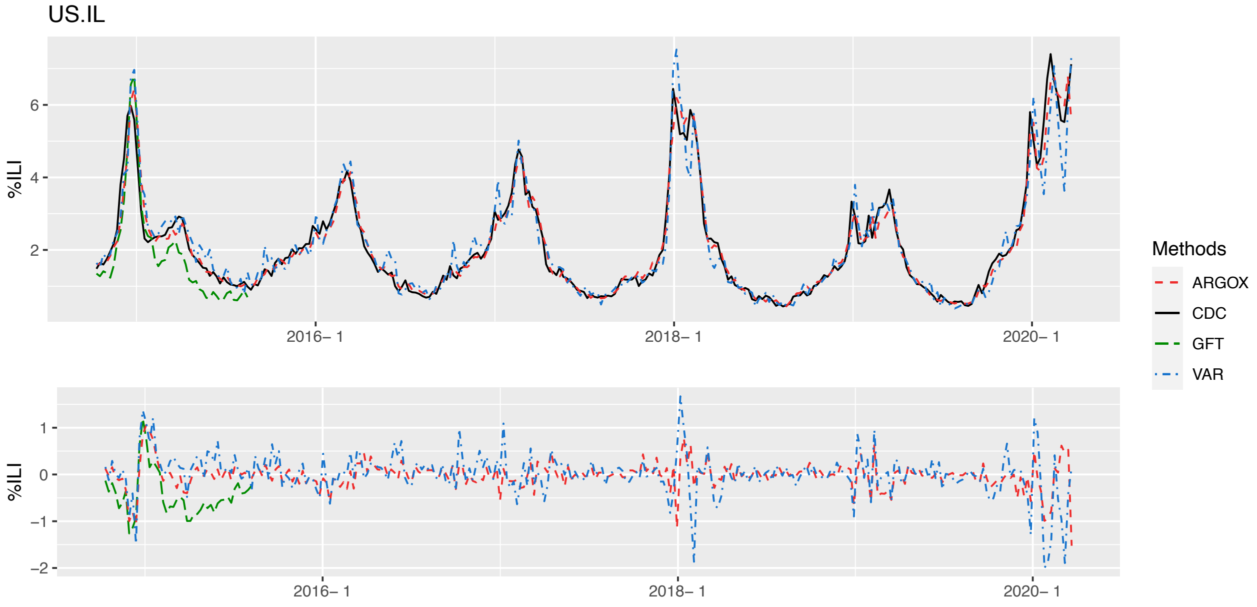

Figure S13. Plots of the %ILI estimates (top) and the estimation errors (bottom) for Illinois (IL).

	Whole period '14-'20	Overall '14-'17	'14-'15	'15-'16	'16-'17	'17-'18	'18-'19	'19-'20
MSE								
ARGOX	**0.326**	**0.304**	0.372	**0.164**	**0.493**	**0.345**	**0.328**	**0.998**
VAR	1.266	0.761	1.410	0.463	0.682	3.456	1.959	2.641
GFT	–	–	**0.220**	–	–	–	–	–
Lu et al. (2019)	–	–	–	–	–	–	–	–
naive	0.537	0.515	0.751	0.315	0.689	0.928	0.613	1.094
MAE								
ARGOX	**0.372**	**0.371**	0.382	**0.310**	**0.485**	**0.435**	**0.408**	**0.576**
VAR	0.636	0.531	0.638	0.499	0.640	1.056	0.940	0.992
GFT	–	–	**0.373**	–	–	–	–	–
Lu et al. (2019)	–	–	–	–	–	–	–	–
naive	0.459	0.464	0.486	0.433	0.596	0.608	0.540	0.685
Correlation								
ARGOX	**0.947**	**0.925**	0.942	**0.878**	**0.900**	**0.972**	**0.922**	**0.860**
VAR	0.857	0.869	0.907	0.707	0.857	0.884	0.668	0.609
GFT	–	–	**0.970**	–	–	–	–	–
Lu et al. (2019)	–	–	–	–	–	–	–	–
naive	0.914	0.877	0.885	0.776	0.865	0.925	0.857	0.840

Table S18. Comparison of different methods for state-level %ILI estimation in Indiana (IN). The MSE, MAE, and correlation are reported. The method with the best performance is highlighted in boldface for each metric in each period.

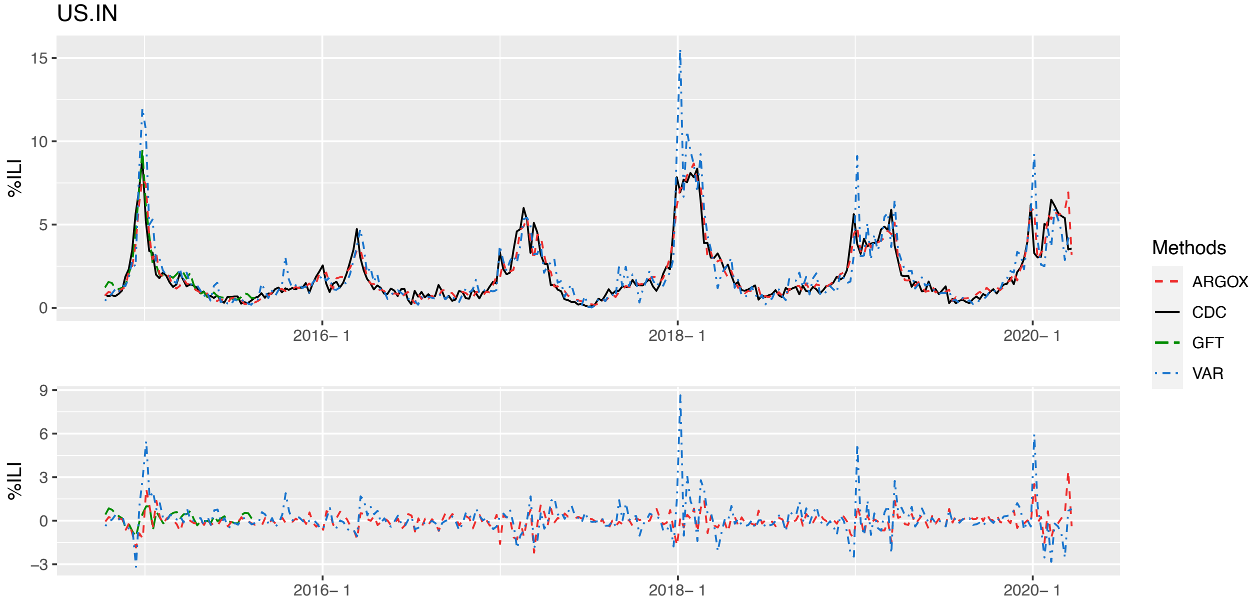

Figure S14. Plots of the %ILI estimates (top) and the estimation errors (bottom) for Indiana (IN).

	Whole period '14-'20	Overall '14-'17	'14-'15	'15-'16	'16-'17	'17-'18	'18-'19	'19-'20
MSE								
ARGOX	**0.166**	**0.182**	0.380	**0.073**	**0.122**	**0.147**	**0.175**	**0.444**
VAR	0.446	0.201	**0.318**	0.096	0.256	0.888	0.430	2.245
GFT	–	–	4.702	–	–	–	–	–
Lu et al. (2019)	–	–	–	–	–	–	–	–
naive	0.219	0.196	0.371	0.087	0.181	0.298	0.229	0.706
MAE								
ARGOX	**0.241**	**0.243**	0.311	**0.225**	**0.250**	**0.280**	**0.288**	**0.442**
VAR	0.350	0.279	0.301	0.249	0.381	0.516	0.450	0.933
GFT	–	–	1.237	–	–	–	–	–
Lu et al. (2019)	–	–	–	–	–	–	–	–
naive	0.265	0.250	**0.284**	0.244	0.302	0.360	0.347	0.513
Correlation								
ARGOX	**0.920**	**0.816**	0.708	**0.720**	**0.916**	**0.945**	**0.920**	**0.880**
VAR	0.825	0.787	**0.775**	0.677	0.809	0.815	0.867	0.563
GFT	–	–	0.605	–	–	–	–	–
Lu et al. (2019)	–	–	–	–	–	–	–	–
naive	0.896	0.812	0.745	0.702	0.875	0.881	0.895	0.811

Table S19. Comparison of different methods for state-level %ILI estimation in Iowa (IA). The MSE, MAE, and correlation are reported. The method with the best performance is highlighted in boldface for each metric in each period.

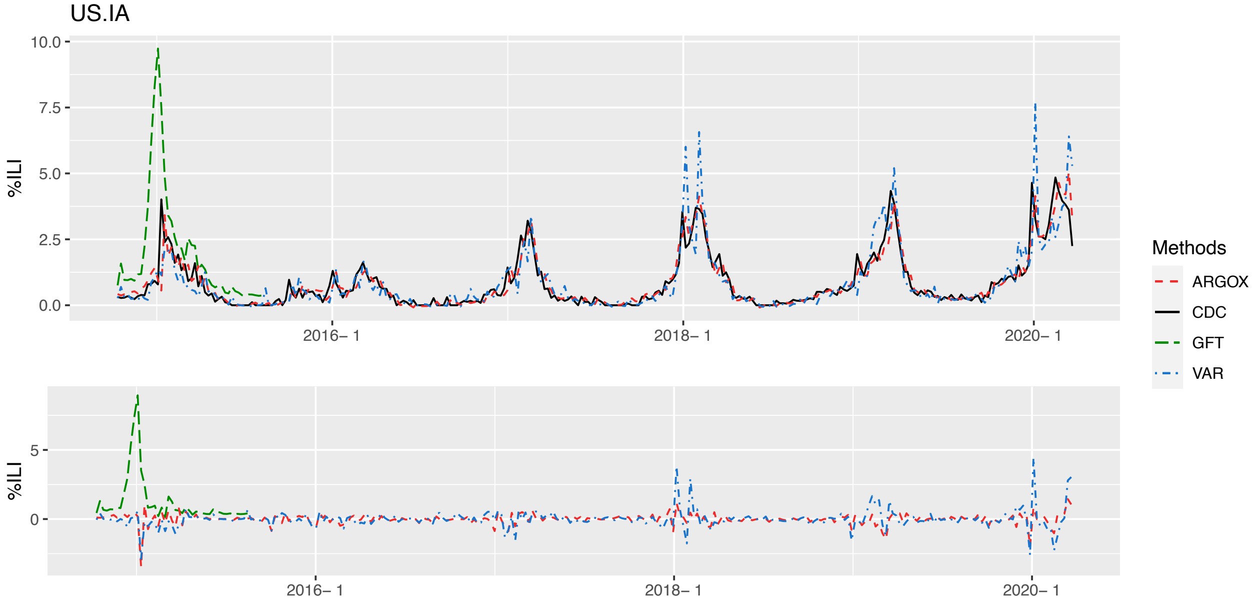

Figure S15. Plots of the %ILI estimates (top) and the estimation errors (bottom) for Iowa (IA).

	Whole period '14-'20	Overall '14-'17	'14-'15	'15-'16	'16-'17	'17-'18	'18-'19	'19-'20
MSE								
ARGOX	**0.269**	**0.326**	**0.450**	**0.069**	0.640	**0.260**	**0.230**	**0.463**
VAR	1.489	0.909	1.504	0.616	1.045	2.389	0.866	7.667
GFT	–	–	1.929	–	–	–	–	–
Lu et al. (2019)	–	0.328	0.607	0.118	**0.591**	–	–	–
naive	0.461	0.492	0.719	0.089	0.942	1.013	0.307	0.642
MAE								
ARGOX	**0.328**	**0.334**	**0.389**	0.224	**0.527**	**0.394**	**0.309**	**0.496**
VAR	0.664	0.562	0.733	0.530	0.675	1.038	0.692	1.673
GFT	–	–	0.983	–	–	–	–	–
Lu et al. (2019)	–	–	–	–	–	–	–	–
naive	0.405	0.397	0.481	**0.222**	0.674	0.709	0.381	0.586
Correlation								
ARGOX	**0.980**	**0.967**	**0.969**	**0.901**	0.953	**0.992**	**0.955**	**0.967**
VAR	0.919	0.918	0.919	0.697	0.919	0.954	0.871	0.699
GFT	–	–	0.962	–	–	–	–	–
Lu et al. (2019)	–	0.965	0.960	0.860	**0.961**	–	–	–
naive	0.965	0.948	0.945	0.874	0.929	0.965	0.937	0.959

Table S20. Comparison of different methods for state-level %ILI estimation in Kansas (KS). The MSE, MAE, and correlation are reported. The method with the best performance is highlighted in boldface for each metric in each period.

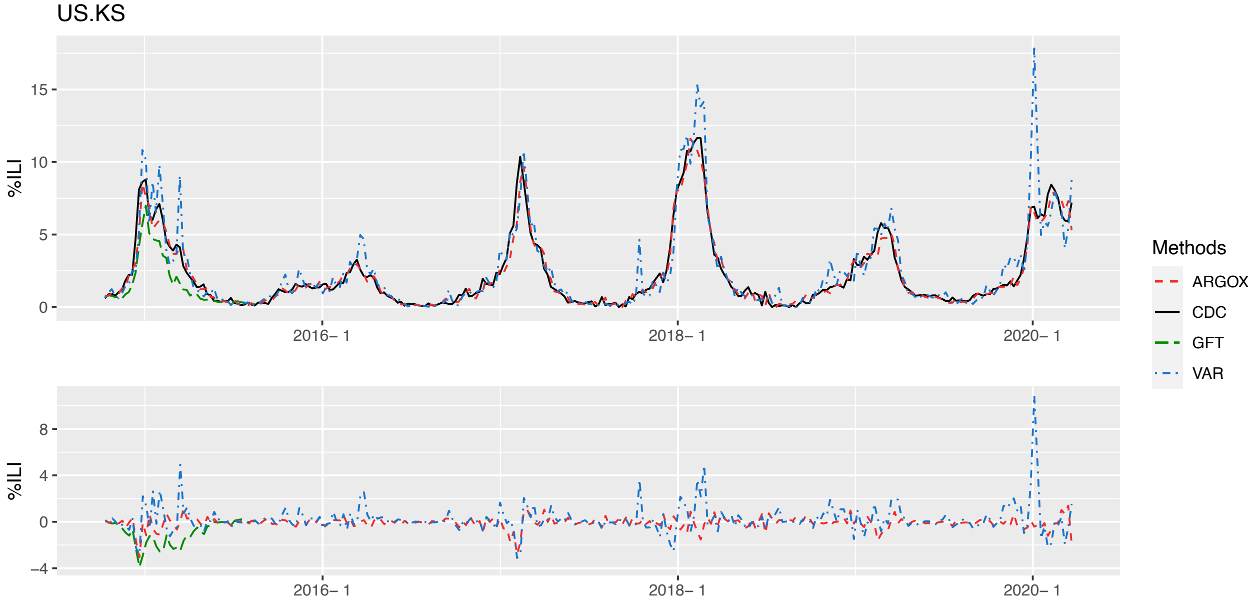

Figure S16. Plots of the %ILI estimates (top) and the estimation errors (bottom) for Kansas (KS).

	Whole period ’14-’20	Overall ’14-’17	’14-’15	’15-’16	’16-’17	’17-’18	’18-’19	’19-’20
MSE								
ARGOX	**0.467**	**0.310**	**0.071**	**0.058**	**1.049**	**0.871**	**0.785**	**1.342**
VAR	3.082	0.823	0.508	0.076	2.562	9.726	3.600	13.010
GFT	–	–	7.601	–	–	–	–	–
Lu et al. (2019)	–	0.415	0.106	0.076	1.433	–	–	–
naive	0.724	0.447	0.137	0.079	1.494	1.641	1.078	2.174
MAE								
ARGOX	**0.385**	**0.303**	**0.158**	**0.162**	**0.788**	**0.612**	**0.548**	**0.810**
VAR	0.777	0.408	0.288	0.194	1.014	1.715	1.293	2.222
GFT	–	–	1.951	–	–	–	–	–
Lu et al. (2019)	–	–	–	–	–	–	–	–
naive	0.466	0.351	0.197	0.190	0.913	0.851	0.680	1.072
Correlation								
ARGOX	**0.971**	**0.957**	0.954	**0.934**	**0.931**	**0.971**	**0.952**	**0.930**
VAR	0.879	0.876	0.905	0.922	0.825	0.899	0.857	0.617
GFT	–	–	**0.964**	–	–	–	–	–
Lu et al. (2019)	–	0.941	0.940	0.923	0.907	–	–	–
naive	0.955	0.936	0.910	0.908	0.898	0.943	0.934	0.891

Table S21. Comparison of different methods for state-level %ILI estimation in Kentucky (KY). The MSE, MAE, and correlation are reported. The method with the best performance is highlighted in boldface for each metric in each period.

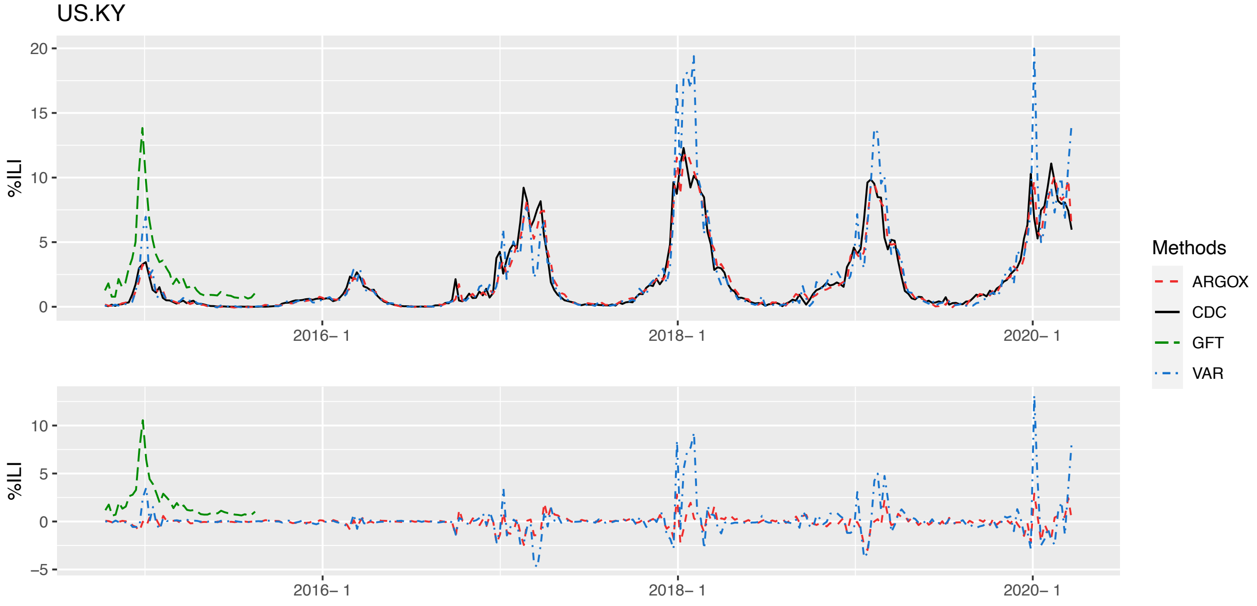

Figure S17. Plots of the %ILI estimates (top) and the estimation errors (bottom) for Kentucky (KY).

	Whole period '14-'20	Overall '14-'17	'14-'15	'15-'16	'16-'17	'17-'18	'18-'19	'19-'20
MSE								
ARGOX	**0.281**	**0.084**	**0.162**	**0.049**	**0.064**	**0.403**	0.770	**1.135**
VAR	0.554	0.310	0.654	0.194	0.115	0.574	**0.758**	2.684
GFT	–	–	0.917	–	–	–	–	–
Lu et al. (2019)	–	0.149	0.281	0.132	0.154	–	–	–
naive	0.429	0.144	0.287	0.062	0.126	0.817	0.963	1.667
MAE								
ARGOX	**0.306**	**0.191**	**0.258**	**0.177**	**0.189**	**0.444**	**0.560**	**0.792**
VAR	0.483	0.366	0.541	0.336	0.260	0.562	0.622	1.375
GFT	–	–	0.676	–	–	–	–	–
Lu et al. (2019)	–	–	–	–	–	–	–	–
naive	0.392	0.249	0.353	0.195	0.272	0.591	0.705	1.033
Correlation								
ARGOX	**0.979**	**0.977**	0.975	**0.921**	**0.974**	**0.979**	0.948	**0.884**
VAR	0.960	0.942	0.947	0.711	0.951	0.972	**0.959**	0.795
GFT	–	–	**0.984**	–	–	–	–	–
Lu et al. (2019)	–	0.963	0.955	0.796	0.958	–	–	–
naive	0.968	0.960	0.954	0.876	0.948	0.954	0.927	0.833

Table S22. Comparison of different methods for state-level %ILI estimation in Louisiana (LA). The MSE, MAE, and correlation are reported. The method with the best performance is highlighted in boldface for each metric in each period.

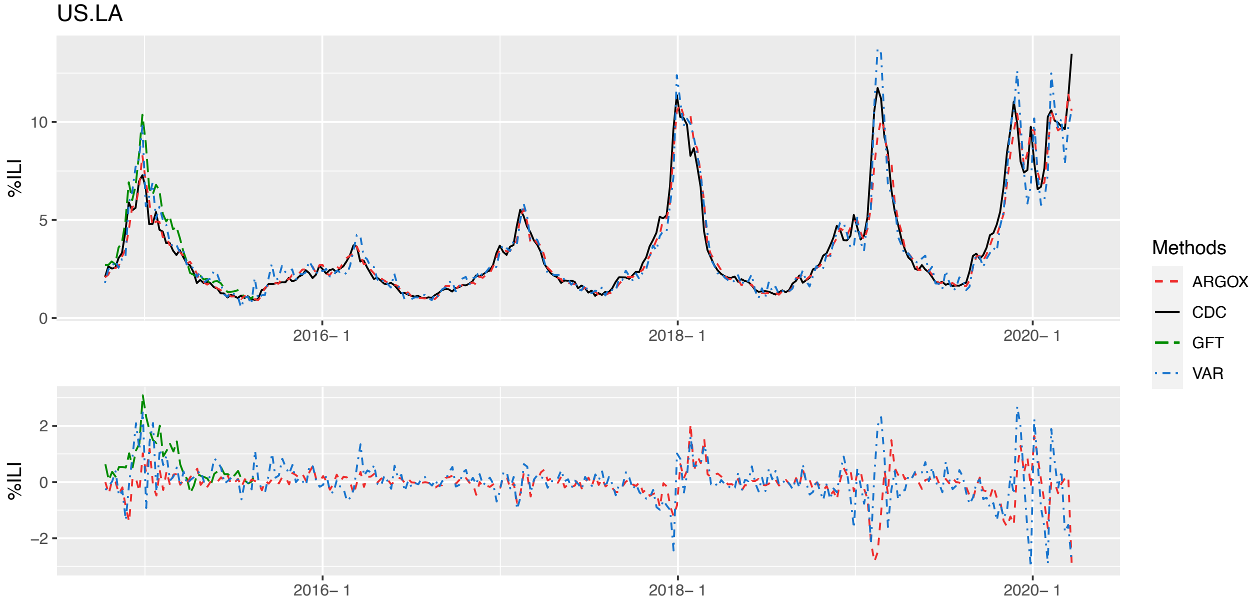

Figure S18. Plots of the %ILI estimates (top) and the estimation errors (bottom) for Louisiana (LA).

	Whole period '14-'20	Overall '14-'17	'14-'15	'15-'16	'16-'17	'17-'18	'18-'19	'19-'20
MSE								
ARGOX	**0.087**	0.086	**0.097**	0.109	0.078	**0.062**	0.090	**0.293**
VAR	0.176	0.172	0.230	0.159	0.132	0.139	0.317	0.401
GFT	–	–	0.497	–	–	–	–	–
Lu et al. (2019)	–	**0.073**	0.102	**0.094**	**0.061**	–	–	–
naive	0.097	0.105	0.118	0.141	0.088	0.062	**0.088**	0.303
MAE								
ARGOX	**0.208**	**0.225**	**0.237**	**0.264**	**0.221**	**0.192**	0.247	**0.331**
VAR	0.313	0.318	0.368	0.301	0.277	0.303	0.460	0.520
GFT	–	–	0.448	–	–	–	–	–
Lu et al. (2019)	–	–	–	–	–	–	–	–
naive	0.222	0.247	0.264	0.290	0.229	0.204	**0.232**	0.350
Correlation								
ARGOX	**0.957**	0.853	0.901	0.310	0.794	**0.806**	**0.943**	0.964
VAR	0.912	0.716	0.783	0.114	0.628	0.626	0.823	0.952
GFT	–	–	**0.914**	–	–	–	–	–
Lu et al. (2019)	–	**0.873**	0.897	**0.375**	**0.854**	–	–	–
naive	0.951	0.831	0.885	0.257	0.789	0.780	0.940	**0.964**

Table S23. Comparison of different methods for state-level %ILI estimation in Maine (ME). The MSE, MAE, and correlation are reported. The method with the best performance is highlighted in boldface for each metric in each period.

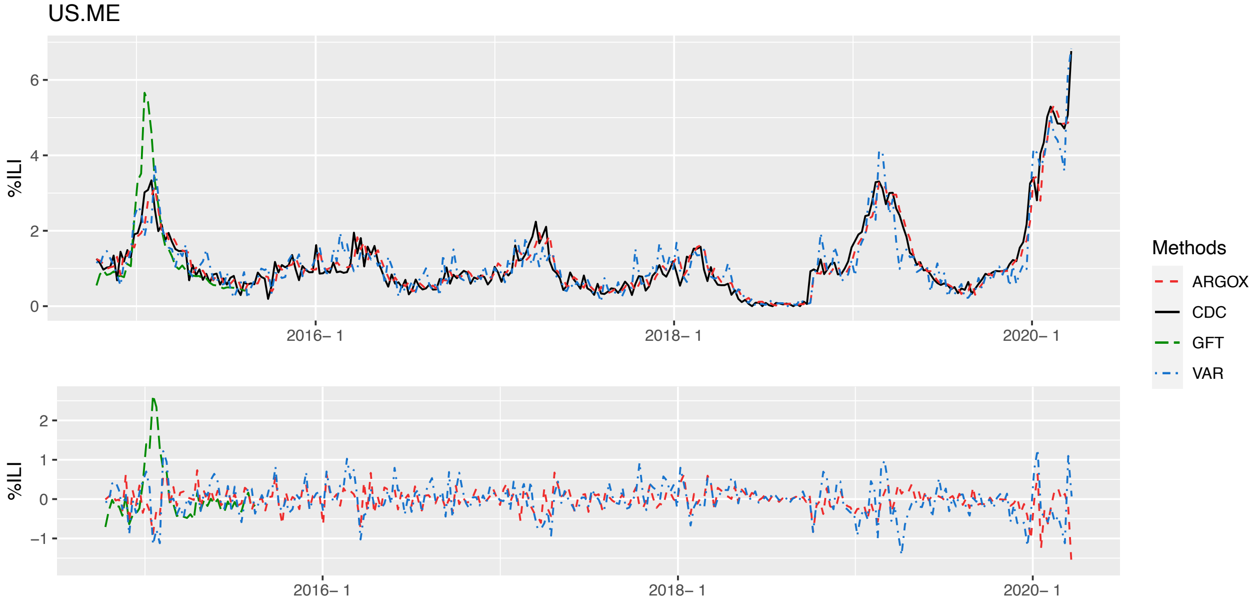

Figure S19. Plots of the %ILI estimates (top) and the estimation errors (bottom) for Maine (ME).

	Whole period '14-'20	Overall '14-'17	'14-'15	'15-'16	'16-'17	'17-'18	'18-'19	'19-'20
MSE								
ARGOX	**0.267**	**0.309**	**0.356**	**0.336**	**0.361**	**0.170**	**0.270**	**0.574**
VAR	0.831	0.846	1.053	0.645	0.846	0.463	0.620	2.937
GFT	–	–	1.504	–	–	–	–	–
Lu et al. (2019)	–	0.347	0.365	0.347	0.441	–	–	–
naive	0.376	0.433	0.507	0.432	0.520	0.376	0.389	0.678
MAE								
ARGOX	**0.374**	**0.413**	**0.396**	**0.465**	**0.487**	**0.331**	**0.409**	**0.517**
VAR	0.595	0.639	0.640	0.597	0.661	0.503	0.567	1.131
GFT	–	–	0.968	–	–	–	–	–
Lu et al. (2019)	–	–	–	–	–	–	–	–
naive	0.423	0.452	0.456	0.494	0.510	0.441	0.496	0.594
Correlation								
ARGOX	**0.940**	**0.846**	0.804	**0.732**	**0.918**	**0.968**	**0.911**	**0.947**
VAR	0.837	0.653	0.741	0.585	0.788	0.905	0.788	0.819
GFT	–	–	0.816	–	–	–	–	–
Lu et al. (2019)	–	0.824	**0.842**	0.718	0.885	–	–	–
naive	0.916	0.800	0.729	0.695	0.872	0.925	0.876	0.937

Table S24. Comparison of different methods for state-level %ILI estimation in Maryland (MD). The MSE, MAE, and correlation are reported. The method with the best performance is highlighted in boldface for each metric in each period.

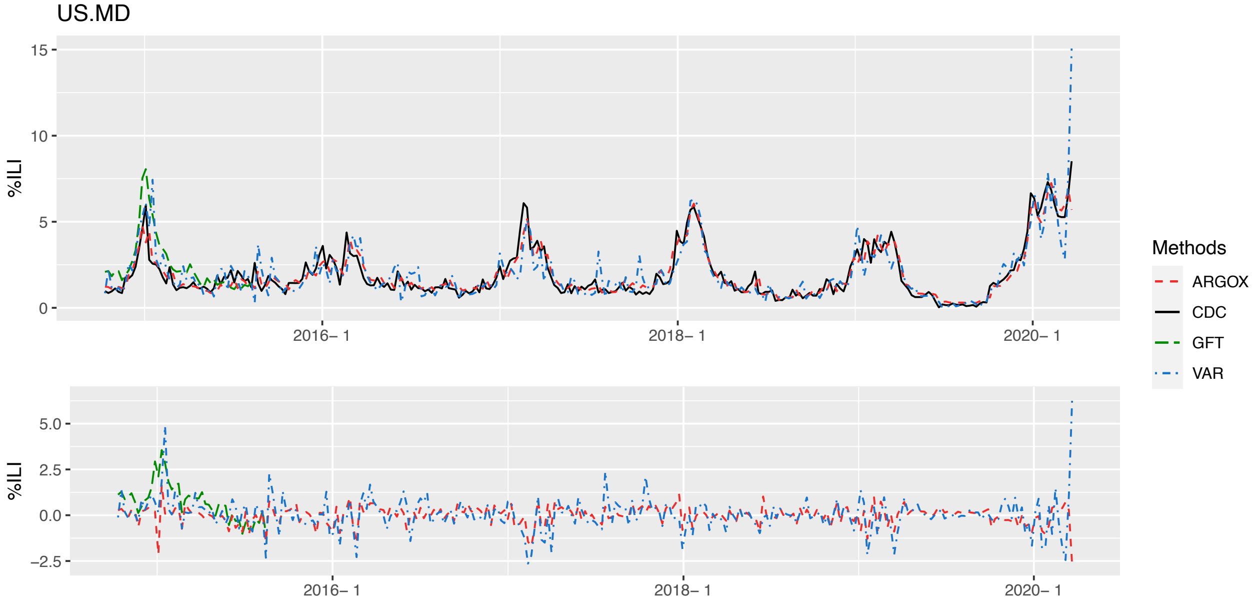

Figure S20. Plots of the %ILI estimates (top) and the estimation errors (bottom) for Maryland (MD).

	Whole period '14-'20	Overall '14-'17	'14-'15	'15-'16	'16-'17	'17-'18	'18-'19	'19-'20
MSE								
ARGOX	**0.080**	0.040	0.059	0.042	0.032	**0.125**	**0.043**	**0.469**
VAR	0.155	0.101	0.133	0.076	0.137	0.315	0.160	0.510
GFT	–	–	0.112	–	–	–	–	–
Lu et al. (2019)	–	**0.027**	**0.038**	**0.034**	**0.019**	–	–	–
naive	0.126	0.070	0.100	0.066	0.073	0.284	0.105	0.528
MAE								
ARGOX	**0.161**	**0.139**	**0.146**	**0.160**	**0.145**	**0.243**	**0.152**	**0.397**
VAR	0.266	0.234	0.263	0.219	0.283	0.393	0.293	0.540
GFT	–	–	0.263	–	–	–	–	–
Lu et al. (2019)	–	–	–	–	–	–	–	–
naive	0.217	0.181	0.214	0.181	0.203	0.358	0.231	0.530
Correlation								
ARGOX	**0.970**	0.949	0.936	0.912	0.959	**0.968**	**0.966**	**0.930**
VAR	0.938	0.874	0.869	0.845	0.818	0.927	0.884	0.928
GFT	–	–	0.947	–	–	–	–	–
Lu et al. (2019)	–	**0.967**	**0.966**	**0.929**	**0.976**	–	–	–
naive	0.950	0.912	0.891	0.870	0.908	0.921	0.919	0.923

Table S25. Comparison of different methods for state-level %ILI estimation in Massachusetts (MA). The MSE, MAE, and correlation are reported. The method with the best performance is highlighted in boldface for each metric in each period.

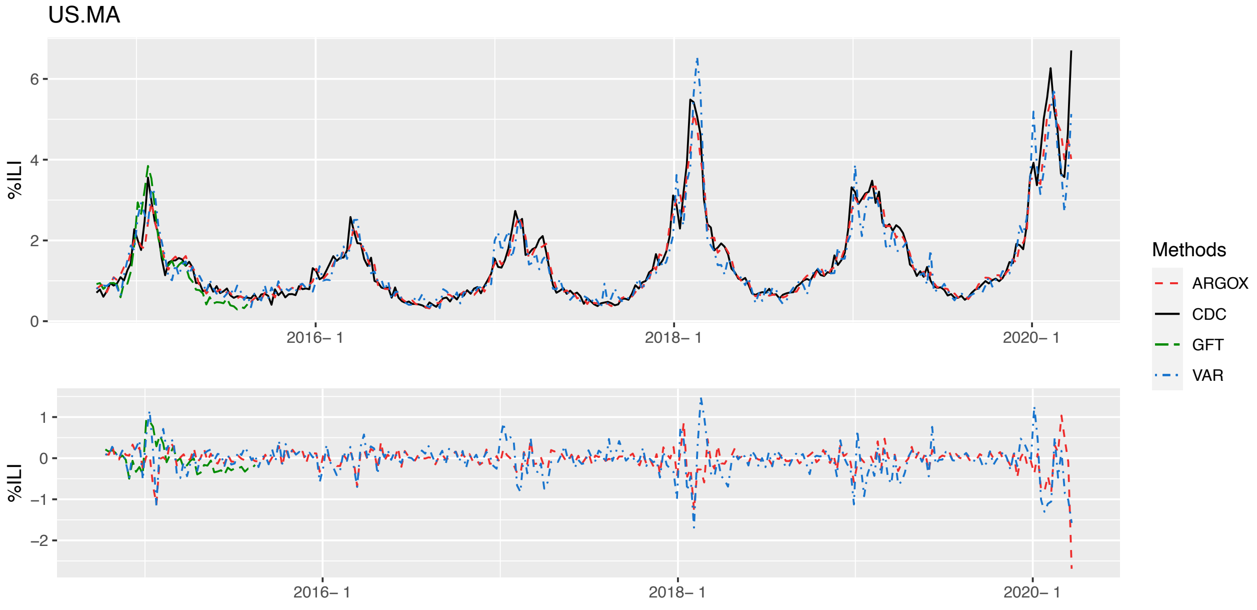

Figure S21. Plots of the %ILI estimates (top) and the estimation errors (bottom) for Massachusetts (MA).

	Whole period '14-'20	Overall '14-'17	'14-'15	'15-'16	'16-'17	'17-'18	'18-'19	'19-'20
MSE								
ARGOX	**0.073**	**0.077**	**0.131**	**0.032**	**0.093**	**0.100**	**0.082**	**0.119**
VAR	0.177	0.168	0.174	0.174	0.249	0.318	0.181	0.317
GFT	–	–	1.313	–	–	–	–	–
Lu et al. (2019)	–	0.132	0.246	0.081	0.174	–	–	–
naive	0.130	0.146	0.260	0.066	0.169	0.232	0.108	0.177
MAE								
ARGOX	**0.187**	**0.183**	**0.237**	**0.134**	**0.221**	**0.256**	**0.222**	**0.253**
VAR	0.292	0.293	0.284	0.309	0.403	0.413	0.314	0.380
GFT	–	–	0.957	–	–	–	–	–
Lu et al. (2019)	–	–	–	–	–	–	–	–
naive	0.236	0.227	0.284	0.184	0.299	0.392	0.255	0.308
Correlation								
ARGOX	**0.969**	**0.962**	0.935	**0.963**	**0.955**	**0.977**	**0.873**	**0.949**
VAR	0.934	0.928	0.938	0.905	0.874	0.940	0.795	0.871
GFT	–	–	**0.979**	–	–	–	–	–
Lu et al. (2019)	–	0.936	0.919	0.908	0.928	–	–	–
naive	0.945	0.928	0.868	0.917	0.917	0.939	0.837	0.930

Table S26. Comparison of different methods for state-level %ILI estimation in Michigan (MI). The MSE, MAE, and correlation are reported. The method with the best performance is highlighted in boldface for each metric in each period.

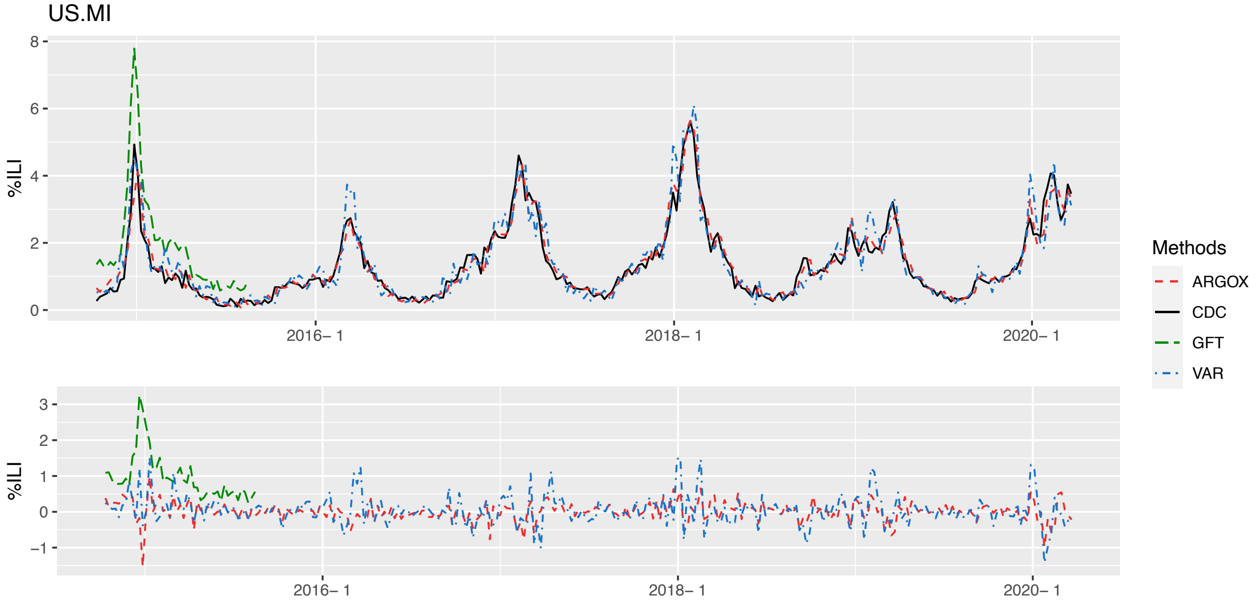

Figure S22. Plots of the %ILI estimates (top) and the estimation errors (bottom) for Michigan (MI).

	Whole period '14-'20	Overall '14-'17	'14-'15	'15-'16	'16-'17	'17-'18	'18-'19	'19-'20
MSE								
ARGOX	**0.309**	**0.251**	**0.388**	**0.283**	**0.174**	**0.148**	**0.266**	**1.447**
VAR	0.953	1.049	1.552	0.634	1.414	0.387	1.047	2.487
GFT	–	–	0.544	–	–	–	–	–
Lu et al. (2019)	–	0.287	0.472	0.370	0.215	–	–	–
naive	0.499	0.443	0.722	0.510	0.277	0.247	0.289	2.395
MAE								
ARGOX	**0.371**	**0.374**	**0.436**	**0.460**	**0.333**	**0.320**	**0.364**	**0.773**
VAR	0.644	0.640	0.713	0.608	0.779	0.475	0.779	1.143
GFT	–	–	0.527	–	–	–	–	–
Lu et al. (2019)	–	–	–	–	–	–	–	–
naive	0.441	0.440	0.535	0.544	0.378	0.388	0.402	0.965
Correlation								
ARGOX	**0.929**	**0.922**	0.923	**0.770**	**0.935**	**0.978**	**0.825**	**0.726**
VAR	0.815	0.779	0.839	0.576	0.536	0.948	0.662	0.602
GFT	–	–	**0.931**	–	–	–	–	–
Lu et al. (2019)	–	0.907	0.910	0.703	0.915	–	–	–
naive	0.888	0.865	0.855	0.629	0.888	0.960	0.807	0.575

Table S27. Comparison of different methods for state-level %ILI estimation in Minnesota (MN). The MSE, MAE, and correlation are reported. The method with the best performance is highlighted in boldface for each metric in each period.

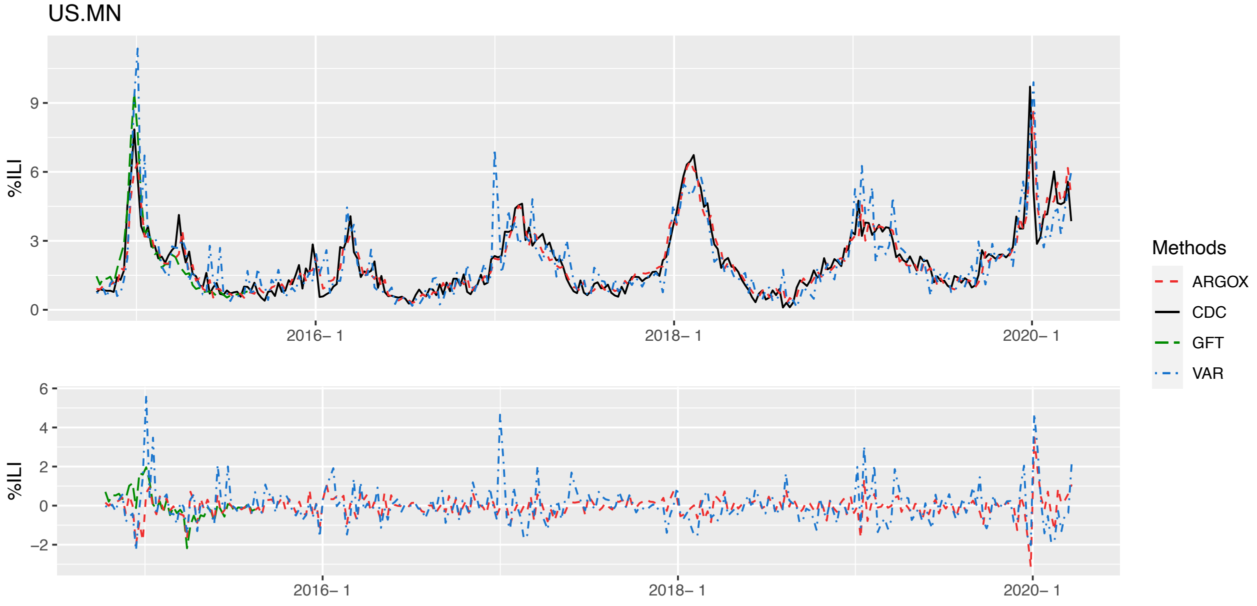

Figure S23. Plots of the %ILI estimates (top) and the estimation errors (bottom) for Minnesota (MN).

	Whole period '14-'20	Overall '14-'17	'14-'15	'15-'16	'16-'17	'17-'18	'18-'19	'19-'20
MSE								
ARGOX	**0.334**	**0.224**	**0.381**	**0.092**	**0.241**	**0.961**	**0.358**	**0.640**
VAR	0.821	0.462	0.663	0.439	0.428	2.412	0.989	1.619
GFT	–	–	1.936	–	–	–	–	–
Lu et al. (2019)	–	–	–	–	–	–	–	–
naive	0.622	0.476	0.931	0.200	0.422	1.690	0.706	1.043
MAE								
ARGOX	**0.384**	**0.335**	**0.413**	**0.261**	**0.392**	**0.677**	**0.415**	**0.665**
VAR	0.614	0.510	0.571	0.533	0.531	1.032	0.724	0.939
GFT	–	–	1.220	–	–	–	–	–
Lu et al. (2019)	–	–	–	–	–	–	–	–
naive	0.508	0.450	0.606	0.344	0.519	0.882	0.601	0.851
Correlation								
ARGOX	**0.967**	**0.965**	**0.969**	**0.921**	**0.932**	**0.963**	**0.962**	**0.894**
VAR	0.932	0.932	0.947	0.731	0.879	0.931	0.925	0.778
GFT	–	–	0.960	–	–	–	–	–
Lu et al. (2019)	–	–	–	–	–	–	–	–
naive	0.940	0.926	0.923	0.835	0.883	0.930	0.912	0.822

Table S28. Comparison of different methods for state-level %ILI estimation in Mississippi (MS). The MSE, MAE, and correlation are reported. The method with the best performance is highlighted in boldface for each metric in each period.

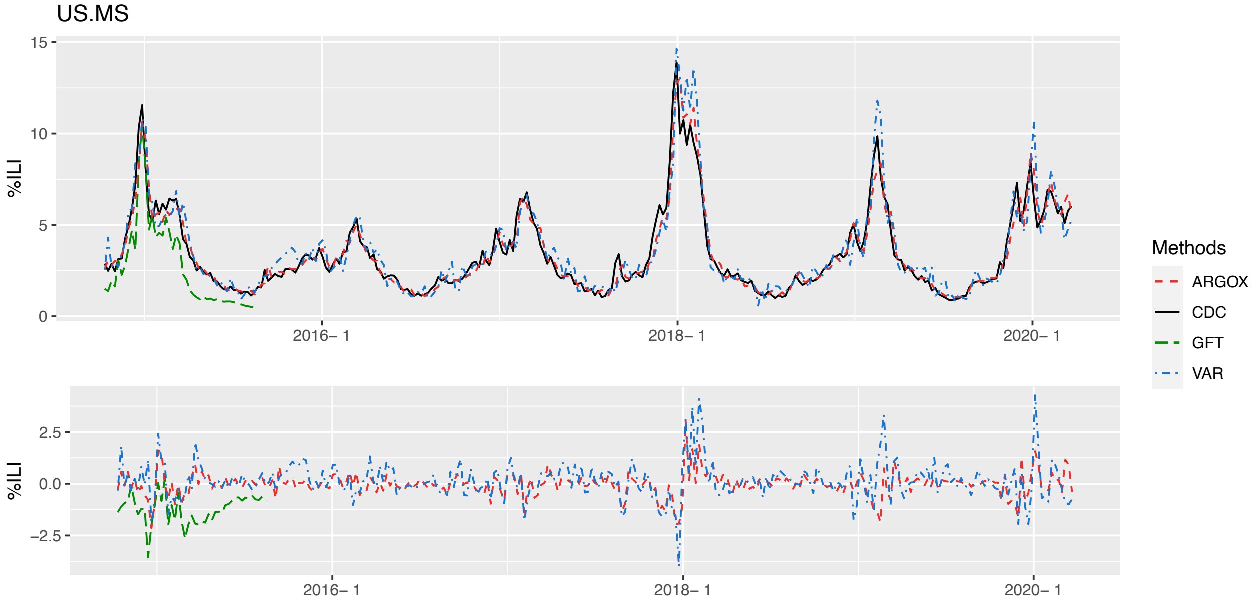

Figure S24. Plots of the %ILI estimates (top) and the estimation errors (bottom) for Mississippi (MS).

	Whole period '14-'20	Overall '14-'17	'14-'15	'15-'16	'16-'17	'17-'18	'18-'19	'19-'20
MSE								
ARGOX	**0.442**	**0.241**	**0.336**	**0.139**	**0.353**	**0.817**	**0.654**	**1.709**
VAR	2.090	0.934	0.843	0.969	1.633	6.039	1.068	9.347
GFT	–	–	0.482	–	–	–	–	–
Lu et al. (2019)	–	–	–	–	–	–	–	–
naive	0.767	0.412	0.741	0.155	0.478	1.818	1.017	2.692
MAE								
ARGOX	**0.392**	**0.319**	**0.358**	0.314	**0.379**	**0.571**	**0.559**	**0.918**
VAR	0.712	0.611	0.637	0.639	0.795	1.068	0.691	2.100
GFT	–	–	0.570	–	–	–	–	–
Lu et al. (2019)	–	–	–	–	–	–	–	–
naive	0.499	0.392	0.486	**0.311**	0.466	0.883	0.718	1.114
Correlation								
ARGOX	**0.963**	**0.951**	0.956	**0.775**	**0.939**	**0.972**	**0.936**	**0.929**
VAR	0.866	0.852	0.883	0.626	0.844	0.897	0.901	0.658
GFT	–	–	**0.959**	–	–	–	–	–
Lu et al. (2019)	–	–	–	–	–	–	–	–
naive	0.935	0.916	0.898	0.761	0.919	0.938	0.890	0.895

Table S29. Comparison of different methods for state-level %ILI estimation in Missouri (MO). The MSE, MAE, and correlation are reported. The method with the best performance is highlighted in boldface for each metric in each period.

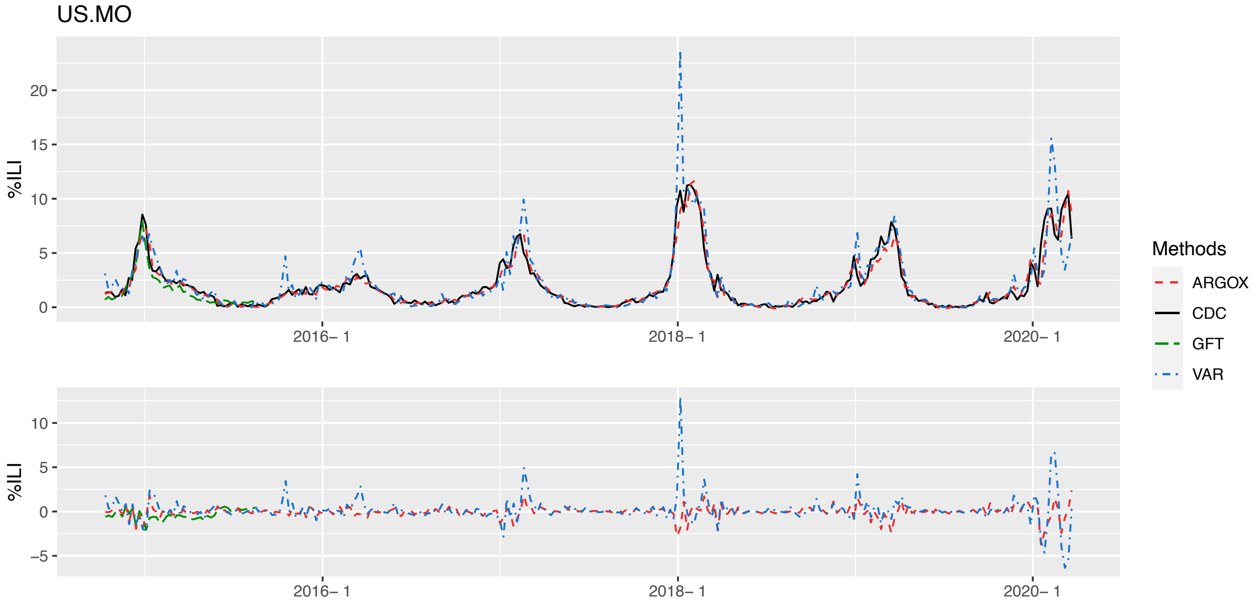

Figure S25. Plots of the %ILI estimates (top) and the estimation errors (bottom) for Missouri (MO).

	Whole period '14-'20	Overall '14-'17	'14-'15	'15-'16	'16-'17	'17-'18	'18-'19	'19-'20
MSE								
ARGOX	**0.055**	**0.039**	**0.061**	**0.055**	**0.020**	**0.022**	0.194	**0.121**
VAR	0.198	0.157	0.275	0.080	0.136	0.037	0.351	0.885
GFT	–	–	1.095	–	–	–	–	–
Lu et al. (2019)	–	–	–	–	–	–	–	–
naive	0.061	0.049	0.079	0.067	0.024	0.029	**0.184**	0.142
MAE								
ARGOX	**0.143**	**0.131**	0.181	**0.164**	**0.100**	**0.110**	0.322	**0.254**
VAR	0.248	0.232	0.330	0.216	0.212	0.140	0.449	0.598
GFT	–	–	0.870	–	–	–	–	–
Lu et al. (2019)	–	–	–	–	–	–	–	–
naive	0.144	0.132	**0.179**	0.169	0.112	0.120	**0.320**	0.271
Correlation								
ARGOX	**0.967**	**0.854**	0.893	**0.419**	**0.580**	**0.779**	**0.919**	**0.972**
VAR	0.877	0.552	0.585	0.367	0.398	0.668	0.897	0.802
GFT	–	–	**0.939**	–	–	–	–	–
Lu et al. (2019)	–	–	–	–	–	–	–	–
naive	0.963	0.821	0.865	0.341	0.499	0.736	0.916	0.969

Table S30. Comparison of different methods for state-level %ILI estimation in Montana (MT). The MSE, MAE, and correlation are reported. The method with the best performance is highlighted in boldface for each metric in each period.

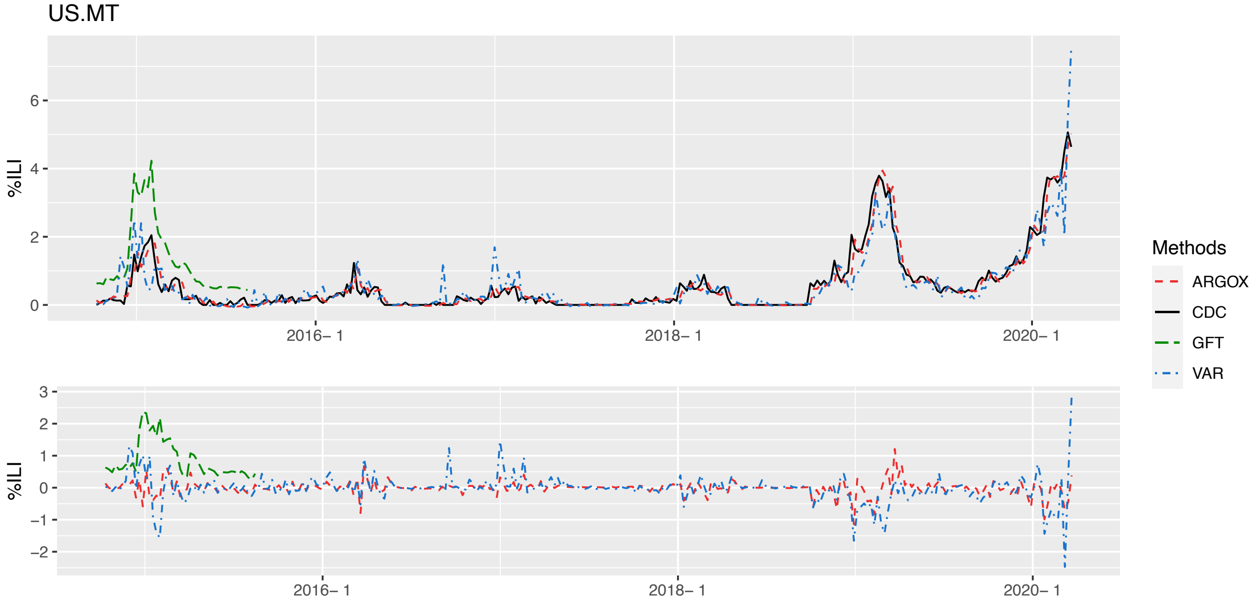

Figure S26. Plots of the %ILI estimates (top) and the estimation errors (bottom) for Montana (MT).

	Whole period '14-'20	Overall '14-'17	'14-'15	'15-'16	'16-'17	'17-'18	'18-'19	'19-'20
MSE								
ARGOX	**0.380**	**0.231**	**0.470**	0.086	**0.195**	**0.684**	**0.755**	**0.840**
VAR	2.520	1.172	3.148	0.093	0.353	2.474	4.081	13.168
GFT	–	–	0.678	–	–	–	–	–
Lu et al. (2019)	–	0.265	0.605	0.141	0.251	–	–	–
naive	0.497	0.303	0.636	**0.082**	0.272	0.822	0.944	1.341
MAE								
ARGOX	**0.392**	**0.287**	**0.389**	0.234	**0.303**	**0.617**	**0.574**	**0.704**
VAR	0.779	0.490	0.881	0.253	0.373	1.175	1.295	1.846
GFT	–	–	0.501	–	–	–	–	–
Lu et al. (2019)	–	–	–	–	–	–	–	–
naive	0.434	0.318	0.460	**0.211**	0.353	0.692	0.684	0.736
Correlation								
ARGOX	**0.950**	0.866	0.835	0.631	**0.897**	**0.925**	**0.918**	**0.900**
VAR	0.769	0.495	0.255	0.498	0.818	0.723	0.628	0.735
GFT	–	–	0.846	–	–	–	–	–
Lu et al. (2019)	–	**0.878**	**0.861**	**0.680**	0.863	–	–	–
naive	0.936	0.836	0.788	0.608	0.859	0.902	0.901	0.842

Table S31. Comparison of different methods for state-level %ILI estimation in Nebraska (NE). The MSE, MAE, and correlation are reported. The method with the best performance is highlighted in boldface for each metric in each period.

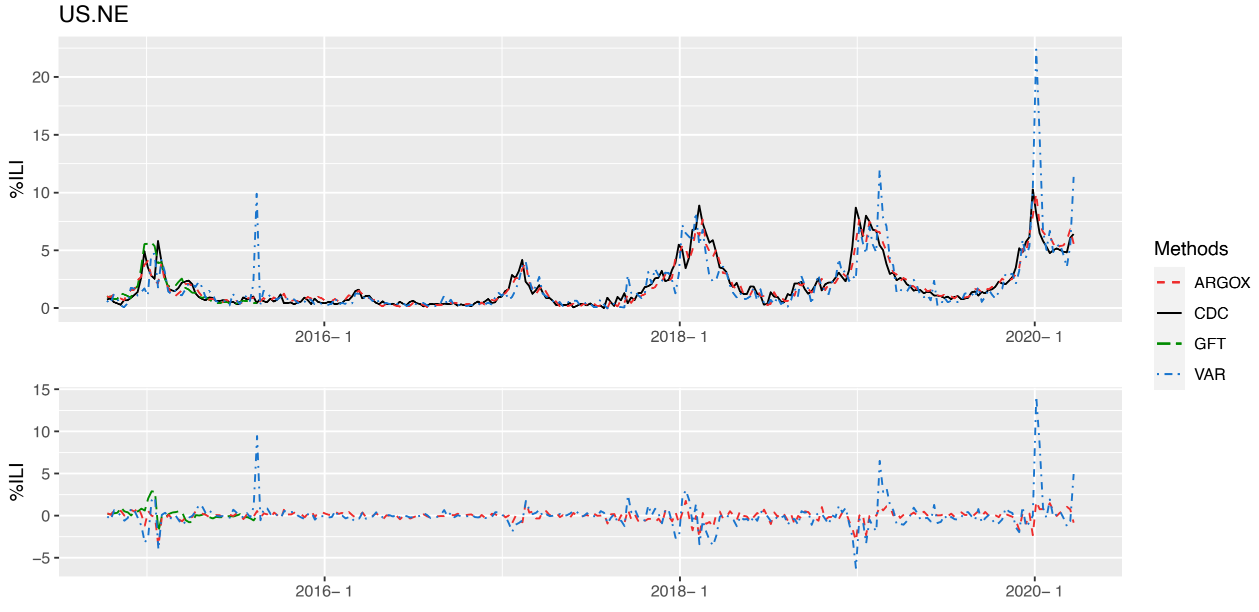

Figure S27. Plots of the %ILI estimates (top) and the estimation errors (bottom) for Nebraska (NE).

	Whole period '14-'20	Overall '14-'17	'14-'15	'15-'16	'16-'17	'17-'18	'18-'19	'19-'20
MSE								
ARGOX	**0.096**	**0.089**	**0.132**	**0.096**	0.066	**0.046**	**0.142**	0.322
VAR	0.232	0.163	0.185	0.199	0.187	0.395	0.342	0.687
GFT	–	–	2.565	–	–	–	–	–
Lu et al. (2019)	–	0.112	0.261	0.112	**0.053**	–	–	–
naive	0.115	0.109	0.179	0.131	0.060	0.122	0.184	**0.256**
MAE								
ARGOX	**0.208**	**0.210**	**0.257**	**0.224**	0.197	**0.170**	**0.231**	0.477
VAR	0.318	0.295	0.328	0.330	0.335	0.374	0.423	0.607
GFT	–	–	1.509	–	–	–	–	–
Lu et al. (2019)	–	–	–	–	–	–	–	–
naive	0.224	0.218	0.288	0.251	**0.185**	0.262	0.283	**0.378**
Correlation								
ARGOX	**0.940**	**0.932**	**0.948**	**0.901**	0.876	**0.977**	**0.920**	0.699
VAR	0.874	0.882	0.927	0.829	0.769	0.944	0.863	0.705
GFT	–	–	0.936	–	–	–	–	–
Lu et al. (2019)	–	0.915	0.896	0.881	**0.884**	–	–	–
naive	0.929	0.919	0.929	0.859	0.865	0.933	0.898	**0.766**

Table S32. Comparison of different methods for state-level %ILI estimation in Nevada (NV). The MSE, MAE, and correlation are reported. The method with the best performance is highlighted in boldface for each metric in each period.

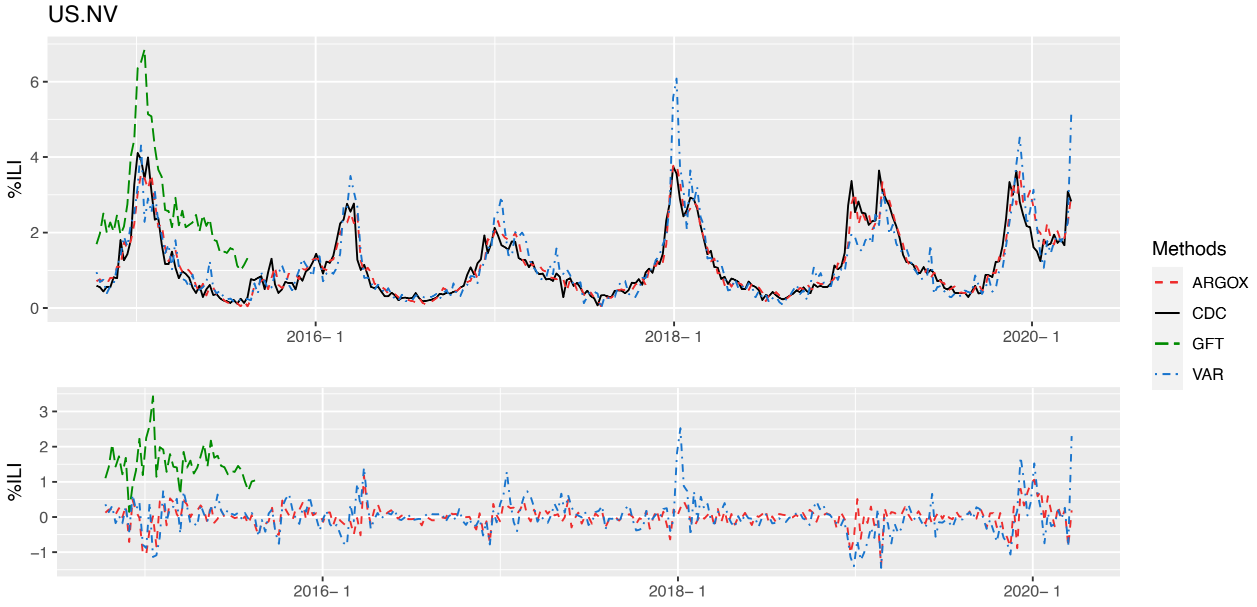

Figure S28. Plots of the %ILI estimates (top) and the estimation errors (bottom) for Nevada (NV).

	Whole period '14-'20	Overall '14-'17	'14-'15	'15-'16	'16-'17	'17-'18	'18-'19	'19-'20
MSE								
ARGOX	**0.175**	0.054	0.058	**0.021**	0.111	0.470	**0.334**	**0.476**
VAR	0.363	0.081	0.113	0.048	0.086	**0.447**	0.617	1.991
GFT	–	–	1.113	–	–	–	–	–
Lu et al. (2019)	–	**0.045**	0.061	0.030	**0.071**	–	–	–
naive	0.201	0.056	**0.052**	0.027	0.120	0.616	0.374	0.477
MAE								
ARGOX	**0.247**	**0.152**	0.150	**0.110**	0.232	**0.452**	**0.337**	**0.462**
VAR	0.357	0.198	0.226	0.168	**0.222**	0.466	0.539	0.989
GFT	–	–	0.844	–	–	–	–	–
Lu et al. (2019)	–	–	–	–	–	–	–	–
naive	0.266	0.153	**0.142**	0.116	0.242	0.464	0.384	0.529
Correlation								
ARGOX	**0.913**	0.846	0.887	**0.912**	0.672	0.916	**0.750**	0.909
VAR	0.817	0.776	0.766	0.778	0.783	**0.930**	0.554	0.572
GFT	–	–	**0.915**	–	–	–	–	–
Lu et al. (2019)	–	**0.877**	0.887	0.894	**0.813**	–	–	–
naive	0.904	0.854	0.902	0.883	0.692	0.887	0.743	**0.914**

Table S33. Comparison of different methods for state-level %ILI estimation in New Hampshire (NH). The MSE, MAE, and correlation are reported. The method with the best performance is highlighted in boldface for each metric in each period.

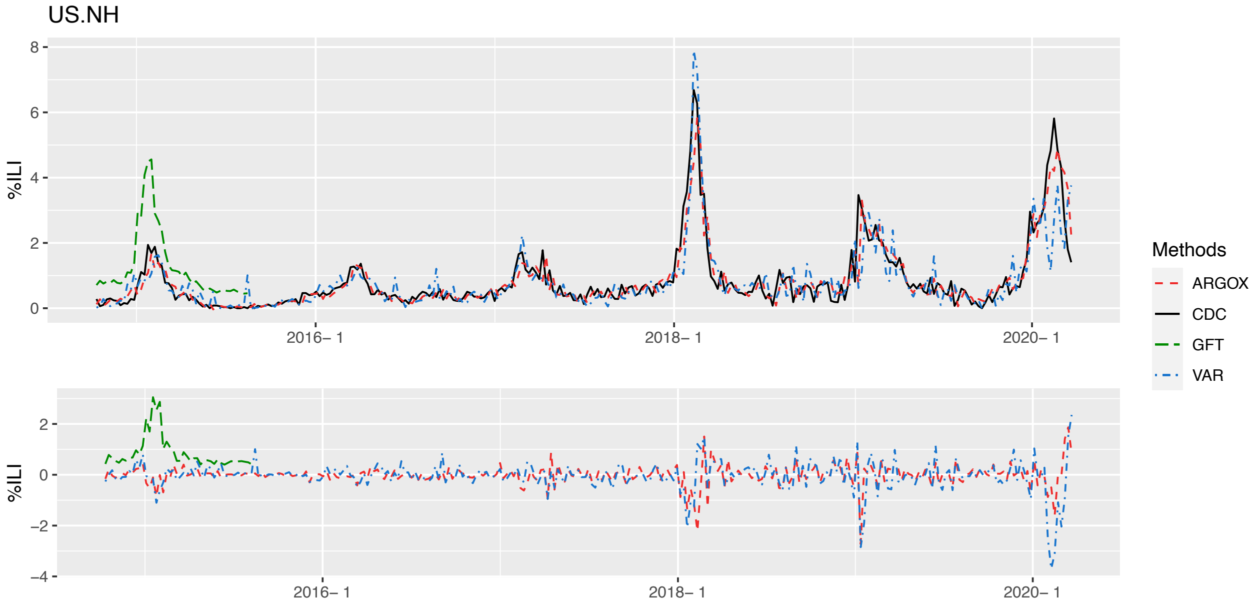

Figure S29. Plots of the %ILI estimates (top) and the estimation errors (bottom) for New Hampshire (NH).

	Whole period '14-'20	Overall '14-'17	'14-'15	'15-'16	'16-'17	'17-'18	'18-'19	'19-'20
MSE								
ARGOX	**0.301**	0.244	**0.218**	0.228	0.408	**0.427**	**0.041**	**1.491**
VAR	0.757	0.797	1.205	0.440	1.098	1.405	0.257	1.542
GFT	–	–	0.644	–	–	–	–	–
Lu et al. (2019)	–	**0.243**	0.250	**0.228**	0.466	–	–	–
naive	0.419	0.275	0.318	0.253	**0.367**	0.979	0.186	1.689
MAE								
ARGOX	**0.325**	**0.381**	**0.354**	**0.373**	0.519	**0.452**	**0.160**	**0.584**
VAR	0.562	0.621	0.775	0.462	0.836	0.727	0.350	0.811
GFT	–	–	0.672	–	–	–	–	–
Lu et al. (2019)	–	–	–	–	–	–	–	–
naive	0.387	0.384	0.412	0.376	**0.456**	0.594	0.303	0.797
Correlation								
ARGOX	**0.964**	0.944	**0.896**	**0.924**	**0.926**	**0.976**	**0.987**	**0.903**
VAR	0.907	0.826	0.638	0.835	0.766	0.911	0.926	0.888
GFT	–	–	0.896	–	–	–	–	–
Lu et al. (2019)	–	**0.944**	0.887	0.922	0.922	–	–	–
naive	0.948	0.938	0.851	0.909	0.917	0.930	0.940	0.894

Table S34. Comparison of different methods for state-level %ILI estimation in New Jersey (NJ). The MSE, MAE, and correlation are reported. The method with the best performance is highlighted in boldface for each metric in each period.

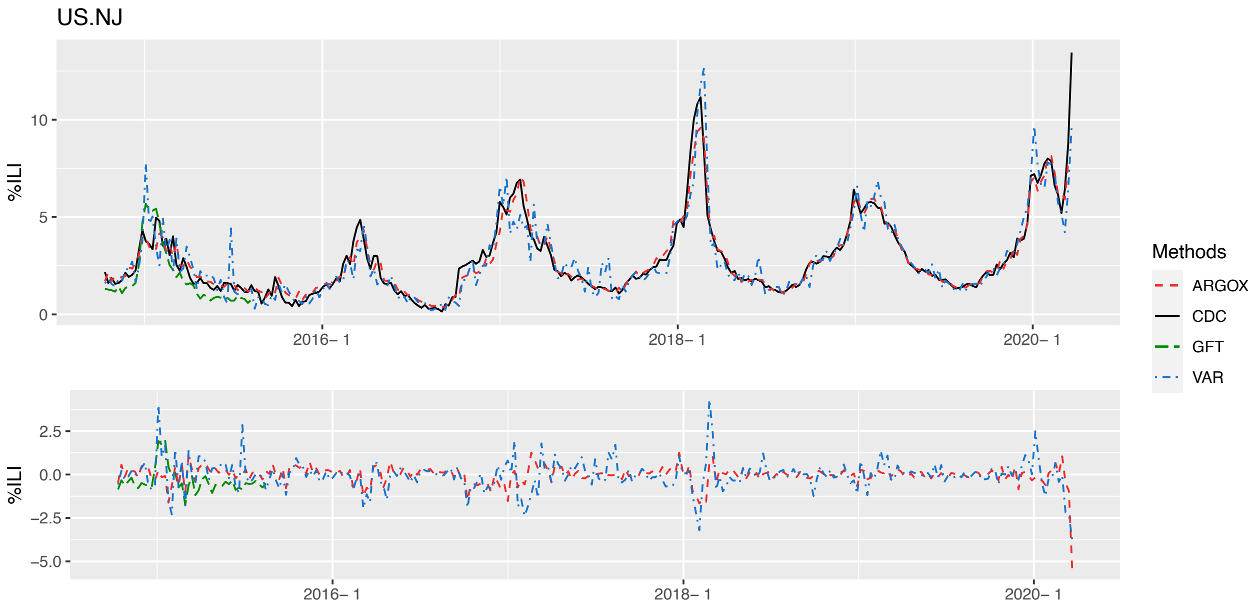

Figure S30. Plots of the %ILI estimates (top) and the estimation errors (bottom) for New Jersey (NJ).

	Whole period '14-'20	Overall '14-'17	'14-'15	'15-'16	'16-'17	'17-'18	'18-'19	'19-'20
MSE								
ARGOX	**0.263**	**0.146**	**0.193**	**0.180**	0.100	**0.487**	**0.315**	**1.031**
VAR	0.598	0.477	0.700	0.401	0.519	0.751	0.825	1.958
GFT	–	–	1.944	–	–	–	–	–
Lu et al. (2019)	–	0.164	0.293	0.213	**0.093**	–	–	–
naive	0.417	0.247	0.399	0.234	0.167	0.807	0.560	1.499
MAE								
ARGOX	**0.325**	**0.274**	**0.299**	**0.321**	**0.241**	**0.401**	**0.358**	**0.785**
VAR	0.478	0.454	0.475	0.487	0.516	0.538	0.611	0.928
GFT	–	–	1.222	–	–	–	–	–
Lu et al. (2019)	–	–	–	–	–	–	–	–
naive	0.389	0.329	0.368	0.368	0.310	0.580	0.501	0.806
Correlation								
ARGOX	**0.966**	**0.948**	0.945	**0.935**	0.962	**0.957**	**0.959**	**0.946**
VAR	0.926	0.871	0.895	0.843	0.845	0.942	0.888	0.903
GFT	–	–	**0.951**	–	–	–	–	–
Lu et al. (2019)	–	0.943	0.923	0.927	**0.966**	–	–	–
naive	0.946	0.912	0.881	0.916	0.935	0.926	0.924	0.925

Table S35. Comparison of different methods for state-level %ILI estimation in New Mexico (NM). The MSE, MAE, and correlation are reported. The method with the best performance is highlighted in boldface for each metric in each period.

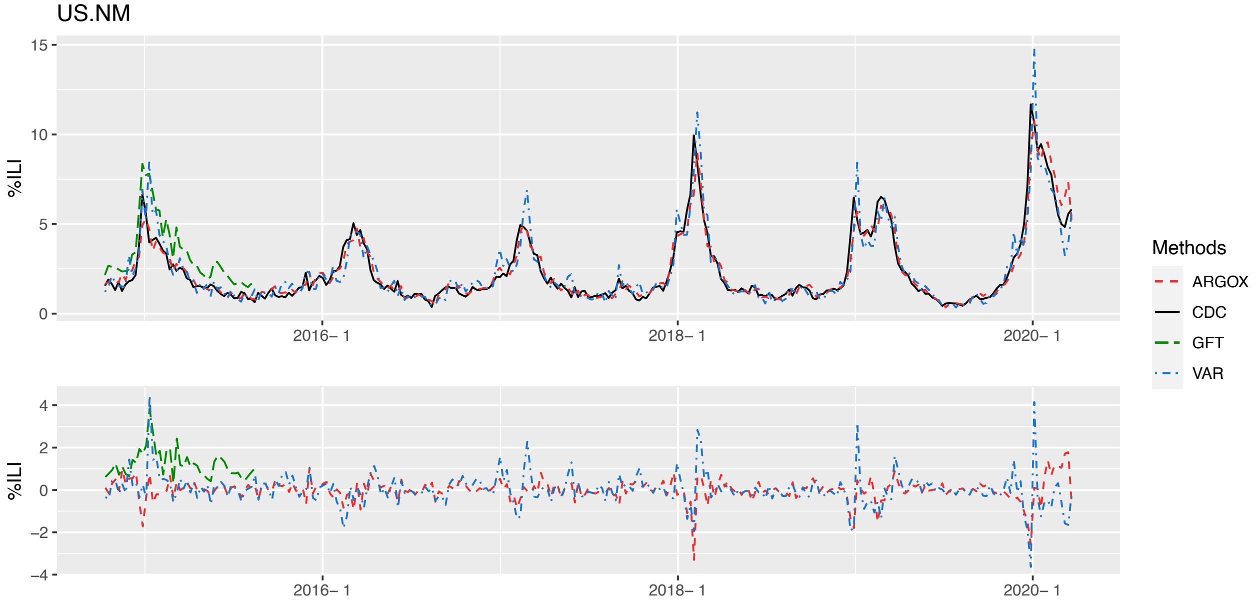

Figure S31. Plots of the %ILI estimates (top) and the estimation errors (bottom) for New Mexico (NM).

	Whole period '14-'20	Overall '14-'17	'14-'15	'15-'16	'16-'17	'17-'18	'18-'19	'19-'20
MSE								
ARGOX	**0.363**	**0.469**	0.425	**0.254**	**1.028**	**0.532**	**0.080**	**0.668**
VAR	1.863	2.812	0.792	0.522	9.831	1.794	0.431	2.434
GFT	–	–	0.686	–	–	–	–	–
Lu et al. (2019)	–	1.228	**0.349**	0.461	3.932	–	–	–
naive	0.501	0.577	0.631	0.336	1.124	0.956	0.143	1.026
MAE								
ARGOX	**0.395**	**0.482**	**0.480**	**0.393**	0.755	**0.524**	**0.212**	**0.556**
VAR	0.690	0.755	0.594	0.544	1.581	0.952	0.486	1.003
GFT	–	–	0.630	–	–	–	–	–
Lu et al. (2019)	–	–	–	–	–	–	–	–
naive	0.426	0.489	0.514	0.432	**0.732**	0.648	0.291	0.735
Correlation								
ARGOX	**0.959**	**0.939**	0.925	**0.837**	**0.925**	**0.970**	**0.965**	**0.939**
VAR	0.849	0.769	0.859	0.756	0.676	0.921	0.817	0.856
GFT	–	–	0.903	–	–	–	–	–
Lu et al. (2019)	–	0.856	**0.948**	0.751	0.782	–	–	–
naive	0.943	0.926	0.890	0.794	0.915	0.943	0.940	0.904

Table S36. Comparison of different methods for state-level %ILI estimation in New York (NY). The MSE, MAE, and correlation are reported. The method with the best performance is highlighted in boldface for each metric in each period.

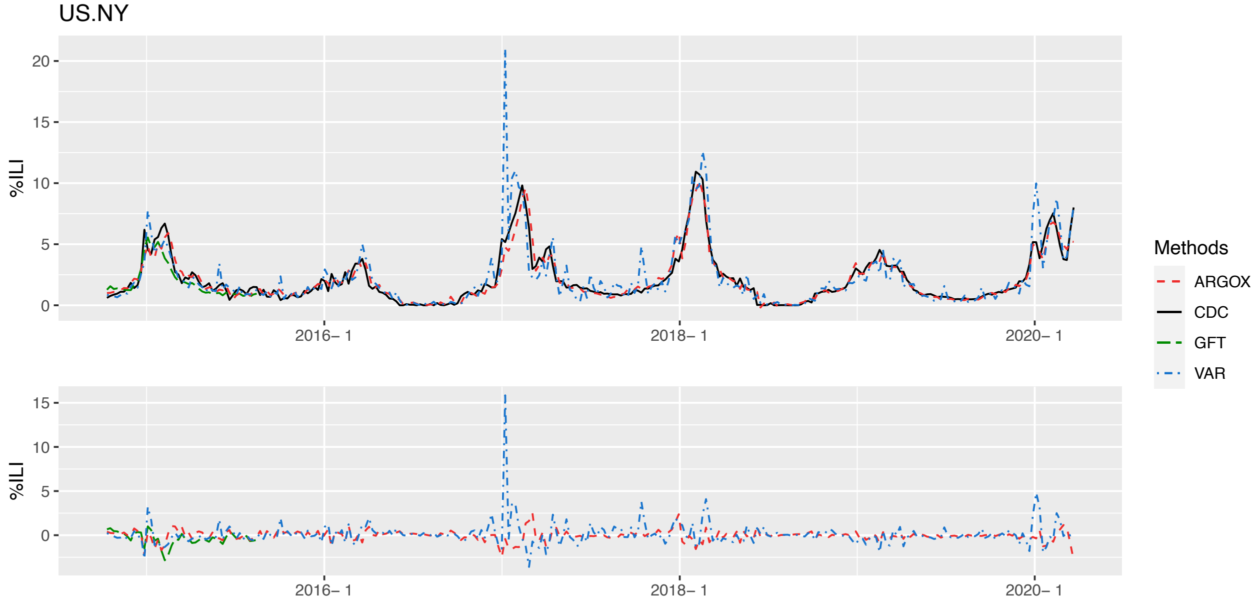

Figure S32. Plots of the %ILI estimates (top) and the estimation errors (bottom) for New York (NY).

	Whole period '14-'20	Overall '14-'17	'14-'15	'15-'16	'16-'17	'17-'18	'18-'19	'19-'20
MSE								
ARGOX	**0.246**	0.298	0.446	**0.129**	**0.449**	**0.566**	**0.129**	**0.134**
VAR	1.006	1.072	2.404	0.297	0.740	2.260	1.597	0.279
GFT	–	–	0.374	–	–	–	–	–
Lu et al. (2019)	–	**0.293**	**0.309**	0.297	0.554	–	–	–
naive	0.477	0.576	0.935	0.208	0.841	1.102	0.293	0.333
MAE								
ARGOX	**0.303**	**0.345**	**0.395**	**0.279**	**0.453**	**0.480**	**0.236**	**0.247**
VAR	0.547	0.607	0.887	0.456	0.625	0.927	0.617	0.422
GFT	–	–	0.420	–	–	–	–	–
Lu et al. (2019)	–	–	–	–	–	–	–	–
naive	0.408	0.446	0.474	0.348	0.680	0.707	0.374	0.431
Correlation								
ARGOX	**0.962**	**0.943**	0.938	**0.925**	**0.937**	**0.956**	**0.977**	**0.974**
VAR	0.874	0.769	0.569	0.831	0.890	0.954	0.911	0.957
GFT	–	–	**0.975**	–	–	–	–	–
Lu et al. (2019)	–	0.942	0.958	0.890	0.919	–	–	–
naive	0.925	0.887	0.860	0.881	0.881	0.910	0.940	0.941

Table S37. Comparison of different methods for state-level %ILI estimation in North Carolina (NC). The MSE, MAE, and correlation are reported. The method with the best performance is highlighted in boldface for each metric in each period.

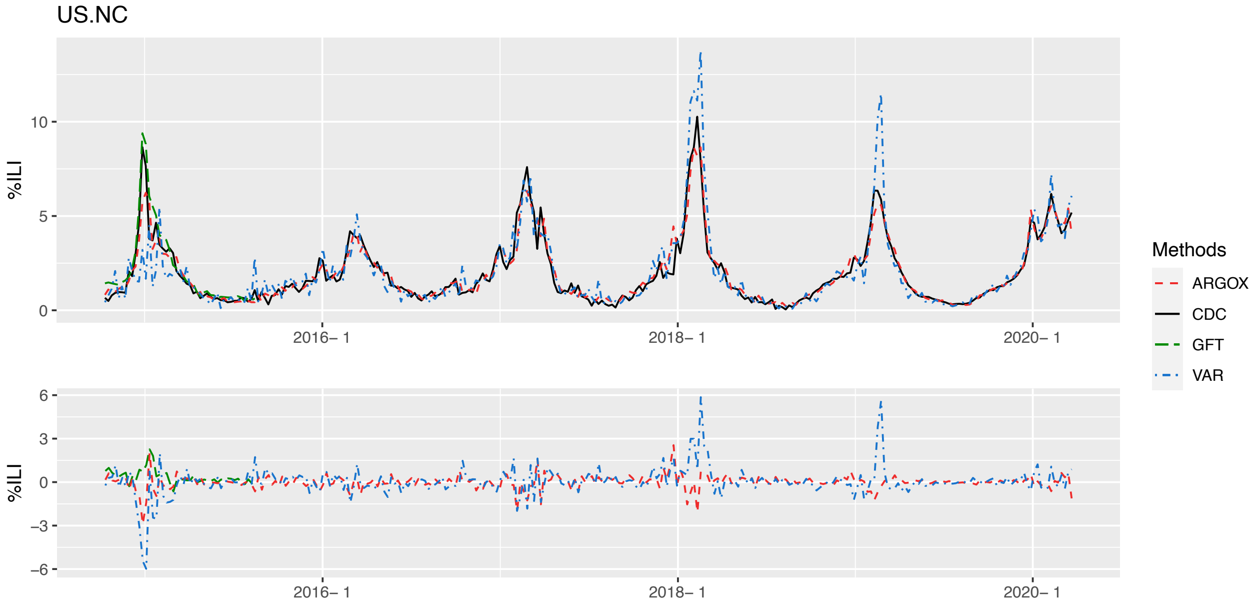

Figure S33. Plots of the %ILI estimates (top) and the estimation errors (bottom) for North Carolina (NC).

	Whole period '14-'20	Overall '14-'17	'14-'15	'15-'16	'16-'17	'17-'18	'18-'19	'19-'20
MSE								
ARGOX	**0.634**	**0.578**	**0.717**	**0.185**	1.197	**0.604**	**0.440**	**2.297**
VAR	3.213	1.877	2.947	0.544	3.125	5.011	1.006	8.291
GFT	–	–	0.938	–	–	–	–	–
Lu et al. (2019)	–	0.806	1.496	0.230	**0.927**	–	–	–
naive	0.816	0.776	0.819	0.245	1.802	0.992	0.618	2.408
MAE								
ARGOX	**0.495**	**0.438**	**0.454**	**0.351**	**0.761**	**0.609**	**0.491**	**1.121**
VAR	0.874	0.724	0.923	0.521	1.080	1.165	0.796	1.894
GFT	–	–	0.669	–	–	–	–	–
Lu et al. (2019)	–	–	–	–	–	–	–	–
naive	0.567	0.499	0.495	0.405	0.899	0.735	0.607	1.210
Correlation								
ARGOX	**0.876**	**0.839**	0.879	**0.735**	0.673	**0.846**	**0.867**	**0.778**
VAR	0.671	0.799	**0.917**	0.545	0.479	0.612	0.696	0.516
GFT	–	–	0.847	–	–	–	–	–
Lu et al. (2019)	–	0.809	0.808	0.658	**0.761**	–	–	–
naive	0.846	0.800	0.869	0.687	0.575	0.740	0.808	0.769

Table S38. Comparison of different methods for state-level %ILI estimation in North Dakota (ND). The MSE, MAE, and correlation are reported. The method with the best performance is highlighted in boldface for each metric in each period.

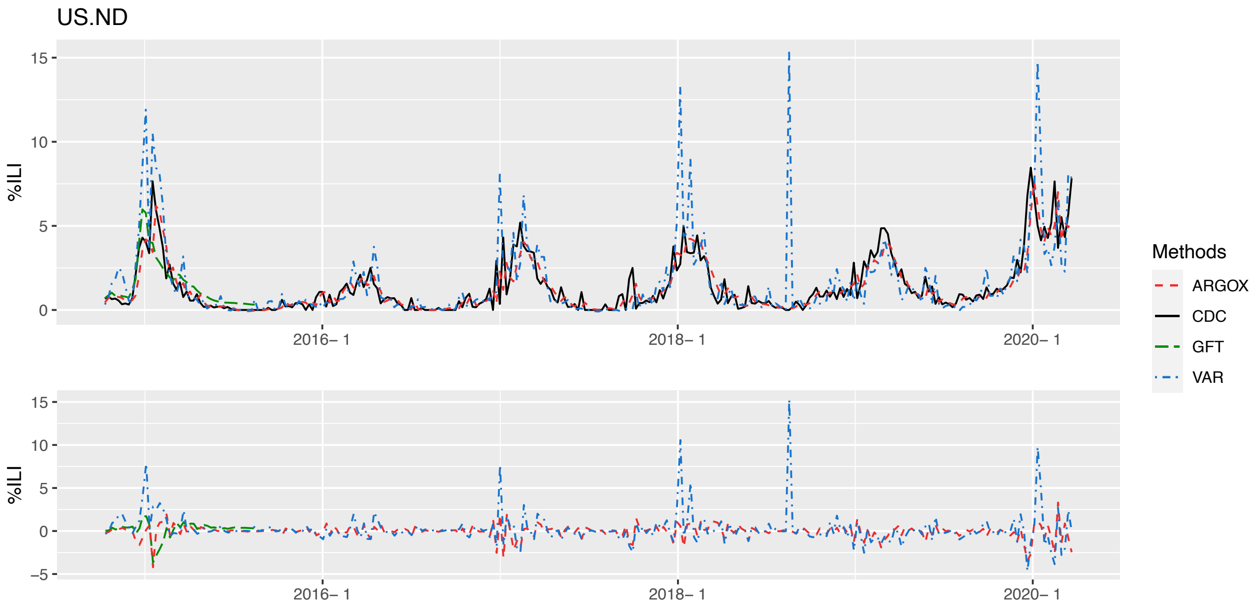

Figure S34. Plots of the %ILI estimates (top) and the estimation errors (bottom) for North Dakota (ND).

	Whole period '14-'20	Overall '14-'17	'14-'15	'15-'16	'16-'17	'17-'18	'18-'19	'19-'20
MSE								
ARGOX	**0.108**	0.121	0.291	**0.032**	**0.057**	**0.127**	**0.108**	**0.232**
VAR	0.248	0.312	0.784	0.063	0.119	0.232	0.110	0.596
GFT	–	–	0.819	–	–	–	–	–
Lu et al. (2019)	–	**0.094**	**0.236**	0.042	0.095	–	–	–
naive	0.181	0.224	0.547	0.065	0.104	0.270	0.144	0.269
MAE								
ARGOX	**0.193**	**0.179**	**0.248**	**0.136**	**0.177**	**0.253**	0.240	**0.342**
VAR	0.280	0.280	0.436	0.203	0.237	0.344	**0.233**	0.554
GFT	–	–	0.719	–	–	–	–	–
Lu et al. (2019)	–	–	–	–	–	–	–	–
naive	0.234	0.230	0.355	0.192	0.225	0.334	0.268	0.383
Correlation								
ARGOX	**0.943**	0.928	0.911	**0.915**	**0.958**	**0.959**	0.890	**0.922**
VAR	0.885	0.846	0.835	0.829	0.919	0.957	**0.905**	0.784
GFT	–	–	**0.971**	–	–	–	–	–
Lu et al. (2019)	–	**0.943**	0.941	0.897	0.932	–	–	–
naive	0.906	0.869	0.833	0.835	0.922	0.912	0.860	0.915

Table S39. Comparison of different methods for state-level %ILI estimation in Ohio (OH). The MSE, MAE, and correlation are reported. The method with the best performance is highlighted in boldface for each metric in each period.

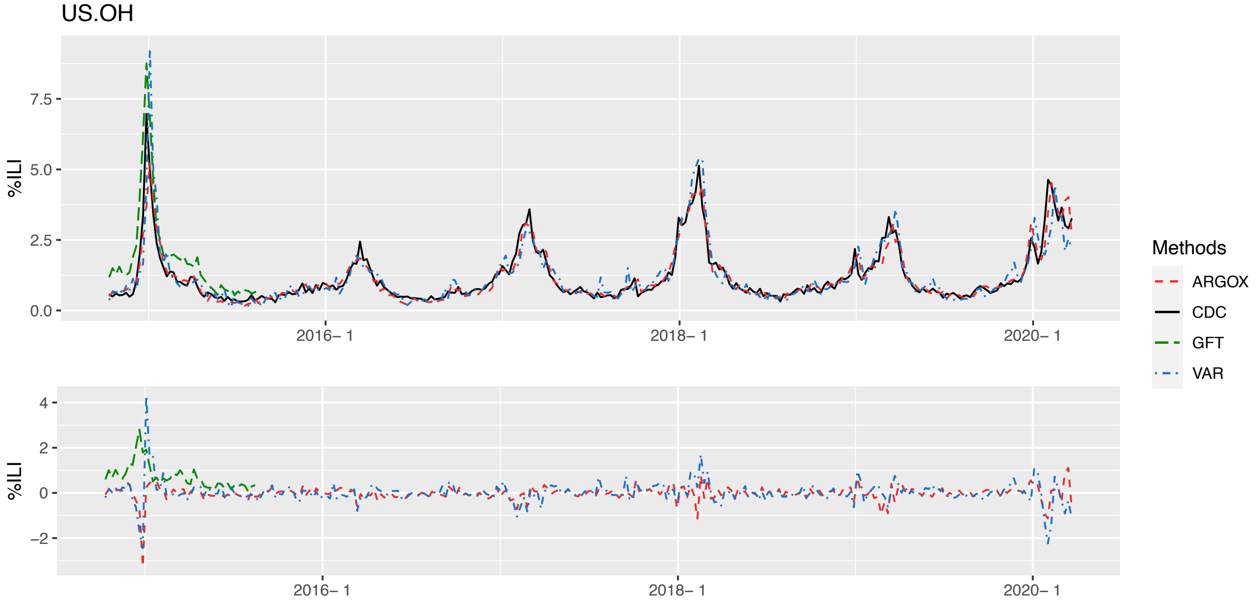

Figure S35. Plots of the %ILI estimates (top) and the estimation errors (bottom) for Ohio (OH).

	Whole period '14-'20	Overall '14-'17	'14-'15	'15-'16	'16-'17	'17-'18	'18-'19	'19-'20
MSE								
ARGOX	**0.859**	1.360	2.003	**0.726**	**2.002**	**0.554**	**0.539**	**0.701**
VAR	8.691	4.993	3.314	2.556	13.327	5.961	9.482	52.931
GFT	–	–	3.906	–	–	–	–	–
Lu et al. (2019)	–	–	–	–	–	–	–	–
naive	0.965	**1.291**	**1.692**	0.786	2.124	1.000	0.988	1.063
MAE								
ARGOX	**0.586**	0.788	0.924	**0.695**	**1.052**	**0.516**	**0.501**	**0.573**
VAR	1.415	1.282	1.186	1.253	2.139	1.349	1.956	4.176
GFT	–	–	1.390	–	–	–	–	–
Lu et al. (2019)	–	–	–	–	–	–	–	–
naive	0.646	**0.775**	**0.889**	0.716	1.091	0.745	0.706	0.742
Correlation								
ARGOX	**0.956**	0.933	0.917	0.532	**0.935**	**0.980**	**0.971**	**0.959**
VAR	0.808	0.803	0.858	0.493	0.761	0.914	0.890	0.610
GFT	–	–	0.856	–	–	–	–	–
Lu et al. (2019)	–	–	–	–	–	–	–	–
naive	0.951	**0.938**	**0.930**	**0.535**	0.931	0.943	0.940	0.943

Table S40. Comparison of different methods for state-level %ILI estimation in Oklahoma (OK). The MSE, MAE, and correlation are reported. The method with the best performance is highlighted in boldface for each metric in each period.

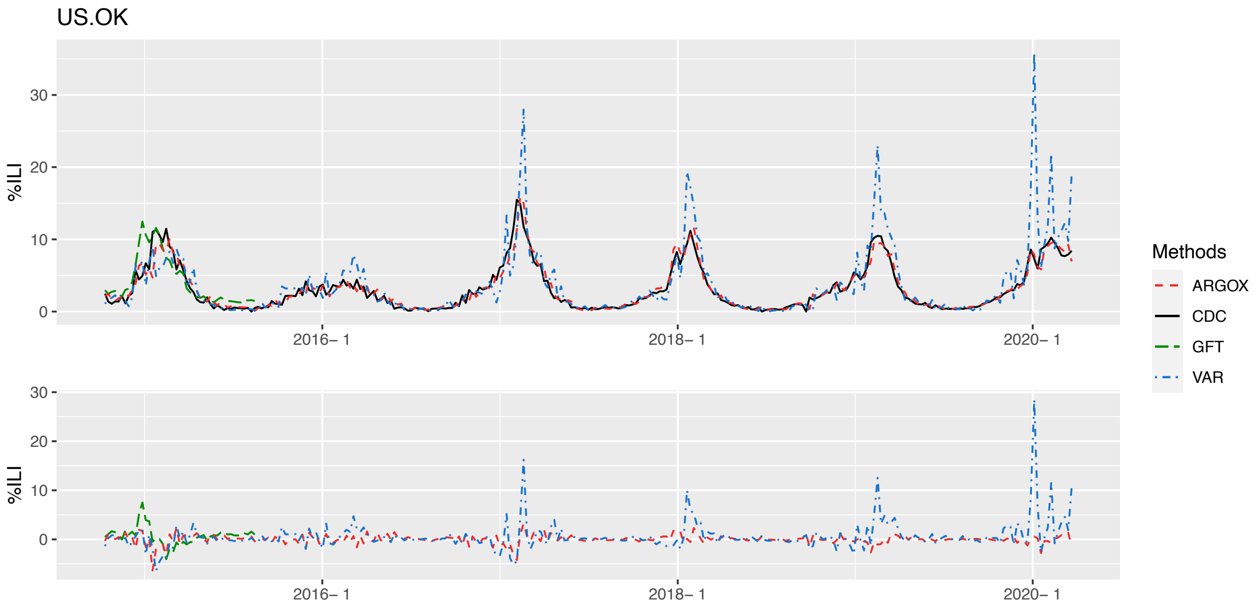

Figure S36. Plots of the %ILI estimates (top) and the estimation errors (bottom) for Oklahoma (OK).

	Whole period '14-'20	Overall '14-'17	'14-'15	'15-'16	'16-'17	'17-'18	'18-'19	'19-'20
MSE								
ARGOX	**0.187**	**0.286**	0.123	0.358	**0.624**	**0.062**	**0.157**	**0.240**
VAR	1.188	0.616	0.281	0.559	1.463	6.474	0.349	0.717
GFT	–	–	0.372	–	–	–	–	–
Lu et al. (2019)	–	0.408	0.253	**0.350**	0.869	–	–	–
naive	0.260	0.352	**0.094**	0.400	0.894	0.190	0.203	0.470
MAE								
ARGOX	**0.264**	**0.327**	0.254	0.435	**0.457**	**0.187**	**0.273**	**0.364**
VAR	0.522	0.480	0.356	0.598	0.716	1.156	0.436	0.636
GFT	–	–	0.516	–	–	–	–	–
Lu et al. (2019)	–	–	–	–	–	–	–	–
naive	0.300	0.341	**0.237**	**0.428**	0.560	0.310	0.275	0.534
Correlation								
ARGOX	**0.945**	**0.856**	0.803	0.715	**0.796**	**0.988**	**0.941**	**0.958**
VAR	0.794	0.739	0.745	0.591	0.702	0.825	0.878	0.872
GFT	–	–	0.739	–	–	–	–	–
Lu et al. (2019)	–	0.802	0.779	**0.750**	0.745	–	–	–
naive	0.924	0.832	**0.829**	0.713	0.728	0.952	0.919	0.920

Table S41. Comparison of different methods for state-level %ILI estimation in Oregon (OR). The MSE, MAE, and correlation are reported. The method with the best performance is highlighted in boldface for each metric in each period.

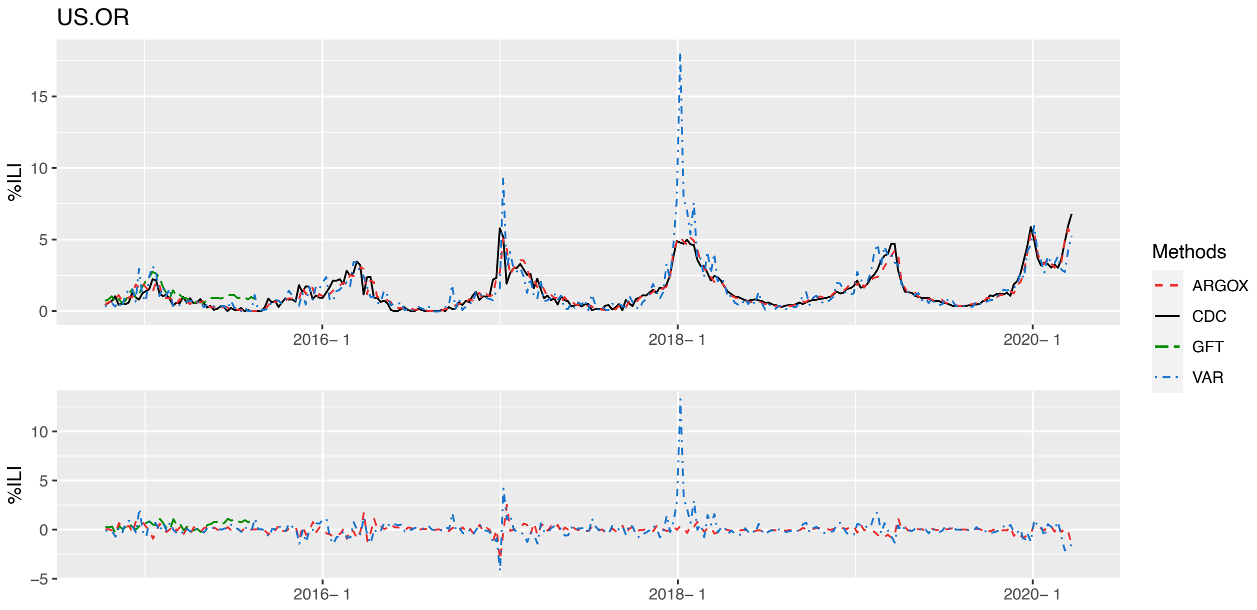

Figure S37. Plots of the %ILI estimates (top) and the estimation errors (bottom) for Oregon (OR).

	Whole period '14-'20	Overall '14-'17	'14-'15	'15-'16	'16-'17	'17-'18	'18-'19	'19-'20
MSE								
ARGOX	**0.124**	0.157	0.236	**0.092**	**0.202**	**0.165**	**0.035**	**0.274**
VAR	0.311	0.511	1.013	0.224	0.446	0.167	0.106	0.342
GFT	–	–	0.329	–	–	–	–	–
Lu et al. (2019)	–	**0.138**	**0.214**	0.110	0.218	–	–	–
naive	0.227	0.307	0.523	0.206	0.318	0.319	0.082	0.357
MAE								
ARGOX	**0.215**	**0.266**	**0.314**	**0.224**	**0.325**	**0.231**	**0.149**	**0.305**
VAR	0.322	0.420	0.561	0.324	0.473	0.302	0.230	0.421
GFT	–	–	0.473	–	–	–	–	–
Lu et al. (2019)	–	–	–	–	–	–	–	–
naive	0.295	0.361	0.467	0.336	0.396	0.358	0.212	0.440
Correlation								
ARGOX	**0.967**	0.947	0.954	0.899	0.934	**0.978**	**0.977**	**0.950**
VAR	0.925	0.871	0.866	0.810	0.866	0.973	0.934	0.935
GFT	–	–	0.950	–	–	–	–	–
Lu et al. (2019)	–	**0.954**	**0.963**	**0.905**	**0.935**	–	–	–
naive	0.938	0.898	0.889	0.793	0.900	0.945	0.943	0.938

Table S42. Comparison of different methods for state-level %ILI estimation in Pennsylvania (PA). The MSE, MAE, and correlation are reported. The method with the best performance is highlighted in boldface for each metric in each period.

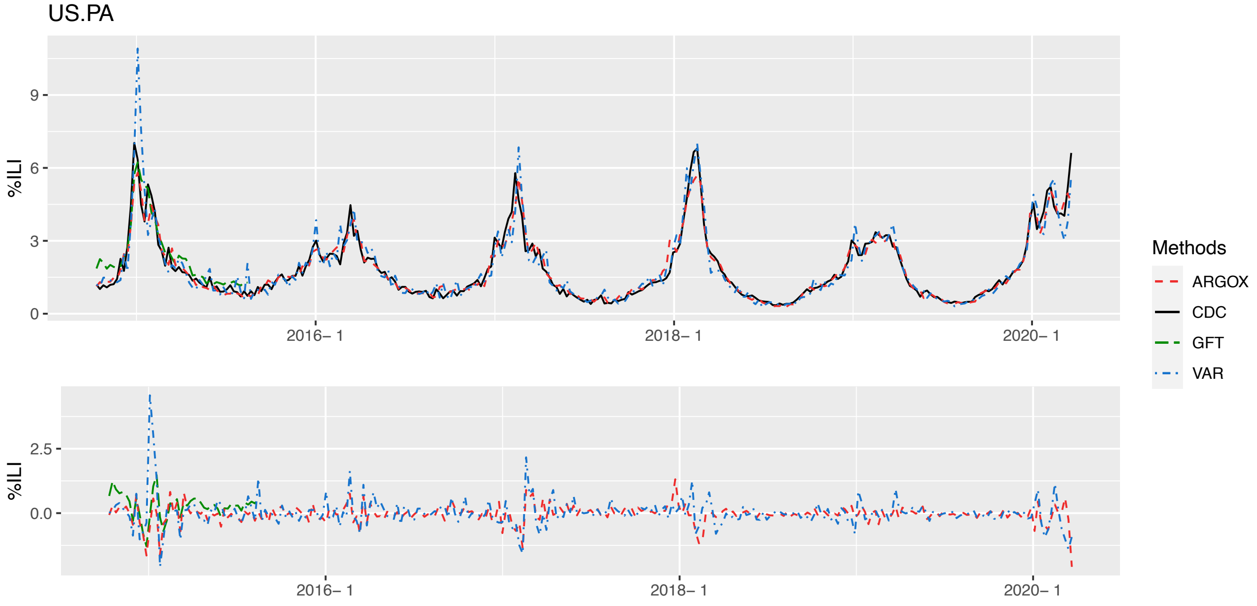

Figure S38. Plots of the %ILI estimates (top) and the estimation errors (bottom) for Pennsylvania (PA).

	Whole period '14-'20	Overall '14-'17	'14-'15	'15-'16	'16-'17	'17-'18	'18-'19	'19-'20
MSE								
ARGOX	**0.235**	0.144	0.194	0.056	0.212	**0.390**	**0.283**	**0.998**
VAR	0.858	0.272	0.349	0.129	0.423	2.802	2.542	1.130
GFT	–	–	0.417	–	–	–	–	–
Lu et al. (2019)	–	**0.067**	**0.117**	**0.025**	**0.018**	–	–	–
naive	0.307	0.166	0.201	0.057	0.294	0.661	0.428	1.150
MAE								
ARGOX	**0.276**	**0.223**	**0.243**	0.178	**0.314**	**0.438**	**0.368**	**0.682**
VAR	0.433	0.313	0.313	0.248	0.494	0.818	0.894	0.737
GFT	–	–	0.565	–	–	–	–	–
Lu et al. (2019)	–	–	–	–	–	–	–	–
naive	0.314	0.232	0.257	**0.153**	0.365	0.564	0.490	0.750
Correlation								
ARGOX	**0.961**	0.932	0.923	0.905	**0.940**	**0.954**	**0.959**	0.942
VAR	0.901	0.882	0.862	0.858	0.880	0.873	0.752	**0.952**
GFT	–	–	0.944	–	–	–	–	–
Lu et al. (2019)	–	**0.949**	**0.956**	**0.961**	0.047	–	–	–
naive	0.949	0.919	0.916	0.907	0.904	0.918	0.936	0.936

Table S43. Comparison of different methods for state-level %ILI estimation in Rhode Island (RI). The MSE, MAE, and correlation are reported. The method with the best performance is highlighted in boldface for each metric in each period.

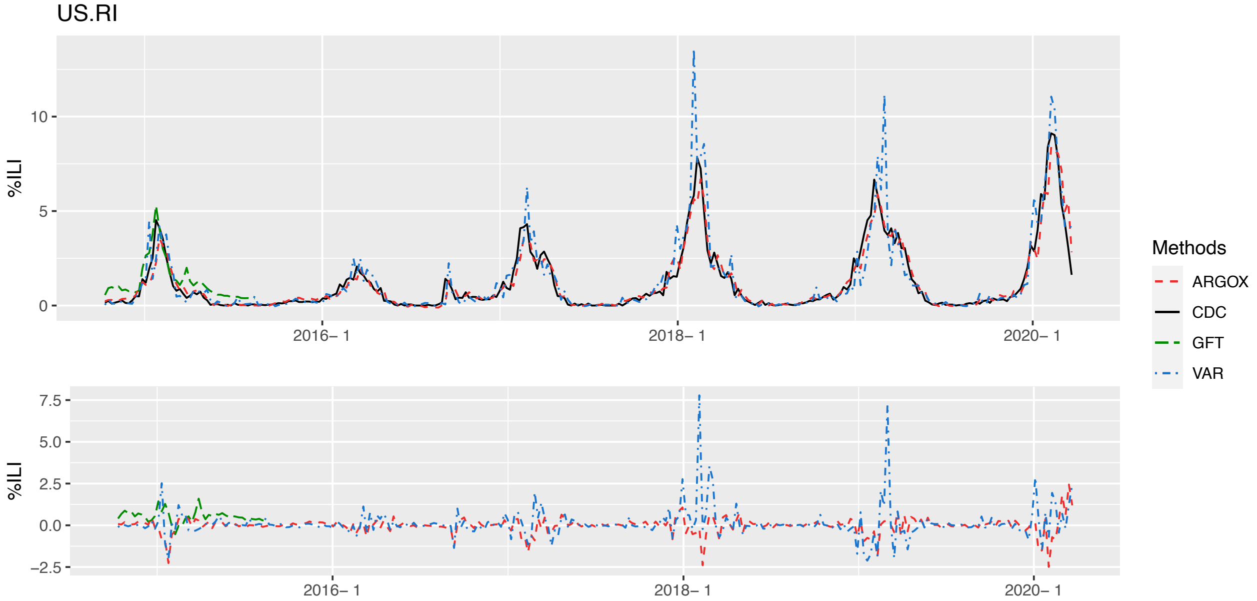

Figure S39. Plots of the %ILI estimates (top) and the estimation errors (bottom) for Rhode Island (RI).

	Whole period '14-'20	Overall '14-'17	'14-'15	'15-'16	'16-'17	'17-'18	'18-'19	'19-'20
MSE								
ARGOX	**0.351**	**0.336**	0.317	**0.097**	**0.864**	**0.472**	**0.427**	**0.835**
VAR	1.932	0.912	0.963	0.342	2.115	8.423	1.260	3.850
GFT	–	–	2.269	–	–	–	–	–
Lu et al. (2019)	–	0.383	**0.130**	0.452	0.955	–	–	–
naive	0.767	0.465	0.565	0.141	1.013	1.790	0.871	2.547
MAE								
ARGOX	**0.367**	**0.348**	**0.285**	**0.234**	**0.759**	**0.455**	**0.432**	**0.726**
VAR	0.687	0.531	0.436	0.459	1.085	1.772	0.741	1.029
GFT	–	–	1.235	–	–	–	–	–
Lu et al. (2019)	–	–	–	–	–	–	–	–
naive	0.516	0.403	0.387	0.292	0.795	0.880	0.683	1.172
Correlation								
ARGOX	**0.979**	**0.953**	0.925	**0.873**	0.909	**0.985**	**0.963**	**0.956**
VAR	0.906	0.882	0.816	0.574	0.811	0.754	0.922	0.918
GFT	–	–	0.978	–	–	–	–	–
Lu et al. (2019)	–	0.951	**0.983**	0.639	**0.918**	–	–	–
naive	0.954	0.934	0.860	0.823	0.888	0.941	0.915	0.874

Table S44. Comparison of different methods for state-level %ILI estimation in South Carolina (SC). The MSE, MAE, and correlation are reported. The method with the best performance is highlighted in boldface for each metric in each period.

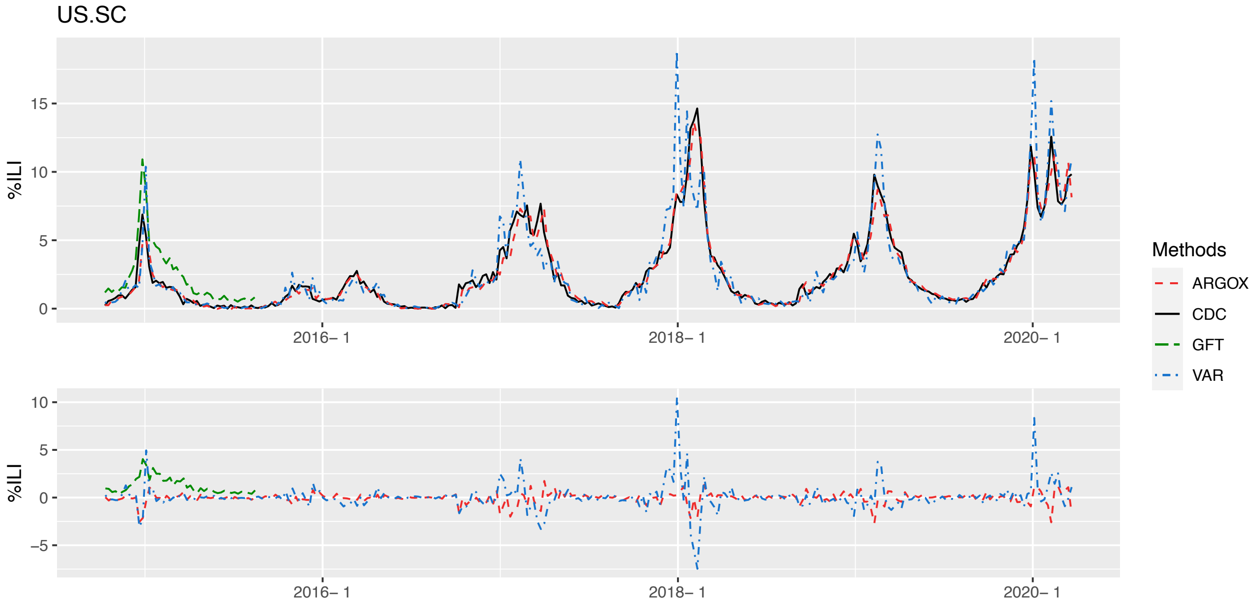

Figure S40. Plots of the %ILI estimates (top) and the estimation errors (bottom) for South Carolina (SC).

	Whole period ’14-’20	Overall ’14-’17	’14-’15	’15-’16	’16-’17	’17-’18	’18-’19	’19-’20
MSE								
ARGOX	**0.104**	0.099	**0.087**	**0.087**	0.166	**0.224**	**0.126**	**0.081**
VAR	0.331	0.372	0.363	0.371	0.594	0.250	0.228	0.992
GFT	–	–	0.953	–	–	–	–	–
Lu et al. (2019)	–	**0.082**	0.093	0.109	**0.102**	–	–	–
naive	0.124	0.117	0.117	0.095	0.182	0.285	0.141	0.095
MAE								
ARGOX	**0.231**	**0.226**	**0.215**	**0.215**	**0.289**	**0.347**	**0.280**	**0.218**
VAR	0.373	0.397	0.405	0.421	0.497	0.395	0.369	0.629
GFT	–	–	0.476	–	–	–	–	–
Lu et al. (2019)	–	–	–	–	–	–	–	–
naive	0.256	0.252	0.239	0.244	0.318	0.416	0.284	0.220
Correlation								
ARGOX	**0.952**	0.929	**0.932**	**0.849**	0.929	**0.930**	**0.884**	**0.973**
VAR	0.880	0.828	0.828	0.674	0.832	0.922	0.725	0.843
GFT	–	–	0.906	–	–	–	–	–
Lu et al. (2019)	–	**0.941**	0.926	0.805	**0.956**	–	–	–
naive	0.943	0.918	0.907	0.840	0.924	0.908	0.844	0.970

Table S45. Comparison of different methods for state-level %ILI estimation in South Dakota (SD). The MSE, MAE, and correlation are reported. The method with the best performance is highlighted in boldface for each metric in each period.

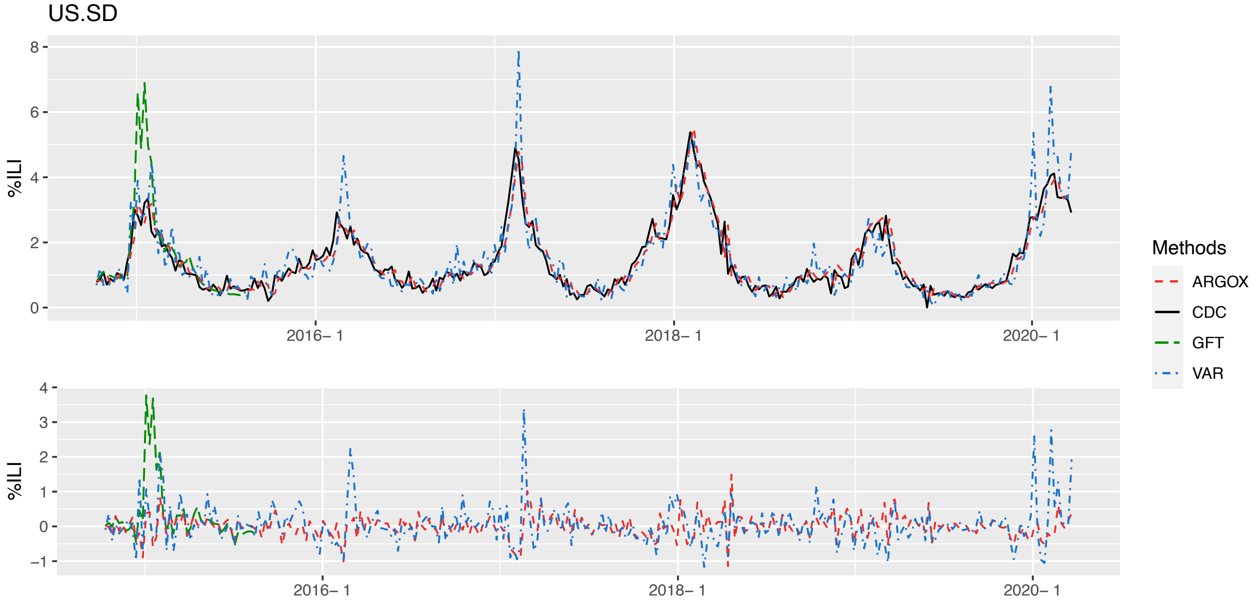

Figure S41. Plots of the %ILI estimates (top) and the estimation errors (bottom) for South Dakota (SD).

	Whole period '14-'20	Overall '14-'17	'14-'15	'15-'16	'16-'17	'17-'18	'18-'19	'19-'20
MSE								
ARGOX	**0.522**	**0.369**	**0.321**	**0.204**	0.740	**1.020**	**0.239**	**2.122**
VAR	1.752	1.695	2.354	0.767	2.342	3.022	1.295	4.688
GFT	–	–	1.407	–	–	–	–	–
Lu et al. (2019)	–	0.391	0.440	0.282	**0.717**	–	–	–
naive	0.748	0.568	0.705	0.257	0.983	1.427	0.500	2.811
MAE								
ARGOX	**0.471**	**0.435**	**0.377**	0.378	**0.644**	**0.613**	**0.379**	**1.138**
VAR	0.726	0.776	0.729	0.633	1.079	0.787	0.713	1.469
GFT	–	–	0.947	–	–	–	–	–
Lu et al. (2019)	–	–	–	–	–	–	–	–
naive	0.509	0.481	0.480	**0.358**	0.711	0.711	0.522	1.142
Correlation								
ARGOX	**0.950**	**0.936**	0.956	**0.867**	0.864	**0.916**	**0.941**	**0.908**
VAR	0.853	0.797	0.867	0.684	0.671	0.813	0.824	0.803
GFT	–	–	**0.968**	–	–	–	–	–
Lu et al. (2019)	–	0.936	0.953	0.841	**0.890**	–	–	–
naive	0.929	0.900	0.898	0.836	0.822	0.869	0.869	0.881

Table S46. Comparison of different methods for state-level %ILI estimation in Tennessee (TN). The MSE, MAE, and correlation are reported. The method with the best performance is highlighted in boldface for each metric in each period.

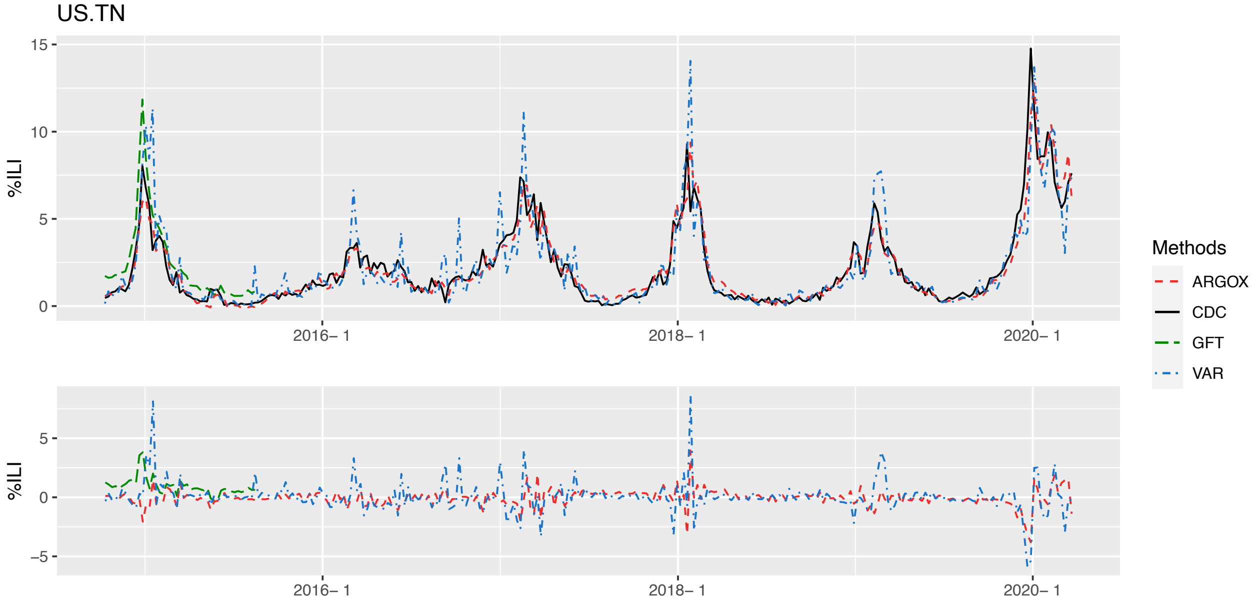

Figure S42. Plots of the %ILI estimates (top) and the estimation errors (bottom) for Tennessee (TN).

	Whole period '14-'20	Overall '14-'17	'14-'15	'15-'16	'16-'17	'17-'18	'18-'19	'19-'20
MSE								
ARGOX	**0.755**	0.831	1.824	0.347	**0.518**	**1.066**	**0.364**	**2.081**
VAR	1.783	1.736	3.191	1.162	1.448	3.269	2.243	3.249
GFT	–	–	**1.403**	–	–	–	–	–
Lu et al. (2019)	–	**0.783**	2.074	0.432	0.562	–	–	–
naive	0.997	0.958	2.121	**0.333**	0.633	1.898	0.676	2.585
MAE								
ARGOX	**0.510**	**0.537**	**0.759**	0.466	**0.540**	**0.635**	**0.419**	**1.054**
VAR	0.738	0.757	0.961	0.709	0.829	0.959	0.858	1.152
GFT	–	–	0.772	–	–	–	–	–
Lu et al. (2019)	–	–	–	–	–	–	–	–
naive	0.598	0.563	0.801	**0.454**	0.577	0.936	0.634	1.177
Correlation								
ARGOX	**0.959**	0.914	0.882	0.812	**0.959**	**0.972**	**0.976**	**0.908**
VAR	0.920	0.839	0.827	0.641	0.866	0.937	0.931	0.876
GFT	–	–	**0.936**	–	–	–	–	–
Lu et al. (2019)	–	**0.919**	0.870	0.791	0.954	–	–	–
naive	0.947	0.905	0.869	**0.816**	0.945	0.951	0.946	0.887

Table S47. Comparison of different methods for state-level %ILI estimation in Texas (TX). The MSE, MAE, and correlation are reported. The method with the best performance is highlighted in boldface for each metric in each period.

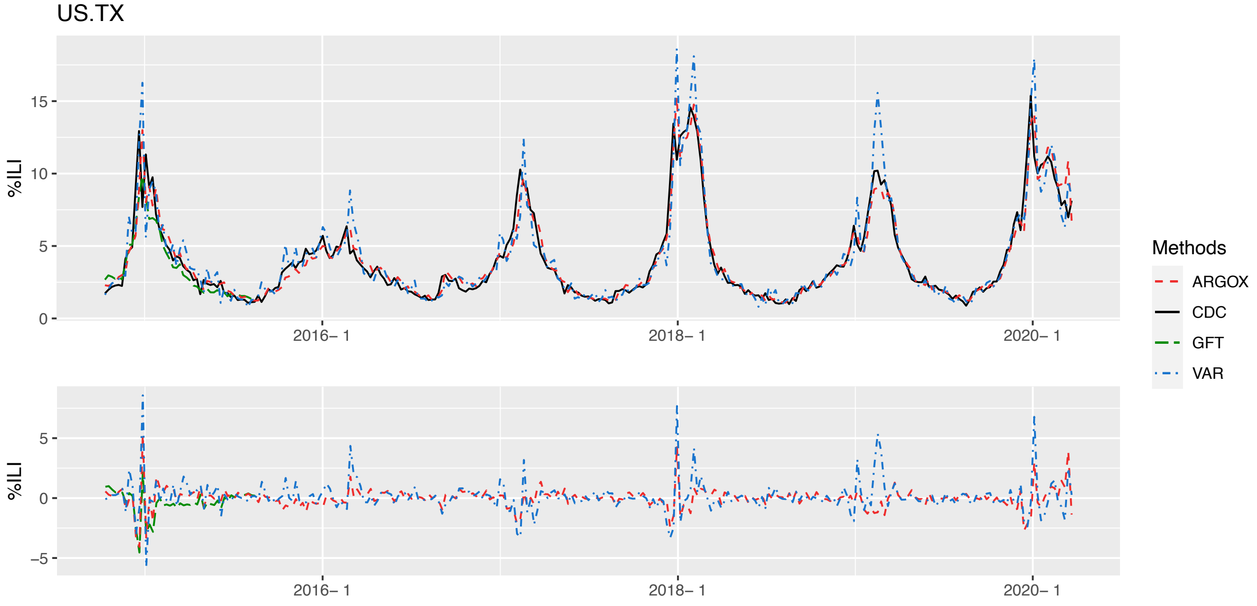

Figure S43. Plots of the %ILI estimates (top) and the estimation errors (bottom) for Texas (TX).

	Whole period '14-'20	Overall '14-'17	'14-'15	'15-'16	'16-'17	'17-'18	'18-'19	'19-'20
MSE								
ARGOX	**0.202**	**0.125**	**0.092**	**0.129**	0.221	**0.142**	**0.393**	**0.773**
VAR	0.875	0.742	0.960	1.257	0.363	0.232	1.888	2.574
GFT	–	–	1.059	–	–	–	–	–
Lu et al. (2019)	–	0.185	0.235	0.221	0.217	–	–	–
naive	0.255	0.182	0.238	0.184	**0.193**	0.197	0.433	0.938
MAE								
ARGOX	**0.305**	**0.253**	**0.224**	**0.281**	0.321	**0.286**	**0.476**	**0.672**
VAR	0.633	0.594	0.698	0.881	0.392	0.356	1.086	1.313
GFT	–	–	0.930	–	–	–	–	–
Lu et al. (2019)	–	–	–	–	–	–	–	–
naive	0.334	0.293	0.334	0.329	**0.289**	0.298	0.515	0.730
Correlation								
ARGOX	**0.961**	**0.946**	**0.972**	**0.917**	0.883	**0.900**	**0.934**	**0.915**
VAR	0.840	0.818	0.795	0.698	0.858	0.847	0.704	0.720
GFT	–	–	0.949	–	–	–	–	–
Lu et al. (2019)	–	0.921	0.930	0.849	0.880	–	–	–
naive	0.951	0.923	0.923	0.885	**0.889**	0.868	0.921	0.900

Table S48. Comparison of different methods for state-level %ILI estimation in Utah (UT). The MSE, MAE, and correlation are reported. The method with the best performance is highlighted in boldface for each metric in each period.

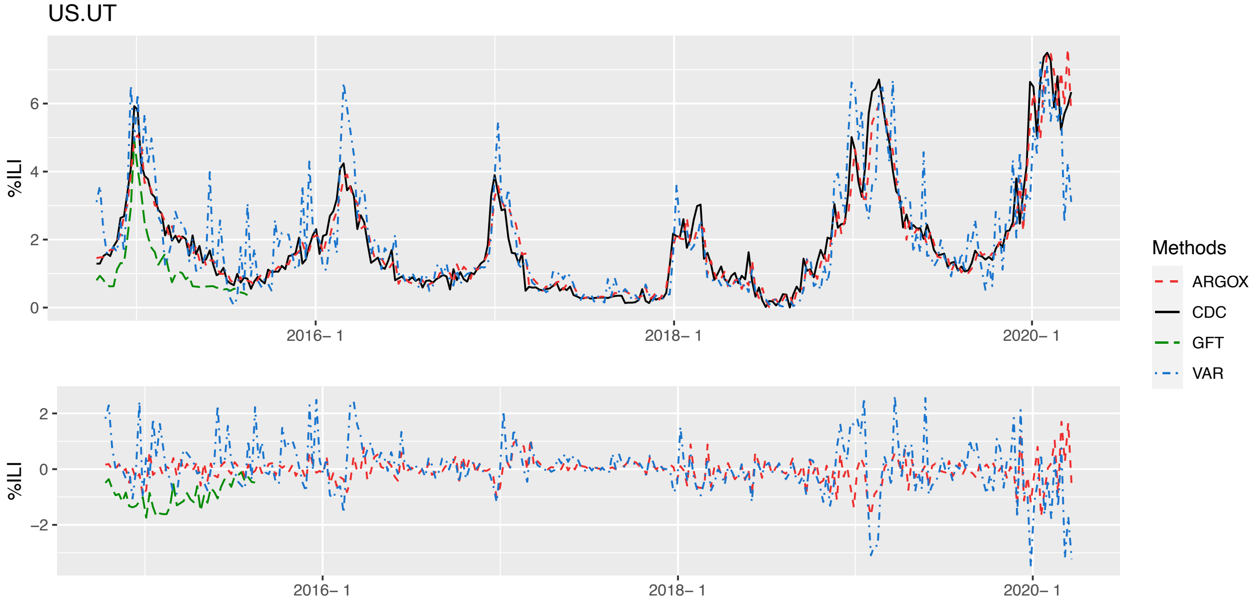

Figure S44. Plots of the %ILI estimates (top) and the estimation errors (bottom) for Utah (UT).

	Whole period '14-'20	Overall '14-'17	'14-'15	'15-'16	'16-'17	'17-'18	'18-'19	'19-'20
MSE								
ARGOX	**0.241**	**0.265**	**0.513**	0.181	0.101	**0.284**	**0.287**	**0.261**
VAR	0.579	0.597	0.655	0.692	0.597	0.801	0.967	0.355
GFT	–	–	1.330	–	–	–	–	–
Lu et al. (2019)	–	0.317	0.845	**0.176**	**0.101**	–	–	–
naive	0.286	0.315	0.606	0.220	0.127	0.333	0.348	0.323
MAE								
ARGOX	**0.349**	**0.355**	**0.492**	**0.325**	**0.252**	**0.407**	**0.409**	**0.342**
VAR	0.558	0.580	0.615	0.661	0.510	0.670	0.727	0.471
GFT	–	–	0.761	–	–	–	–	–
Lu et al. (2019)	–	–	–	–	–	–	–	–
naive	0.380	0.384	0.521	0.360	0.264	0.451	0.447	0.400
Correlation								
ARGOX	**0.918**	**0.902**	**0.914**	0.789	**0.799**	**0.920**	**0.880**	**0.946**
VAR	0.824	0.790	0.883	0.609	0.478	0.840	0.683	0.928
GFT	–	–	0.851	–	–	–	–	–
Lu et al. (2019)	–	0.889	0.845	**0.833**	0.782	–	–	–
naive	0.905	0.886	0.896	0.753	0.754	0.909	0.859	0.939

Table S49. Comparison of different methods for state-level %ILI estimation in Vermont (VT). The MSE, MAE, and correlation are reported. The method with the best performance is highlighted in boldface for each metric in each period.

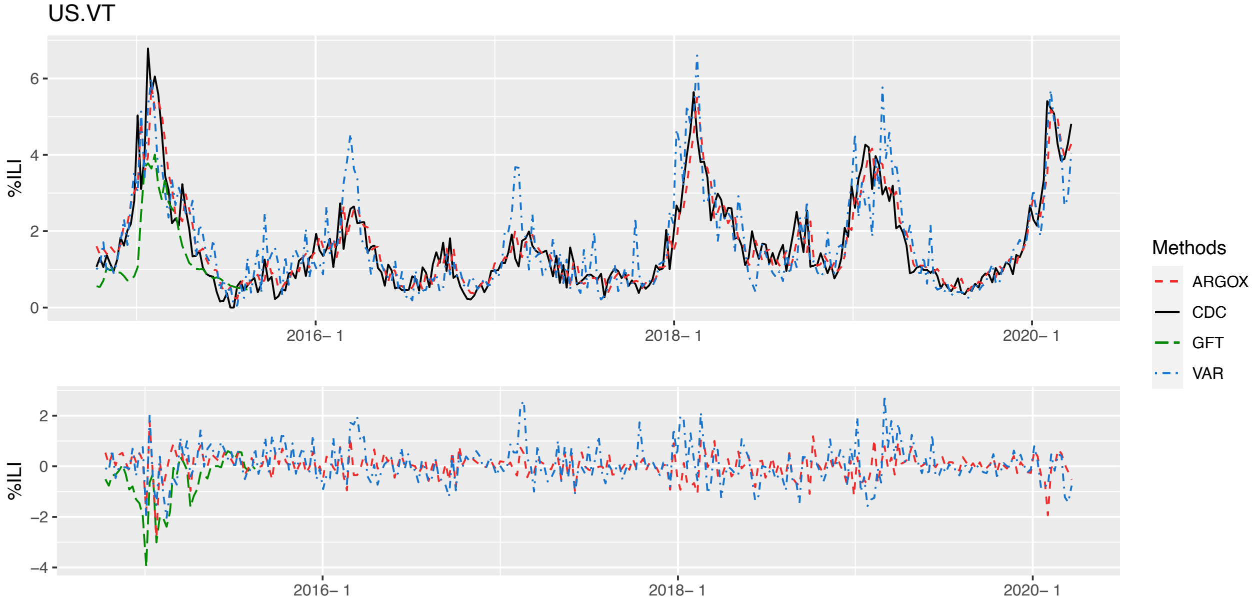

Figure S45. Plots of the %ILI estimates (top) and the estimation errors (bottom) for Vermont (VT).

	Whole period '14-'20	Overall '14-'17	'14-'15	'15-'16	'16-'17	'17-'18	'18-'19	'19-'20
MSE								
ARGOX	**0.169**	0.178	0.329	0.058	0.214	**0.376**	**0.203**	**0.163**
VAR	0.325	0.263	0.438	0.048	0.408	0.651	0.297	0.975
GFT	–	–	0.984	–	–	–	–	–
Lu et al. (2019)	–	**0.108**	**0.211**	**0.024**	**0.183**	–	–	–
naive	0.396	0.389	0.798	0.084	0.417	0.952	0.314	0.695
MAE								
ARGOX	**0.238**	**0.232**	**0.287**	0.181	**0.307**	**0.400**	**0.316**	**0.310**
VAR	0.327	0.299	0.373	**0.159**	0.455	0.518	0.387	0.609
GFT	–	–	0.497	–	–	–	–	–
Lu et al. (2019)	–	–	–	–	–	–	–	–
naive	0.335	0.310	0.418	0.240	0.381	0.595	0.411	0.643
Correlation								
ARGOX	**0.982**	0.959	0.960	0.923	0.943	**0.983**	**0.972**	**0.987**
VAR	0.965	0.946	0.956	0.937	0.892	0.960	0.959	0.929
GFT	–	–	0.977	–	–	–	–	–
Lu et al. (2019)	–	**0.975**	**0.983**	**0.970**	**0.950**	–	–	–
naive	0.955	0.907	0.895	0.889	0.885	0.941	0.956	0.948

Table S50. Comparison of different methods for state-level %ILI estimation in Virginia (VA). The MSE, MAE, and correlation are reported. The method with the best performance is highlighted in boldface for each metric in each period.

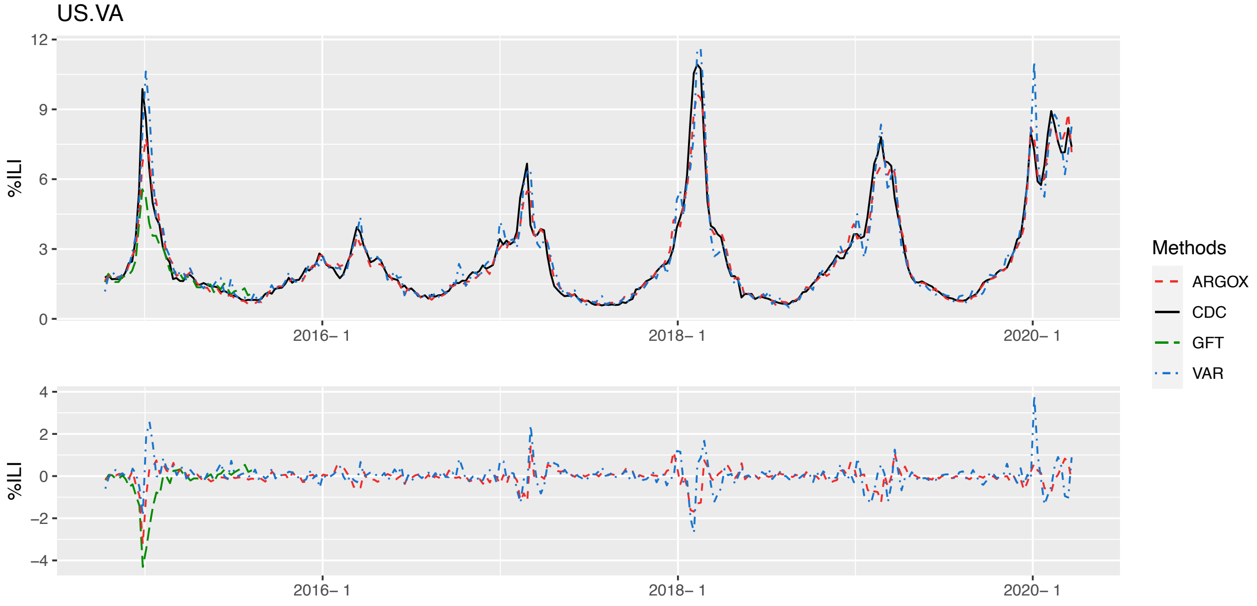

Figure S46. Plots of the %ILI estimates (top) and the estimation errors (bottom) for Virginia (VA).

	Whole period '14-'20	Overall '14-'17	'14-'15	'15-'16	'16-'17	'17-'18	'18-'19	'19-'20
MSE								
ARGOX	**0.192**	0.141	0.122	**0.092**	0.312	**0.411**	**0.211**	**0.552**
VAR	0.561	0.257	0.305	0.213	0.401	0.558	0.322	3.864
GFT	–	–	0.562	–	–	–	–	–
Lu et al. (2019)	–	**0.115**	**0.076**	0.115	**0.269**	–	–	–
naive	0.263	0.157	0.145	0.114	0.322	0.551	0.302	0.966
MAE								
ARGOX	**0.261**	**0.242**	**0.237**	**0.252**	0.352	**0.325**	**0.289**	**0.585**
VAR	0.406	0.337	0.396	0.364	0.383	0.450	0.329	1.431
GFT	–	–	0.654	–	–	–	–	–
Lu et al. (2019)	–	–	–	–	–	–	–	–
naive	0.304	0.262	0.277	0.283	**0.352**	0.415	0.358	0.723
Correlation								
ARGOX	**0.954**	0.901	0.939	**0.842**	0.825	**0.883**	**0.914**	**0.951**
VAR	0.861	0.836	0.867	0.604	0.796	0.848	0.882	0.698
GFT	–	–	0.963	–	–	–	–	–
Lu et al. (2019)	–	**0.913**	**0.967**	0.798	0.823	–	–	–
naive	0.937	0.894	0.928	0.809	**0.834**	0.850	0.876	0.902

Table S51. Comparison of different methods for state-level %ILI estimation in Washington (WA). The MSE, MAE, and correlation are reported. The method with the best performance is highlighted in boldface for each metric in each period.

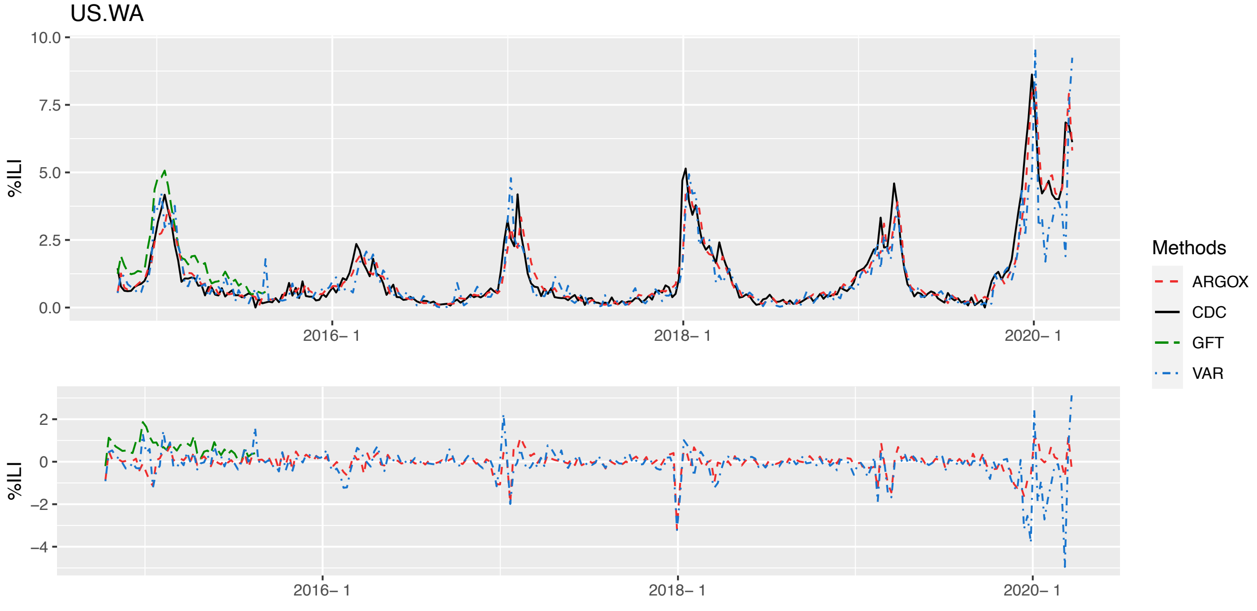

Figure S47. Plots of the %ILI estimates (top) and the estimation errors (bottom) for Washington (WA).

	Whole period ’14-’20	Overall ’14-’17	’14-’15	’15-’16	’16-’17	’17-’18	’18-’19	’19-’20
MSE								
ARGOX	**0.264**	**0.171**	0.303	**0.119**	**0.140**	**0.702**	**0.383**	**0.605**
VAR	0.600	0.307	0.341	0.222	0.523	1.791	0.489	2.093
GFT	–	–	0.974	–	–	–	–	–
Lu et al. (2019)	–	0.179	**0.264**	0.185	–	–	–	–
naive	0.413	0.261	0.520	0.153	0.181	1.212	0.478	1.000
MAE								
ARGOX	**0.314**	**0.285**	**0.345**	**0.274**	0.302	**0.536**	**0.417**	**0.518**
VAR	0.458	0.385	0.405	0.355	0.529	0.778	0.522	1.101
GFT	–	–	0.909	–	–	–	–	–
Lu et al. (2019)	–	–	–	–	–	–	–	–
naive	0.355	0.317	0.442	0.281	**0.298**	0.649	0.437	0.645
Correlation								
ARGOX	**0.957**	0.958	0.967	**0.826**	**0.939**	**0.929**	**0.925**	**0.943**
VAR	0.920	0.939	0.964	0.787	0.896	0.894	0.909	0.835
GFT	–	–	0.969	–	–	–	–	–
Lu et al. (2019)	–	**0.965**	**0.979**	0.793	–	–	–	–
naive	0.933	0.933	0.936	0.798	0.921	0.876	0.907	0.912

Table S52. Comparison of different methods for state-level %ILI estimation in West Virginia (WV). The MSE, MAE, and correlation are reported. The method with the best performance is highlighted in boldface for each metric in each period.

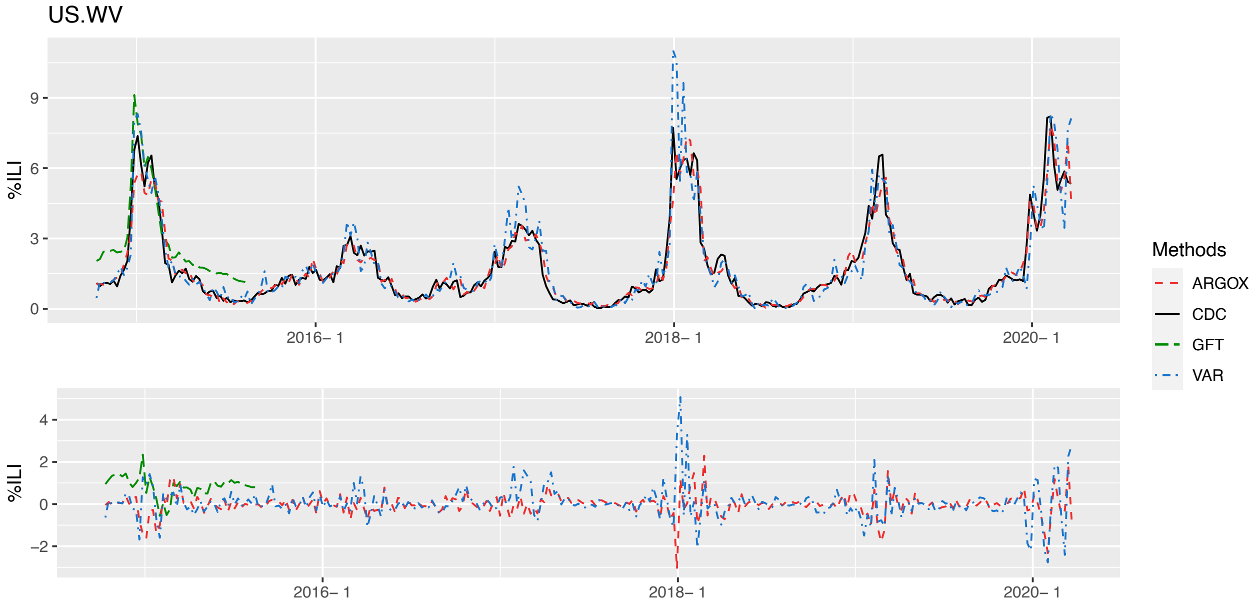

Figure S48. Plots of the %ILI estimates (top) and the estimation errors (bottom) for West Virginia (WV).

	Whole period '14-'20	Overall '14-'17	'14-'15	'15-'16	'16-'17	'17-'18	'18-'19	'19-'20
MSE								
ARGOX	**0.142**	**0.184**	**0.263**	**0.111**	**0.144**	**0.138**	**0.077**	**0.182**
VAR	0.870	0.802	1.446	0.474	0.681	1.958	0.343	0.788
GFT	–	–	0.562	–	–	–	–	–
Lu et al. (2019)	–	0.271	0.630	0.144	0.162	–	–	–
naive	0.203	0.274	0.458	0.120	0.180	0.204	0.086	0.243
MAE								
ARGOX	**0.276**	**0.320**	**0.380**	**0.260**	**0.311**	**0.300**	**0.216**	**0.328**
VAR	0.583	0.618	0.881	0.557	0.530	0.891	0.432	0.666
GFT	–	–	0.604	–	–	–	–	–
Lu et al. (2019)	–	–	–	–	–	–	–	–
naive	0.308	0.360	0.418	0.298	0.344	0.344	0.224	0.381
Correlation								
ARGOX	**0.956**	**0.928**	**0.945**	**0.752**	**0.895**	**0.951**	**0.888**	**0.974**
VAR	0.779	0.738	0.695	0.543	0.757	0.749	0.475	0.860
GFT	–	–	0.897	–	–	–	–	–
Lu et al. (2019)	–	0.898	0.868	0.696	0.895	–	–	–
naive	0.937	0.896	0.902	0.743	0.869	0.922	0.885	0.962

Table S53. Comparison of different methods for state-level %ILI estimation in Wisconsin (WI). The MSE, MAE, and correlation are reported. The method with the best performance is highlighted in boldface for each metric in each period.

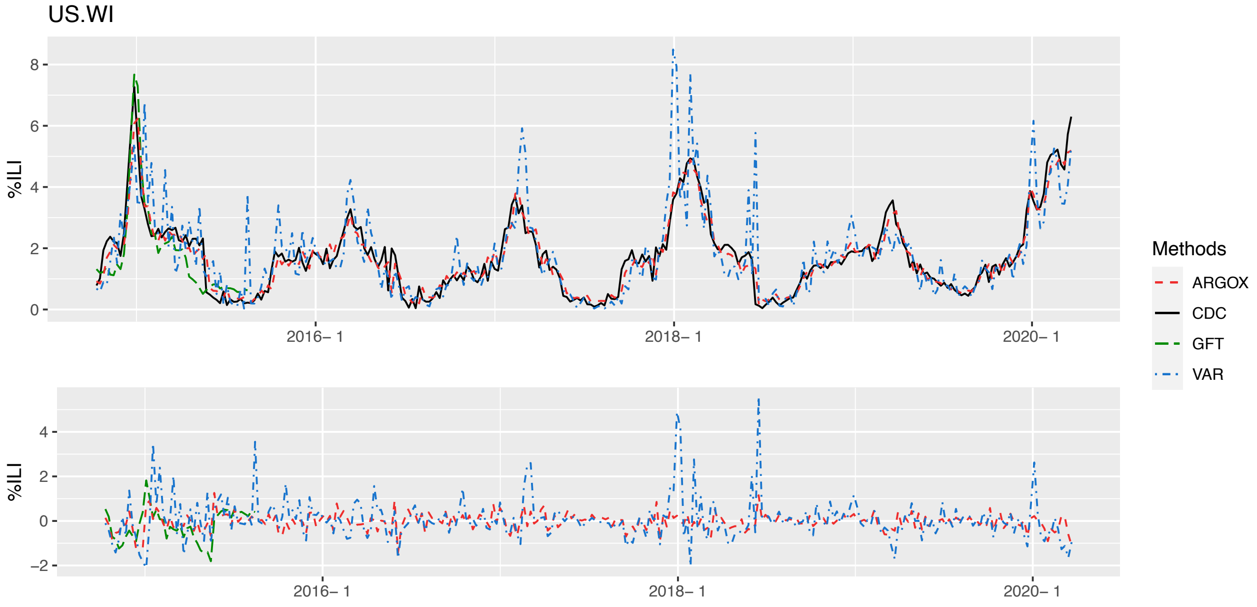

Figure S49. Plots of the %ILI estimates (top) and the estimation errors (bottom) for Wisconsin (WI).

	Whole period '14-'20	Overall '14-'17	'14-'15	'15-'16	'16-'17	'17-'18	'18-'19	'19-'20
MSE								
ARGOX	**0.276**	**0.143**	0.130	**0.128**	**0.278**	**0.468**	**0.556**	**0.912**
VAR	0.739	0.473	0.489	0.290	0.946	1.503	1.603	1.554
GFT	–	–	0.318	–	–	–	–	–
Lu et al. (2019)	–	–	–	–	–	–	–	–
naive	0.325	0.156	**0.115**	0.139	0.339	0.636	0.681	0.992
MAE								
ARGOX	**0.341**	**0.260**	0.277	**0.241**	**0.394**	**0.472**	**0.558**	0.711
VAR	0.503	0.402	0.393	0.390	0.615	0.919	0.730	0.887
GFT	–	–	0.480	–	–	–	–	–
Lu et al. (2019)	–	–	–	–	–	–	–	–
naive	0.362	0.271	**0.248**	0.265	0.448	0.585	0.593	**0.690**
Correlation								
ARGOX	**0.947**	**0.921**	0.938	**0.884**	**0.866**	**0.961**	**0.908**	**0.864**
VAR	0.880	0.846	0.930	0.728	0.740	0.848	0.862	0.803
GFT	–	–	0.891	–	–	–	–	–
Lu et al. (2019)	–	–	–	–	–	–	–	–
naive	0.939	0.918	**0.946**	0.881	0.842	0.940	0.890	0.858

Table S54. Comparison of different methods for state-level %ILI estimation in Wyoming (WY). The MSE, MAE, and correlation are reported. The method with the best performance is highlighted in boldface for each metric in each period.

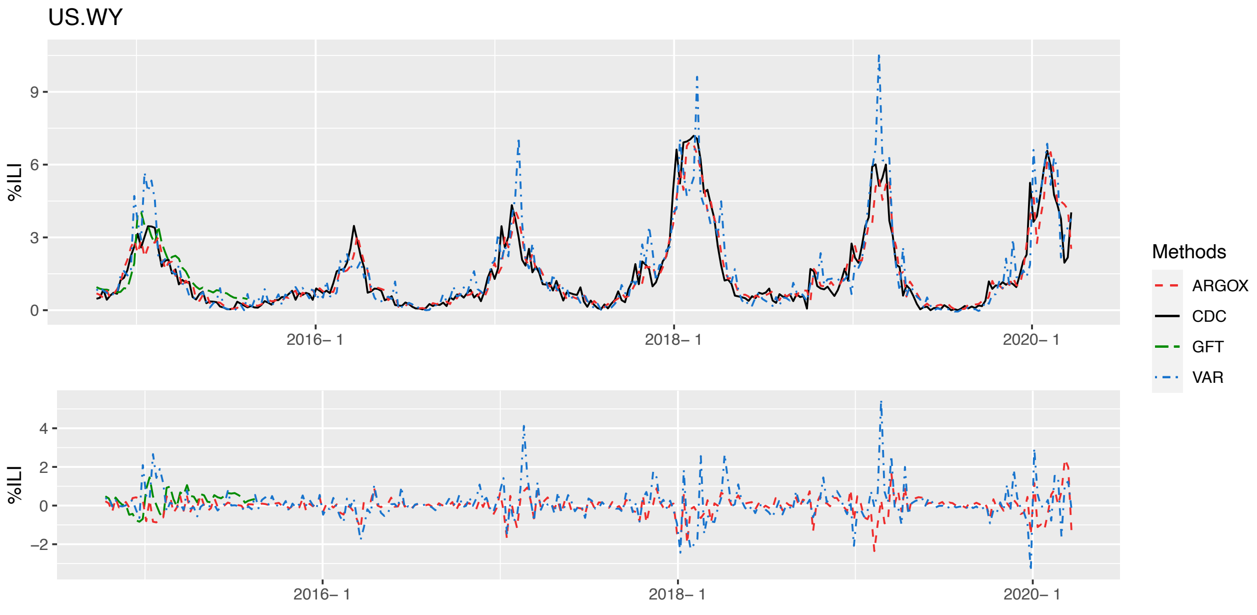

Figure S50. Plots of the %ILI estimates (top) and the estimation errors (bottom) for Wyoming (WY).

	Whole period '14-'20	Overall '14-'17	'14-'15	'15-'16	'16-'17	'17-'18	'18-'19	'19-'20
MSE								
ARGOX	**0.204**	**0.063**	**0.041**	**0.041**	**0.154**	**0.258**	**0.045**	1.599
VAR	0.242	0.105	0.080	0.097	0.187	0.339	0.187	**1.399**
GFT	–	–	0.257	–	–	–	–	–
Lu et al. (2019)	–	–	–	–	–	–	–	–
naive	0.269	0.072	0.048	0.069	0.154	0.581	0.105	1.768
MAE								
ARGOX	**0.220**	**0.178**	**0.153**	**0.172**	0.285	**0.347**	**0.167**	**0.632**
VAR	0.301	0.238	0.223	0.250	0.299	0.386	0.336	0.745
GFT	–	–	0.436	–	–	–	–	–
Lu et al. (2019)	–	–	–	–	–	–	–	–
naive	0.267	0.188	0.168	0.207	**0.273**	0.454	0.246	0.849
Correlation								
ARGOX	**0.959**	**0.956**	**0.962**	**0.956**	0.907	**0.972**	**0.971**	0.887
VAR	0.949	0.934	0.938	0.907	**0.909**	0.960	0.882	**0.898**
GFT	–	–	0.949	–	–	–	–	–
Lu et al. (2019)	–	–	–	–	–	–	–	–
naive	0.944	0.949	0.950	0.929	0.902	0.928	0.929	0.878

Table S55. Comparison of different methods for state-level %ILI estimation in New York City (NYC). The MSE, MAE, and correlation are reported. The method with the best performance is highlighted in boldface for each metric in each period.

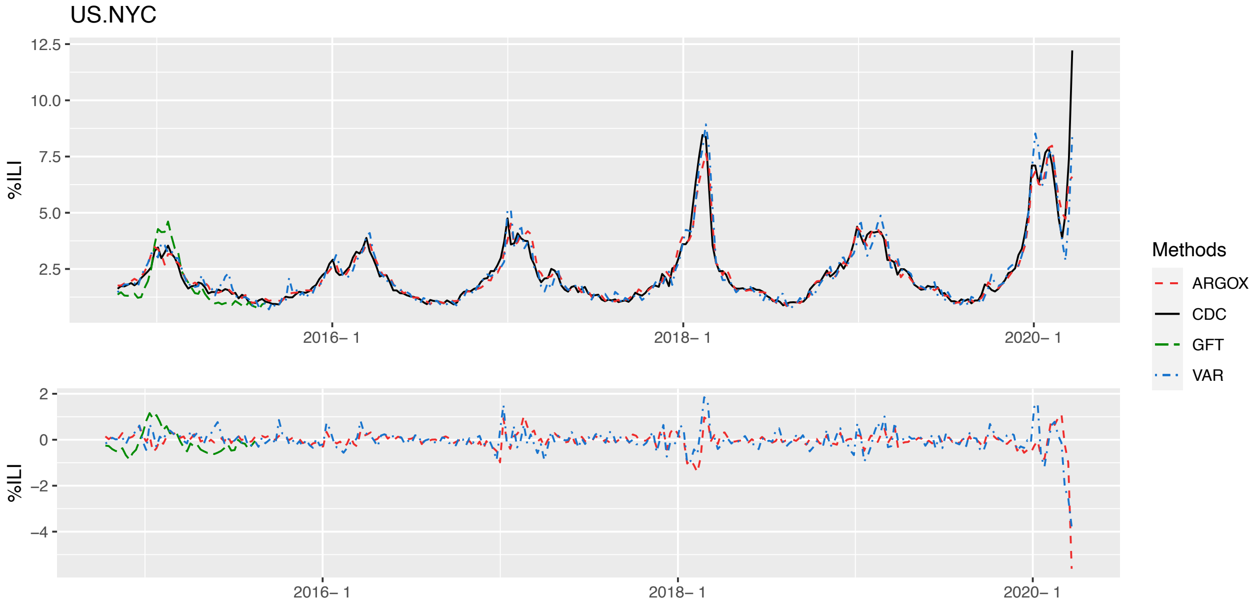

Figure S51. Plots of the %ILI estimates (top) and the estimation errors (bottom) for New York City (NYC).
